# Evaluating the effects of switching from cigarette smoking to using a heated tobacco product on health effect indicators in healthy subjects: study protocol for a randomized controlled trial

**DOI:** 10.1007/s11739-019-02090-8

**Published:** 2019-05-02

**Authors:** Nik Newland, Frazer John Lowe, Oscar Martin Camacho, Mike McEwan, Nathan Gale, James Ebajemito, George Hardie, James Murphy, Christopher Proctor

**Affiliations:** Group Research and Development, British American Tobacco (Investments) Ltd., Regents Park Road, Southampton, SO15 8TL UK

**Keywords:** Tobacco heating product, PRRP, Cardiovascular disease, Biomarkers, NNAL, Augmentation Index, Oxidative stress, Smoking cessation

## Abstract

Tobacco heating products (THPs) are a potentially safer alternative to combustible cigarette smoking. Through continued use, THPs may reduce smoking-related disease risk, whilst maintaining the sensorial experience and nicotine delivery sought by smokers. While literature evidence of the biological effects of THP aerosol exposure is increasing, there remains a knowledge gap with respect to substantiation of THP reduced risk potential in longer term real-life use. This randomized, multi-centre, controlled clinical study will test the hypotheses that following a switch from combustible cigarettes to a THP for 1 year, participants will experience a sustained reduction in exposure to tobacco-related toxicants that will lead to favourable changes in health effect indicators associated with smoking-related disease development. Changes in such indicators will be contextualized against smoking cessation and never-smoker cohorts. Up to 280 participants who do not intend to quit smoking will be randomized to continued combustible smoking (arm A, up to *n* = 80) or a commercially available THP (arm B *n* = 200). Furthermore, up to 190 participants with a high intent to quit smoking will undergo smoking cessation (arm D), and 40 never smokers will serve as a control group (arm E). Recruitment numbers were determined to be sufficient to achieve *n* = 50 in arms A, B and D, at study end. Enrolment started in March 2018 and the trial is scheduled to be completed in March 2020. Data from this study will be a valuable addition to the growing body of evidence in the field of understanding the individual and public health impact of THPs.

Clinical Trial Registration: https://www.isrctn.com/ISRCTN81075760

**Electronic supplementary material:**

The online version of this article (10.1007/s11739-019-02090-8) contains supplementary material, which is available to authorized users.

## Introduction

Cigarette smoking is a well-known cause of human disease [1]. The associated risks are known to correlate with duration of smoking and daily cigarette consumption, and quitting reduces these risks [2, 3]. Reducing the negative health burden of tobacco use is a clear public health priority and has led to a series of regulatory and educational initiatives to persuade people not to smoke [1, 4]. Despite these efforts, smoking rates in adult populations worldwide remains at 10–40% in most countries [5], and the World Health Organization (WHO) forecasts that there will be around 1.5 billion tobacco smokers worldwide in 2050 [6]. Therefore, complementing existing tobacco control policies with strategies that attempt to reduce or prevent harm in those who will otherwise continue to smoke is important. Tobacco harm reduction, the substitution of cigarette smoking for potentially reduced-risk nicotine delivery systems, could offer substantial public health gains if widely adopted [7, 8]. Tobacco researchers and policy experts have long embraced the idea that less harmful sources of nicotine could provide rewarding effects similar to that of cigarettes and might entice smokers away from cigarette smoking. This is because nicotine per se (decoupled from tobacco smoke) at doses commonly consumed is relatively safe [9, 10]. Although known for its psychoactive properties [11], nicotine is not a carcinogen [12] and does not contribute to smoking-related diseases [1].

In principle, pharmaceutical nicotine products [also known as nicotine-replacement therapies (NRTs)] that deliver “clean” nicotine (e.g. patches, gums, sprays and inhalers) can also be used for cigarette smoking substitution by virtue of their value in reducing cravings and symptoms of withdrawal in smokers. However, nicotine delivery from NRT products is relatively slow compared with smoking, their pharmacokinetic profiles hardly approximating those of cigarettes with maximum plasma concentrations typically showing a much lower and flatter pharmacokinetic profile compared to cigarettes [13, 14]. The deficient pharmacokinetic profiles, poor product appeal, absence of a typical hand to mouth action and satisfaction may explain why many smokers who attempt to quit using NRT products relapse to smoking. Realistic alternatives need to be as readily available as cigarettes are currently, competitively priced, socially acceptable and approved for regular long-term recreational use, while also strongly discouraging use of smoked tobacco [8].

Electronic inhalable vapour products (e-cigarettes) and heated tobacco products (THPs) have received most attention as long-term alternatives to smoking. This study will exclusively investigate a newly marketed THP.

Most THPs heat tobacco sticks, typically to temperatures lower than 350 °C, rather than combusting them. The lack of combustion results in significantly fewer chemical toxicants formed in the aerosol than in cigarette smoke, but nicotine is still delivered. Compared with e-cigarettes, THPs are less well characterised, but assessments of the chemical toxicants found in THP vapour has revealed significant reductions in the levels of many chemical toxicants when compared to those found in conventional cigarette smoke [15, 16]. In an independent clinical trial measuring CO levels in exhaled breath of participants using two recently marketed THPs, no CO elevations could be detected, suggesting that no combustion takes place when using these products [17]. Significant levels of nicotine in THP vapour has also been confirmed [15, 16, 18].

Several studies have investigated the potential for adverse human health effects of THPs, and in some cases, in comparison with combustible cigarettes. The studies performed to date have predominantly measured chemical emissions [15–21], in vitro endpoints [15, 22–25], in vivo endpoints [26–29] and biomarkers of exposure (BoE) of chemical toxicants found in cigarette smoke in individuals who have switched from smoking to using THPs [30, 31]. In summary, significant reductions in emissions, biological activity and human exposure respectively were rapidly attained and were sustained for at least 1 month of continued use [15, 16, 19]. However, the presence of some emission toxicants, biological activity and elevated BoEs remained, indicating that use of THPs is unlikely to be risk free.

With the substantially altered chemical profile of THP aerosol and the reduced chemical exposure compared with cigarette smoke, long-term negative health effects might also be reduced [32, 33]. Substantiation of the potential for risk reduction from THP use requires quantification in carefully designed clinical studies [33]. As part of premarketing authorisation applications for novel tobacco products, some regulators require clinical data to identify any potential reduction in individuals’ risk relative to continued smoking. To substantiate harm reduction potential, an extensive weight-of-evidence-based scientific dataset is likely needed. We have recently proposed an assessment framework describing the types of studies, which are likely to be needed to generate such a weight-of-evidence [34]. In addition to the datasets already published as part of the framework [35], this study will provide critical information from a real-life setting to determine if the previously characterised reductions in emissions, exposure and in vitro responses, translate into a reduced risk benefit for consumers.

The purpose of this study is twofold:

1. To test the hypothesis that reductions in toxicant delivery from a THP can translate to sustained reduction in human exposure to cigarette smoke toxicants, as assessed by measuring biomarkers of exposure in an ambulatory setting.
2. To test the hypothesis that reduction in exposure to toxicants will cause changes in health effect indicators when smokers switch to using THPs compared with smokers who continue to smoke (CTS), and that these changes are directionally similar to changes seen in smokers who undergo smoking cessation, over a period of 6–12 months in an ambulatory setting.

## Methods

The full study protocol is provided in the supplementary information, submitted with this manuscript. Due to the removal of arm C from the study, study arm names (A, B, D, E) in this manuscript have been maintained to align with the protocol and the requirements of GCP. The key aspects of the protocol are summarised below.

### Study design and participants

**Fig. 1:**
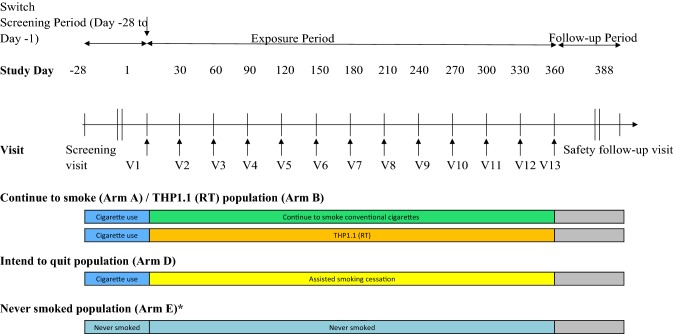
Study schematic. *Subjects in arm E will only attend screening, visit 1 (day 1), visit 4 (day 90 ± 3 days), visit 7 (day 180 ± 2 weeks), visit 13 (day 360 ( ± 2 weeks), and follow-up

This will be a multi-centre, unblinded, controlled ambulatory study with a randomised design (Fig. 1). This study will be conducted in accordance with consensus ethical principles derived from the Declaration of Helsinki, as well as with the quality standards of Good Clinical Practice. This study has been registered at the following site: https://www.isrctn.com/ISRCTN81075760.

The planned duration is 360 days, and three separate populations will be recruited, to four study arms:

- Current smokers, defined as adult male and female regular smokers of 10–30 non-mentholated commercially manufactured filter cigarettes and/or roll your own cigarettes per day. This population is further split into participants who will continue to smoke their own brand of cigarette for the duration of the study (arm A), and participants who will completely switch to THP1.1 (RT) after baseline for the duration of the study (arm B).
- Current smokers intending to quit, defined as adult male and female regular smokers of 10–30 non-mentholated commercially manufactured filter cigarettes and/or roll your own cigarettes per day intending to cease all non-medicinal nicotine use (arm D).
- Never smokers, defined as having smoked < 100 cigarettes in their lifetime and none in the 30 days before screening (arm E).

### Inclusion criteria

Full inclusion criteria are listed in the study protocol (see supplementary information), and include

- Age 23–55 years.
- Body mass index (BMI) 17.6–32.0 kg/m^2^ and body weight > 50 kg (men) or 40 kg (women).
- Good general health (in the opinion of the Principal Investigator or qualified designee).
- Willingness to refrain from consuming alcohol 24 h before study screening and all study visits.
- Participants in arms A and B must have urine cotinine levels > 200 ng/mL and exhaled breath CO levels ≥ 7 ppm at screening [36].
- Participants in arms A, and B will have smoked for at least five consecutive years prior to screening.

### Exclusion criteria

Full exclusion criteria are listed in the study protocol (see supplementary information), and include

- Acute illness (e.g. upper respiratory tract infection) requiring treatment within 4 weeks before visit 1 (participants with viral infections that resolved ≥ 2 weeks before visit 1 will be eligible for inclusion).
- History of alcoholism or drug/chemical abuse within 24 months before screening or a positive alcohol breath test (confirmed by repeat) at screening or visit 1.
- Carriers of hepatitis B virus, hepatitis C virus or HIV.
- Use of prescription or over-the-counter bronchodilator medication (e.g. inhaled or oral β-adrenergic agonists) to treat a chronic condition within the 12 months before visit 1.
- Use of any medications or substances (other than tobacco) that interfere with the cyclo-oxygenase pathway (e.g. anti-inflammatory drugs including aspirin and ibuprofen) or strong inducers or inhibitors of cytochrome CYP enzymes within 14 days or five half-lives of the drug (whichever is longer) within 14 days before visit 1.
- Strenuous physical activity within 7 days before screening or visit 1.
- Clinically relevant abnormal findings in medical history or on physical examination (e.g. presence of on-going disease or pathology).
- For participants in arms A, B, and D, use any nicotine or tobacco product other than commercially manufactured filter cigarettes and/or roll your own cigarettes within 14 days of screening.
- In arms A and B, self-reported non-inhalers (smokers who draw smoke from the cigarette into the mouth and throat but who do not inhale).
- In arms A and B, smokers planning to quit in the next 12 months at screening, although smokers who are enrolled are free to quit smoking and withdraw from the study at any time and will be directed to appropriate stop smoking services.

### Group allocation and randomisation

Current smokers will be randomly assigned to a group that will continue to smoke commercially manufactured filter cigarettes and/or roll-your-own cigarettes (arm A) or a group that will switch to THP1.1 (RT) (arm B). A randomisation scheme for arms A and B will be provided to the study sites. Randomisation lists will be separated by sex and age categories. Each site will recruit a similar number of men and women which will be randomised by two different age groups: aged 23–40 years and 41–55 years. Random allocation of participants will be monitored throughout the study. If an imbalance is seen that is anticipated to lead to problems when interpreting the data, sites will be requested to try to prioritise the recruitment of participants in the relevant category or categories.

Participants intending to quit will be allocated to arm D and non-smokers to arm E. Although these arms will not be randomised, sites will endeavour to balance these populations during recruitment by sex and the same age groups. Subjects in arm D will determine their smoking cessation strategy with the Investigator or their appropriately trained designee at visit 1. If necessary, subjects will be provided with nicotine replacement therapy (Invisipatches starting at 25 mg and weaning down ± an inhalator) or Varenicline, but a combination of this and NRT was not permitted, it was one or the other) or given a prescription for these products. Participants used these products for 12 weeks, or in some cases longer (up to 24 weeks), under the supervision of a qualified general practitioner.

For additional support, subjects will be referred to the following services based on where they live:

- Subjects in England—National Health Service (NHS) quitting support website (https://www.nhs.uk/live-well/quit-smoking/nhs-stop-smoking-services-help-you-quit), the Smokefree helpline (Tel: 0300 123 1044), online advisor support, local stop smoking service, and Smokefree app.
- Subjects in Northern Ireland—Want2Stop quitting support website (www.want2stop.info).
- Subjects in Wales—NHS Wales quitting support website (www.helpmequit.wales) and telephone helpline (0800 085 2219).

Subjects will also be provided with a 24-h site number that they contact for cessation support if required. The Investigator or their appropriately qualified designee will review subject’s progress and strategy at each clinic visit.

### Compliance

Product use compliance is a critical part of this study, as failure to fully replace cigarettes with the THP product would reduce or nullify the expected biomarker changes that would be observed if the product is used as indicated. Participants will be instructed of the importance of adhering to their randomised product allocation (arms A and B) or quitting strategy (arm D) and of not smoking (arm E). They will be asked to report any non-compliance via a study diary and will be informed that assessments of adherence will be conducted at selected clinic visits. Analysis of blood *N*-(2-cyanoethyl)valine haemoglobin adducts (CEVal; formed following acrylonitrile exposure) will be conducted on all study participants, as a marker of combusted tobacco exposure. We will use different thresholds for CEVal in ancillary analyses to deduce product use compliance. These thresholds have been calculated based on a previous study where this biomarker was reported for a modified combustible prototype cigarette [37].

### Study product

The THP1.1 (RT) is a novel THP that has been designed and manufactured by British American Tobacco (BAT), which is commercially available in Japan, Italy, Canada, Romania, South Korea, Russia, and Switzerland. Product information for THP1.0 (the same heating device, and a similar consumable) is fully described by Eaton et al. [38], and the associated machine-puffed chemical emission data are described by Forster et al*.* [16]. THP1.1 (RT) has two components: a cylindrical tobacco stick consumable of 83 mm length and 5.4 mm diameter [weight 540 mg of which 210 mg is tobacco, and nicotine yield is 0.68 mg per stick (modified Health Canada Intense puffing regime)], and an electronic heating device into which the consumable is inserted for heating before use. The heating device comprises a rechargeable battery, an electrical heating element and electronic hardware that controls device warming, heating temperature and heating period. The device heats the tobacco to a maximum temperature of 240 °C ± 5 °C [38], and contains safety technology to prevent heating over 260 °C.

**Fig. 2:**
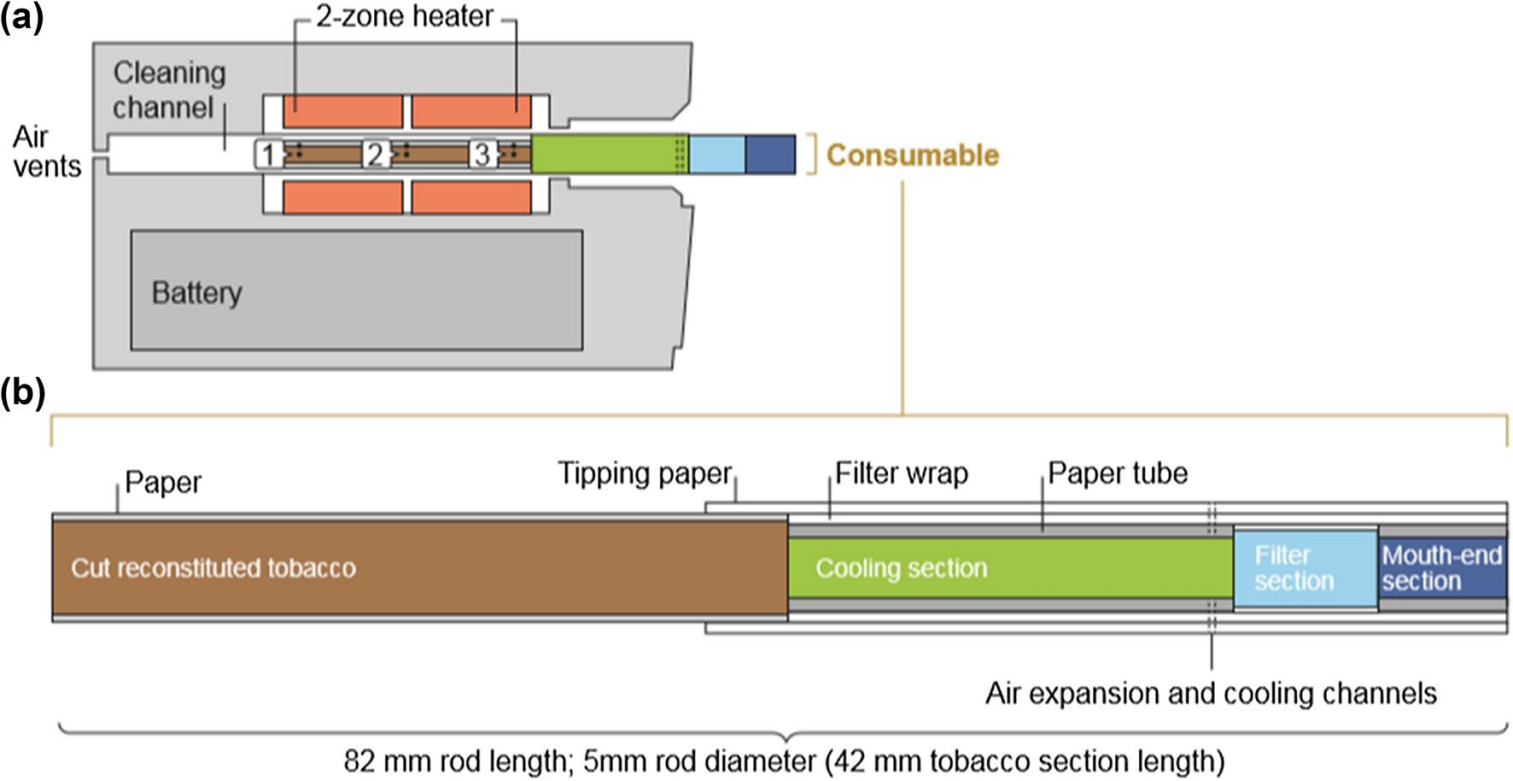
**a** The THP1.1 (RT) tobacco heating product and **b** the THP1.1 (RT) consumable

To use THP1.1 (RT), the consumable is placed in a port on the top of the THP device, leaving the filter end protruding. The port is accessed by moving a sliding cover (Fig. 2). Once the consumable is inserted, the user pushes and holds an activation button on the THP for 3 s to start heating process; the THP vibrates briefly and one of a series of four LED lights on the front of the device around the activation button illuminates to indicate this has begun. The remaining lights illuminate, and when the final light is lit, and the device vibrates again, heating is complete and the user many begin puffing. The heating process takes 40 s and heating at the maximum temperature continues for 210 s, after which the heater shuts down automatically.

### Product use

Participants will be asked to continue smoking their usual brands of cigarette until randomisation (arms A and B) or enrolment (arm D). After randomisation, the participants in arms A and B will be allowed to consume only the assigned products until the final visit on day 360. Smoking or THP use will be ad libitum but capped at 200% of self-reported cigarette per day (CPD) consumption at screening. Participants in arm A will be told before the study that they will continue to smoke their usual brand of cigarettes at their own expense. Smokers will be reminded of the risks associated with smoking prior to enrolment, and informed that they are free to voluntarily quit smoking and/or withdraw from the study at any time.

Participants in arm D will be allowed to use only specific NRT and smoking cessation aids from enrolment to day 360. For participants in arm B, at visit 1, they will be provided with the THP1.1 (RT) and stick consumables equivalent to no more than 150% of the self-reported number of cigarettes per day (CPD) consumed at screening, with the possibility of more, up to a total of 200% of original CPD consumption, before visit 2 by visiting the study site. At visits 2–12, product usage will be assessed by return of all empty, part-used, and unused packs of THP consumables, and the next batch of product will be supplied at 120% of usage in the previous period, up to the limit of 200% of pre-screening consumption. At visit 13, as well as all empty, part-used and unused packs of THP consumables, participants will be asked to return the study device, chargers and other accessories supplied for use in this study.

Two devices are supplied to study participants to mitigate the potential for device loss and/or failure/malfunction. Furthermore, a spare device allows the subject to continue use of the product when required, while a depleted battery is recharging. In the event of loss, or a failure of a device (damage or malfunction), subjects are supplied with a telephone contact at the clinic, who will immediately arrange a replacement device.

### Study objectives and endpoints

The primary objective of the study is

- To quantitatively assess differences in primary study endpoints at 90, 180, and 360 days between subjects who continue to smoke conventional cigarettes and subjects who switch to a THP.
  Secondary objectives of the study are as follows:
- To quantitatively assess differences in secondary study endpoints between subjects who continue to smoke conventional cigarettes and subjects who switch to a THP.
- To assess differences in all study endpoints between subjects who switch to a THP and subjects in the assisted smoking cessation arm.
- To assess the differences in all study endpoints between subjects who switch to a THP or undertake assisted smoking cessation, and subjects who have never smoked.
- To monitor the safety profile of subjects using THPs and combustible cigarettes, and subjects in the smoking cessation and never-smoker arms.

Exploratory objectives are as follows:

- To quantitatively assess the time required to observe changes in selected primary and secondary endpoints following a switch from conventional cigarettes to a THP or assisted cessation.
- To quantitatively assess differences in the exploratory endpoints between subjects who continue to smoke conventional cigarettes, subjects who switch to a THP, subjects in the cessation arm, and subjects who have never smoked (to be reported separately).
- To investigate the profile of selected primary and secondary endpoints over the course of the study for each study arm.

The endpoint analysis schedule is presented in Table 1, and the study endpoints are shown in Tables 2 and 3.

**Table 1:** Analysis schedule

Analysis schedule	Study visit	Study timepoint
1	All visits	All days
2	1, 2, 3, 4, 7, 10, 13	0, 30, 60, 90, 180, 270, 360
3	1, 4, 7, 10, 13	0, 90, 180, 270, 360

**Table 2:** Primary endpoint biomarkers

Biomarker	Abbreviation	Matrix	Indication	Analysis Schedule
Total 4-(methylnitrosamino)-1-(3- pyridyl)-1-butanol	Total NNAL	Urine (24 h)^a^	Metabolite of the smoke toxicant 4-(methylnitrosamino)-1-(3-pyridyl)-1-butanone (NNK)	2
8-epi-Prostaglandin F_2α_ type III	8-Epi-PGF_2α_ type III	Urine (24 h)^a^	Marker of oxidative stress	2
Augmentation Index	AIx	Physiological measure	Marker of arterial stiffness and hence an indicator for cardiovascular risk	2

^a^Ambulatory collection

**Table 3:** Secondary endpoint biomarkers and measures

Biomarker of exposure	Abbreviation	Associated toxicant	Matrix	Analysis schedule
Carbon monoxide	CO	Carbon monoxide	Exhaled breath	1
Total nicotine equivalents (nicotine, cotinine, 3-hydroxycotinine and their glucuronide conjugates)	TNeq	Nicotine	Urine (24 h)^a^	2
Total *N*-nitrosonornicotine	Total NNN	NNN	Urine (24 h)^a^	2
3-Hydroxypropylmercapturic acid	3-HPMA	Acrolein	Urine (24 h)^a^	2
3-Hydroxy-1-methylpropylmercapturic acid	HMPMA	Crotonaldehyde	Urine (24 h)^a^	2
*S*-Phenylmercapturic acid	S-PMA	Benzene	Urine (24 h)^a^	2
Monohydroxybutenyl-mercapturic acid	MHBMA	1,3-butadiene	Urine (24 h)^a^	2
2-Cyanoethylmercapturic acid	CEMA	Acrylonitrile	Urine (24 h)^a^	2
1-Hydroxypyrene	1-OHP	Pyrene	Urine (24 h)^a^	2
2-Hydroxyethylmercapturic acid	HEMA	Ethylene oxide	Urine (24 h)^a^	2
4-Aminobiphenyl	4-ABP	4-aminobiphenyl	Urine (24 h)^a^	2
2-Aminonaphthalene	2-AN	2-aminonaphthalene	Urine (24 h)^a^	2
Ortho-toluidine	o-Tol	Ortho-toluidine	Urine (24 h)^a^	2

Biomarker of compliance	Abbreviation	Indication	Matrix	Analysis schedule
*N*-(2-cyanoethyl)valine (haemoglobin adduct)^b^	CEVal	Acrylonitrile	Whole blood	2

Biomarker of potential harm (BoPH)	Abbreviation	Indication	Matrix	Analysis schedule
Nitric oxide	NO	Bronchodilation/vascular tone	Exhaled breath	2
11-Dehydrothromboxane B2	11-dTx B2	Platelet activation/coagulation	Urine (24-h)^a^	2
4-Hydroxy-nonenal + metabolites	4-HNE	Oxidative stress	Urine (24-h)^a^	2
White blood cell count	WBC count	Inflammation	Whole blood	2
Monocyte chemotactic protein 1/ C–C motif chemokine ligand 2	MCP-1/CCL2	Inflammation/chemokine	Plasma	3
Soluble intercellular adhesion molecule-1	s-ICAM1	Endothelial dysfunction	Plasma	3
Fibrinogen	Fib	Coagulation	Plasma	3
High-sensitivity C-reactive protein	hsCRP	Inflammation	Plasma	3
Homocysteine	HMCys	Oxidative stress	Plasma	3
Glucose	Gluc	Metabolic status	Plasma	3
Plasminogen activator inhibitor-1	PAI-1	Coagulation	Plasma	3
Tissue plasminogen activator	tPA	Coagulation	Serum	3
E-selectin	SELE	Endothelial dysfunction	Plasma	3
Endothelin-1	ET-1	Vascular tone/endothelial dysfunction	Serum	3
3-Nitrotyrosine	3-NTyr	Nitrosative stress/vascular tone	Plasma	3
Serum lipids (high-density lipoprotein [HDL], low-density lipoprotein [LDL], total cholesterol, triglycerides)	HDL/LDL/CholTotal/Trigly	Metabolic status	Serum	2

Other measures				
Creatinine (urine; 24-h)^b^				2
Physiological measures				
Body weight/waist circumference				3
Carotid/femoral pulse wave velocity				2
6-Minute walking test				2
Finger plethysmography				3
Questionnaires				
Fagerström test for cigarette dependence (FTCD)^b^ [39]				3
Product satisfaction^b^				V4, V7, V10, V13
Smoking cessation quality of life^b^ [40]				3
Self-reported product use (eDiary/paper diary)^b^				Throughout study
Cough and Shortness of Breath VAS^b^				3
Safety				
Physical examination^b^				2
Vital signs^b^				3
Electrocardiogram (ECG)^b^				3
Clinical laboratory evaluations^b^				3
Lung function test (spirometry)^b^				
Peak flow				2
Forced vital capacity (FVC)				2
Forced expiratory flow (FEF) 25–75%				2
Forced expiratory volume in 1 s (FEV_1_)				2
Adverse events (AE)/SAE recording^b^				Throughout study

^a^Ambulatory collection

^b^With the exception of blood pressure that will be measured as part of vital signs, endpoint will not be assessed using the statistical analysis as described above, but may be analysed using a statistical method that will be described in the statistical analysis plan

Additional exploratory biomarker endpoints of interest will be body fat, tetrahydrobiopterin/dihydrobiopterin (BH4/BH2) ratio in plasma, untargeted and targeted transcriptomics (assessed in nasal epithelial cells and white blood cells), targeted lipoprotein levels (by nuclear magnetic resonance; NMR) in serum and targeted metabolomics in serum.

A detailed schedule of assessments can be found in the supplementary information.

### Statistical power considerations

Statistical power calculations based upon the primary endpoint that required the largest sample size to observe change [Augmentation Index (AIx)] determined that 50 participants completing the study in arms A, B, and D will provide sufficient power to allow multiple between-arm comparisons between the test and control products. Specifically, the power calculation was based on the number of participants required to perform a contrast based on the *F* statistic, with 90% power between the arm B (THP) and arm A (continued smoking) at day 360. The sample size was determined to be adequate based on AIx with expected means of 25.7% and 17.5% for smoker and THP arms, respectively, and a common standard deviation of 12.4% [41]. This calculation assumes an 80% change from baseline with the THP, in line with that observed in participants quitting smoking and an alpha level of 0.0451, adjusted for timepoint multiplicity using O’Brien–Fleming sequential approach [42]. Thus, recruitment targets are 80 participants to arm A, 200 to arm B and 190 to arm D. In arm E, we have determined a sample size of 30, empirically with no formal statistical analysis, as this cohort’s biomarker profile is expected to be stable throughout the study.

### Statistical analysis of primary and secondary objectives

The primary objective will be examined by computing levels of biomarkers at each timepoint, i.e. baseline, 90, 180, and 360 days. These data will be compared between the THP arm (arm B) and the continued smoking arm (arm A) using specific contrast tests from statistical models adjusted for baseline measurements. Data will be examined and may be transformed to ensure that any assumptions associated with statistical tests or models are obeyed. Alpha level across timepoints has been adjusted using the O’Brien–Fleming approach, with 0.0471 overall available alpha at day 360, 0.0151 at day 180, and 0.0006 at day 90 [42]. The significance level has been allocated at each primary endpoint based on likelihood of success to detect a significant change in biomarker levels. At day 360, statistical comparisons between THP and control will be performed at *α* = 0.0469 for AIx. The remainder alpha will be distributed equally (*α* = 0.0001) for the other two primary endpoints. At day 90, only changes in BoE are expected; therefore, only total NNAL will be statistically assessed with *α* = 0.0006. For the statistical analysis performed at day 180, an overall *α* level of 0.0151 will be equally distributed between the three primary endpoints (0.00503). If any endpoint were to be significant at day 90 or 180, it will not be statistically assessed at day 180 and/or 360, as appropriate, and its assigned alpha level will be equally distributed between the remaining primary endpoints.

Similarly, biomarker measures in the secondary objectives will be examined by computing levels of biomarkers at each timepoint. These data will be compared between the THP and the main control arm using specific contrast tests from statistical models adjusted for baseline measurements. Statistical comparisons for secondary endpoints will be only performed if any of the primary endpoints is significant and using the alpha level released by primary endpoints. If the statistical comparison for total NNAL is significant at day 90, only the secondary BoE will be analysed at day 90 using a 0.0006 significance level. Multiplicity adjustment for family-wise error of secondary endpoints will be performed using Holm’s method. If any secondary endpoint were to be significant at day 90 or 180, it will not be statistically assessed at day 180 and/or 360, as appropriate.

Product use compliance is a critical part of this study, as failure to fully replace cigarettes with the THP product would reduce or cancel the expected biomarker changes that would be observed if the product is used as indicated. To aid compliance assessment we will use a haemoglobin adduct of acrylonitrile; *N*-(2-cyanoethyl)valine (CEVal). Acrylonitrile is below the detection limit in the THP product emissions but can be found in cigarette smoke. We will use different thresholds for CEVal in ancillary analyses to deduce product use compliance. These thresholds have been calculated based on a previous study where this biomarker was reported for a modified combustible prototype cigarette [43].

The interim analysis on day 90 will be performed on a subset of subjects who were enrolled in the study on or before the day that the 42nd subject was enrolled on to arm A, and were still enrolled in the study at day 90. This was chosen to ensure that 30 subjects on arm A were still enrolled on the study at day 90 to give sufficient power to detect the statistical difference between the two arms for total NNAL.

All safety data will be summarised for all safety parameters based on the safety population, stratified by study arm.

## Expected results

Enrolment started in March 2018 and the trial is ongoing. The results of this study are expected in 2020.

## Discussion

Scientific studies on next-generation tobacco and nicotine products are increasing in number recently; however, there is still a substantial gap in knowledge with respect to the longer term health effects associated with their use. While there are now numerous studies assessing the biological effects of electronic cigarettes, datasets relating to THPs are still very limited, and more information is needed to inform consumers, public health and regulation alike. This study will be the first to our knowledge to longitudinally investigate the exposure and health effects associated with THP use over a 12-month period and, furthermore, compare those data to that of assisted smoking cessation and continued combustible product use over the same period of time. The ambulatory design of this multi-centre study will facilitate observation of exposure and health effects associated with real-life use of the THP, hence a key critical challenge to the success of the study will be participant compliance with exclusive product use.

### Study participant compliance

Compliance to the study protocol is critical to achieve the scientific objectives of the study. Studies which utilise clinical confinement are somewhat easier to monitor in terms of compliance, as clinic staff are constantly on hand to ensure participants adhere to protocol. However, in an ambulatory setting where participants continue their everyday lives away from the clinic, controls and monitoring tools must be used to ensure, to the greatest degree possible, that participants adhere to the protocol. While some areas of compliance are easily monitored, and breaches easily detectable (e.g. attendance of study visits), others are less so, and a great deal of reliance is placed upon self-reported information from study participants, which may, or may not be 100% complete or accurate.

A key area of compliance for this study is the adherence to exclusive product use and smoking cessation (for the relevant groups) throughout the full study duration. With respect to smoking cessation, self-reported diaries are usually employed to track cigarette use throughout a trial and can provide insight into whether or not a participant who is on a smoking cessation protocol is continuing to smoke cigarettes. However, participants in clinical trials such as this often fail to fill out self-reported diaries correctly, accurately or even at all. Enthusiasm and compliance at the very start of such a trial are likely to be high. But within the early stages of the study, enthusiasm and compliance have been shown to wane significantly, along with effective selfreporting [44]. Clinical staff who interact with participants at each study visit are limited in their ability to monitor such compliance. Furthermore, they are especially hampered when separate study visits span several months/years, as the opportunity to review diary compliance and subsequently encourage non-compliant participants to return to compliance is limited by the visit schedule. Often, the lack of information in self-reported diaries from missed entries may undermine confidence in the data, and hence the overall study conclusions. Therefore, utilization of improved and more objective measures of adherence is needed to protect the accuracy of the data and, in turn, the overall conclusions of the study. In this study, a combination of different strategies will be employed to ensure the highest level of compliance.

1. Electronic diaries
  In this study, participants will self-report product use at each study visit, via the use of diaries. To mitigate against missed information, we have provided participants with an electronic diary application which can be accessed via a mobile phone or tablet. The electronic diary will prompt users to complete the diary and inform the clinic staff when entries have been missed. Clinic staff will then be able to make follow-up calls to provide support and assistance if required.
2. Product counting
  A measure of overall compliance to product use will be evaluated by counting all empty, part-used, and unused packs of THP consumables returned to investigators.
3. Biomarker of compliance
  Given that elimination half-lives of most BoEs are relatively rapid (days/weeks) and are under investigation in this study, further biomarkers are needed to monitor product compliance over a longer period of time. For this, we will use the blood-borne haemoglobin adduct, CEVal, which is formed following exposure to acrylonitrile, a major tobacco smoke constituent. The approximate half-life of this biomarker is 120 days, as it mirrors the life cycle of red blood cells in vivo [37].

### Study design

The protocol incorporates a number of innovative approaches that contribute to the specific uniqueness and quality of the study.

Participants in this study will be a minimum of 23 years of age, which is 18 years (the legal age to obtain tobacco products in the United Kingdom) plus a smoking history of at least 5 years. The study will also ensure that a wide selection of ages is investigated in the study by recruiting individuals between 23 and 55 years old. Given that some of the health effect indicators (such as AIx) are known to correlate with age [45], randomisation has been set to ensure that age bias is minimised, and an equal balance of younger and older participants is recruited. Furthermore, individuals older than 55 years of age are excluded due to the increased likelihood of the presence of clinical or sub-clinical disease.

Smokers are defined as “adult male and female regular smokers of 10–30 non-mentholated commercially manufactured filter cigarettes and/or roll your own cigarettes per day”. These limits have been set due to the presence of significant differences in cohorts of smokers who smoke less than and greater than 10 cigarettes per day for some of the biochemical endpoints.

To investigate the effects of smoking cessation over 12 months, a population of subjects who are intending to quit smoking will be recruited, thus maximizing the proportion of these subjects completing the study. The inclusion of a smoking cessation arm in this study is critical to provide context to any favourable changes observed when participants switch from smoking to the THP. Furthermore, the cessation arm will also demonstrate the best possible outcome within the timeframe of the study, in terms of the study endpoints, and the extent to which they return to levels observed in never-smokers.

Where BoEs have been shown to change relatively rapidly (days/weeks), health effect indictors such as BoPHs require more time (weeks/months) to observe meaningful change following smoking cessation [46]. Thus, the study period of 12 months is expected to provide sufficient time to allow smoking cessation-responsive BoPHs to change in a favourable manner. Furthermore, as several BoPHs have been shown to change in shorter periods of time, the length of this study will also allow the opportunity to observe if any favourable changes are sustained, and whether or not they return to levels observed in never-smokers.

### Health effect indicators

The health effect indicators selected for this study include a combination of biofluid and physiological analysis for biomarkers of potential harm (BoPH) and health assessment questionnaires. The BoPH included in this study were selected based upon their propensity to change in healthy individuals following smoking cessation.

The BoPH selected for evaluation include a suite of biomarkers that are traditionally associated with cardiovascular/respiratory disease risk (e.g. HDL/LDL cholesterol, fibrinogen and spirometry assessments) [46]. To complement these, numerous biomarkers have been selected which indicate change in earlier mechanisms involved in the development of cardiovascular and respiratory disease. For example, hypertension is a known risk factor for future myocardial infarction and stroke [47], and the measurement of systolic and diastolic blood pressure over time, can provide insight into the likelihood of the future development of hypertension [47]. To augment insight into the factors modulating changes in blood pressure, a suite of BoPHs has been included to monitor cause–effect relationships at the molecular, cellular and tissue levels of biological hierarchy, which are reported to be mechanistically involved in the modulation of blood pressure over time. Such BoPH provide insight into the level of vascular oxidative/nitrosative stress (a key driver of smoking-related vascular disease) [48], nitric oxide bioavailability (a key mediator of vasodilation and vascular tone) [49] and arterial stiffness [50]. Further BoPH will be measured which relate to platelet activation, coagulation, inflammation, cell adhesion, endothelial dysfunction and metabolic status. Finally, ‘omic approaches’ will be utilised to assess holistic changes over time in biological samples of respiratory and circulatory origin, and health-related questionnaires will be utilised to provide insight into more subjective perceptions of health and quality of life over time.

In concert, these tools will provide much needed insight into the early processes involved in the development of smoking-related diseases, and better characterise the health-related improvements associated with smoking cessation. With the BoPH profiles of continued smoking, smoking cessation and never smoking characterised, the THP 1.1 (RT) data will be analysed to determine if its exclusive use facilitates a BoPH profile similar to that of smoking cessation, or not.

### Study limitations

Compliance in a study substantiating risk reduction when switching from combustible cigarettes to THPs is paramount. The appeal and the overall sensorial experience of the product under investigation could be a limiting factor to this extent. Exclusive use of the THP product and 100% adherence to smoking cessation protocols are notoriously difficult to both monitor and achieve. Long-term acceptability and tolerability data for the THP are not yet available and, therefore, it is possible that participants using the THP will continue to use combustible cigarettes to varying degrees in parallel. While the compliance tools that are included in the study will certainly help to identify such “dual-use” participants, they will not be perfect.

The study participants will be recruited from the UK population alone; therefore, the conclusions of the study may not be reflective of populations from other geographical locations and cultures. Furthermore, the study population will consist of healthy adult volunteers aged 23–55 years of age, hence the data may not be reflective of the potential effects of THP use in populations with clinical disease or other vulnerable populations, or older and younger individuals.

Although the study period of 12 months is expected to provide sufficient time to detect smoking cessation-responsive BoPH changes, it is still possible that for some of them, a much longer follow-up period might be required.

Generalisation of the study data to other THPs should be undertaken with extreme caution. While THPs share a common feature in heating tobacco, rather than burning it, the product heating devices are often designed very differently within the category. Furthermore, the consumables also share significant differences in physical characteristics and ingredients. Taken together, such differences have the potential to yield very different chemical emission profiles, use behaviours and ultimately, biological effects and disease risk. That said, there is potential for bioequivalence exercises to extrapolate the data to similar products where it is appropriate to do so (i.e. under suitable criteria demonstrating that the original and variant products are sufficiently similar to conduct such an exercise, and for a specific regulatory purpose). Such exercises are likely to require additional data packages to support product similarity.

## Summary

Where reductions in chemical emissions and human toxicant exposure have already been demonstrated with THP use in the short-term, the longer-term effects of THP use in the general population remain unknown. Data from this study will be a valuable addition to the growing body of evidence in the field of understanding the public health impact of THPs. It will generate a pioneering dataset from which the public health community, regulators and consumers can gain insight into whether or not THPs provide a viable and safer alternative to combustible cigarette smoking in adult populations who chose to smoke, or are unable to quit smoking.

## Electronic supplementary material

Below is the link to the electronic supplementary material.

Supplementary file1 (DOCX 297 kb)

Supplementary file2 (DOCX 98 kb)

Supplementary file3 (DOCX 61 kb)
