# Supplementary material for: Evaluating the effects of switching from cigarette smoking to using a heated tobacco product on health effect indicators in healthy subjects: study protocol for a randomized controlled trial
Source: Intern Emerg Med. 2019 May 2;14(6):885–98. doi: 10.1007/s11739-019-02090-8 (PMC6722146; doi:10.1007/s11739-019-02090-8)
Supplement: Supplementary file 1 — Supplementary file1 (DOCX 297 kb) [file 11739_2019_2090_MOESM1_ESM.docx]

Clinical Study Protocol

A randomised, controlled study to evaluate the effects of switching from cigarette smoking to using a Tobacco Heating Product on health effect indicators in healthy subjects

Clinical Trial Registration : <https://www.isrctn.com/ISRCTN81075760>

Final Amendment (Version 9)

25 January 2019

TABLE OF CONTENTS

[TABLE OF CONTENTS 2](#_Toc534965046)

[LIST OF ABBREVIATIONS 6](#_Toc534965047)

[DEFINITION OF TERMS 7](#_Toc534965048)

[SYNOPSIS 8](#_Toc534965049)

[1. INTRODUCTION 12](#_Toc534965050)

[1.1. Smoking and Health 12](#_Toc534965051)

[1.2. Next Generation Products 13](#_Toc534965052)

[1.2.1. Tobacco Heating Products 13](#_Toc534965053)

[1.2.1.1. THP1.1(RT) Tobacco Heating Product 14](#_Toc534965054)

[1.2.1.1.a. Product Use 14](#_Toc534965055)

[1.2.1.1.b. THP1.1(RT) Tobacco Heating Product Consumables 14](#_Toc534965056)

[1.2.2. Clinical Experience 15](#_Toc534965057)

[1.3. Rationale for the Study 15](#_Toc534965058)

[2. OBJECTIVES AND ENDPOINTS 16](#_Toc534965059)

[2.1. Primary Objective 16](#_Toc534965060)

[2.2. Secondary Objectives 16](#_Toc534965061)

[2.3. Exploratory Objectives 16](#_Toc534965062)

[2.4. Endpoints 17](#_Toc534965063)

[2.4.1. Primary Endpoints 17](#_Toc534965064)

[2.4.2. Secondary Endpoints 17](#_Toc534965065)

[2.4.3. Exploratory Endpoints 19](#_Toc534965066)

[3. INVESTIGATIONAL PLAN 19](#_Toc534965067)

[3.1. Overall Study Design and Plan 19](#_Toc534965068)

[3.1.1. Duration 22](#_Toc534965069)

[3.1.2. Study Arms 22](#_Toc534965070)

[3.1.3. Product Use 22](#_Toc534965071)

[3.2. Justification of Study Design 23](#_Toc534965072)

[3.3. Subject Withdrawal Criteria 23](#_Toc534965073)

[3.4. Smoking Advice and Support 25](#_Toc534965074)

[3.4.1. All Subjects 25](#_Toc534965075)

[3.4.2. Smoking Cessation Arm (Arm D) 25](#_Toc534965076)

[3.5. Management of Subject Safety 25](#_Toc534965077)

[4. INVESTIGATIONAL PRODUCT 26](#_Toc534965078)

[4.1. Identity of Investigational Products 26](#_Toc534965079)

[4.2. Accountability 26](#_Toc534965080)

[4.3. Blinding 26](#_Toc534965081)

[4.4. Randomisation 27](#_Toc534965082)

[4.5. Compliance 27](#_Toc534965083)

[5. Selection of Study Population 27](#_Toc534965084)

[5.1. Selection of Study Population 27](#_Toc534965085)

[5.2. Determination of Sample Size 28](#_Toc534965086)

[5.3. Inclusion Criteria 28](#_Toc534965087)

[5.4. Exclusion Criteria 30](#_Toc534965088)

[6. STUDY ACTIVITIES 32](#_Toc534965089)

[6.1. Screening 32](#_Toc534965090)

[6.1.1. Screening Visit 32](#_Toc534965091)

[6.1.2. Re-screening 33](#_Toc534965092)

[6.2. Visit 1 33](#_Toc534965093)

[6.2.1. Continue-to-Smoke/Next Generation Product Population Only 34](#_Toc534965094)

[6.2.2. Intend-to-quit Population 34](#_Toc534965095)

[6.2.3. Never-smoked Population 35](#_Toc534965096)

[6.3. Visit 2 to Visit 13 35](#_Toc534965097)

[6.4. Follow-up Contact 36](#_Toc534965098)

[6.4.1. Visits 1 to 12 36](#_Toc534965099)

[6.4.2. Visit 13 36](#_Toc534965100)

[6.5. Study Termination 37](#_Toc534965101)

[7. STUDY PROCEDURES 37](#_Toc534965102)

[7.1. Specific Restrictions/Requirements 37](#_Toc534965103)

[7.1.1. Concomitant Medication 37](#_Toc534965104)

[7.1.2. Smoking and Nicotine Use 38](#_Toc534965105)

[7.1.3. Diet 38](#_Toc534965106)

[7.1.4. Alcohol 38](#_Toc534965107)

[7.1.5. Exercise 39](#_Toc534965108)

[7.1.6. Blood Donation 39](#_Toc534965109)

[7.1.7. Contraception 39](#_Toc534965110)

[7.2. Clinical Assessments 40](#_Toc534965111)

[7.2.1. Demographic Data 40](#_Toc534965112)

[7.2.2. Smoking History and Willingness to Quit Smoking 40](#_Toc534965113)

[7.2.3. Medical History and Concomitant Diseases 40](#_Toc534965114)

[7.2.4. Vital Signs 40](#_Toc534965115)

[7.2.5. Physical Examination 41](#_Toc534965116)

[7.2.6. Height, Body Weight, and BMI 41](#_Toc534965117)

[7.2.7. Electrocardiography 41](#_Toc534965118)

[7.2.8. Lung Function Tests 41](#_Toc534965119)

[7.2.9. Clinical Laboratory Evaluations 42](#_Toc534965120)

[7.2.10. Urine Drugs of Abuse Screen and Alcohol Breath Test 43](#_Toc534965121)

[7.2.11. Body Fat Analysis 44](#_Toc534965122)

[7.3. Sample Collection for Biomarker and Exploratory Endpoint Assessments 44](#_Toc534965123)

[7.3.1. Exhaled Carbon Monoxide and Nitric Oxide 44](#_Toc534965124)

[7.3.2. Urinary BoE and BoBE 44](#_Toc534965125)

[7.3.3. Blood Sampling for BoE, BoBE, and Exploratory Endpoints 44](#_Toc534965126)

[7.4. Sample Handling, Transport and Storage 44](#_Toc534965127)

[7.4.1. Urine Samples 45](#_Toc534965128)

[7.4.2. Blood Samples 45](#_Toc534965129)

[7.5. Other Study Assessments 46](#_Toc534965130)

[7.5.1. Physiological Assessments 46](#_Toc534965131)

[7.5.1.1. Body Weight and Waist Circumference 46](#_Toc534965132)

[7.5.1.2. Augmentation Index and Carotid/Femoral Pulse Wave Velocity 46](#_Toc534965133)

[7.5.1.3. 6-minute Walking Test 46](#_Toc534965134)

[7.5.1.4. Finger Plethysmography 46](#_Toc534965135)

[7.5.2. Questionnaires 47](#_Toc534965136)

[7.5.2.1. Tobacco Use History (All Subjects) 47](#_Toc534965137)

[7.5.2.2. Fagerström Test for Cigarette Dependence (Arms A, B, and D Only) 47](#_Toc534965138)

[7.5.2.3. Product Satisfaction (Arms A and B Only) 47](#_Toc534965139)

[7.5.2.4. Smoking Cessation Quality of Life (Arms B and D only) 47](#_Toc534965140)

[7.5.2.5. Cough and Shortness of Breath (All Subjects) 47](#_Toc534965141)

[7.5.2.6. Product Use Count (Arms A, B, and D Only) 48](#_Toc534965142)

[8. ADVERSE EVENTS 48](#_Toc534965143)

[8.1. Definitions 48](#_Toc534965144)

[8.1.1. Adverse Events 48](#_Toc534965145)

[8.1.2. Serious Adverse Events 48](#_Toc534965146)

[8.2. Assessment of Adverse Events 49](#_Toc534965147)

[8.3. Intensity of Adverse Events 49](#_Toc534965148)

[8.4. SAE Reporting 50](#_Toc534965149)

[8.4.1. Abnormal Results of Laboratory Tests 50](#_Toc534965150)

[8.4.2. Abnormal Results of Other Tests and Investigations 51](#_Toc534965151)

[8.5. Reporting and Follow-up of Pregnancies 51](#_Toc534965152)

[8.6. Adverse Events Leading to Withdrawal 51](#_Toc534965153)

[8.7. Investigational Product Misuse 52](#_Toc534965154)

[8.8. Investigational Device Malfunctions 52](#_Toc534965155)

[9. DATA ANALYSIS 52](#_Toc534965156)

[9.1. General Considerations 52](#_Toc534965157)

[9.2. Analysis Populations 53](#_Toc534965158)

[9.3. Statistical Analysis of Primary and Secondary Objectives 53](#_Toc534965159)

[9.4. Safety Data Summary and Analysis 54](#_Toc534965160)

[10. REPORTS AND PUBLICATIONS 54](#_Toc534965161)

[10.1. Reports to the Ethics Committee 54](#_Toc534965162)

[10.2. Clinical Study Report 54](#_Toc534965163)

[10.3. Publication and Disclosure Policy 55](#_Toc534965164)

[11. REGULATORY CONSIDERATIONS 55](#_Toc534965165)

[11.1. Clinical Trial Authorisation 55](#_Toc534965166)

[11.2. Visits by Regulatory Authorities 55](#_Toc534965167)

[12. ETHICAL CONSIDERATIONS 55](#_Toc534965168)

[12.1. Ethics Committee Approval 55](#_Toc534965169)

[12.2. Addressing Ethical Issues and the Ethical Conduct of the Study 56](#_Toc534965170)

[12.3. Subject Information and Informed Consent 56](#_Toc534965171)

[12.4. Declaration of Helsinki 57](#_Toc534965172)

[12.5. Good Clinical Practice (GCP) 57](#_Toc534965173)

[12.6. Adherence to the Protocol 57](#_Toc534965174)

[12.7. Protocol Amendments 57](#_Toc534965175)

[13. DATA MANAGEMENT METHODS 57](#_Toc534965176)

[13.1. Data Quality Assurance 57](#_Toc534965177)

[13.2. Case Report Form 58](#_Toc534965178)

[13.3. Monitoring 58](#_Toc534965179)

[13.4. Data Storage and Archiving 58](#_Toc534965180)

[14. REFERENCES 59](#_Toc534965181)

[15. APPENDICES 61](#_Toc534965182)

[Appendix 15.1: Schedule of Assessments 62](#_Toc534965183)

**LIST OF FIGURES**

[Figure 1: Study Schematic 34](#_Toc503183717)

LIST OF ABBREVIATIONS

| AE | adverse event |
| --- | --- |
| AIx | Augmentation Index |
| BAT | British American Tobacco (Investments) Ltd. |
| BMI | body mass index |
| BoBE | biomarker of biological effect |
| BoE | biomarker of exposure |
| CO | carbon monoxide |
| CPD | cigarettes per day |
| CRU | Clinical Research Unit |
| CTA | Clinical Trial Authorisation |
| CYP | cytochrome P450 |
| EC | ethics committee |
| ECG | electrocardiogram |
| eCRF | electronic Case Report Form |
| FDA | Food and Drug Administration |
| FEV_1_ | forced expiratory volume in 1 second |
| FTCD | Fagerström test for cigarette dependence |
| FVC | forced vital capacity |
| IB | Investigator’s Brochure |
| ICF | Informed Consent Form |
| ICH | International Council for Harmonisation |
| ITT | intent-to-treat population |
| LED | light-emitting diode |
| MHRA | Medicines and Healthcare products Regulatory Agency |
| MRTP | modified risk tobacco product |
| NGP | Next Generation Product |
| NO | nitric oxide |
| NRT | nicotine replacement therapy |
| OTC | over-the-counter |
| PP | per-protocol population |
| QTcF | QT interval corrected for heart rate using Fridericia’s method |
| SAE | serious adverse event |
| THP | tobacco heating product |
| VAS | visual analog scale |
| WBC | white blood cell |
| WHO | World Health Organisation |

DEFINITION OF TERMS

| Baseline assessments | Assessments that occur before the product test on Day 1 |
| --- | --- |
| Concomitant medication | Concomitant medication refers to all medication taken during the study conduct period from the Informed Consent Form (ICF) signature onwards. Medications started prior to signing of the ICF but which the subject continued to take during the study, were considered to be concomitant medications. |
| Conventional cigarette | Conventional cigarette refers to commercially available manufactured cigarettes, and excludes other tobacco products including hand-rolled cigarettes, cigars, pipes and bidis. |
| Enrolled not randomised | Any subject in Arms A or B who is enrolled onto the study on Day 1 but who withdraws or is withdrawn prior to randomisation. |
| Exposure period | From start of randomised product use (Arms A or B) on Day 1 until Check‑out from the clinic on Day 360. |
| Follow-up | Follow-up Visit will occur within 28 days following Check-out on Day 360. |
| Randomisation | Assignment of subjects in Arms A or B to a specific product use arm on Day 1. |
| Screening | Screening is defined as the 28 day period prior to Visit 1, during which subjects will undergo a Screening Visit. |
| Screening failure | Any subject who does not meet the entry criteria prior to enrolment will be considered a screening failure. |
| Sponsor | ‘Sponsor’ refers to British American Tobacco (Investments) Ltd. R&D Centre, Regents Park Road, Southampton, Hampshire, UK. SO15 8TL, who is funding the study. |
| Subject | ‘Subject’ refers to an individual who participates in the clinical study. |
| Tobacco heating product | A tobacco heating product (THP) is an electronic device which heats tobacco, typically to temperatures lower than 350°C, rather than combusting it. |

SYNOPSIS

| Title of Study: | A randomised, controlled study to evaluate the effects of switching from cigarette smoking to using a Tobacco Heating Product on health effect indicators in healthy subjects |
| --- | --- |
| Objectives: | **Primary Objective:**   - To quantitatively assess differences in primary study endpoints at 90, 180, and 360 days between subjects who continue to smoke conventional cigarettes and subjects who switch to a tobacco heating product (THP)   **Secondary Objectives:**   - To quantitatively assess differences in secondary study endpoints between subjects who continue to smoke conventional cigarettes and subjects who switch to a THP. - To assess differences in all study endpoints between subjects who switch to a THP and subjects in the assisted smoking cessation arm. - To assess the differences in all study endpoints between subjects who switch to a THP or undertake assisted smoking cessation, and subjects who have never smoked. - To monitor the safety profile of subjects using THPs and combustible cigarettes, and subjects in the smoking cessation and never-smoked arms.   **Exploratory Objectives:**   - To quantitatively assess the time required to observe changes in selected primary and secondary endpoints following a switch from conventional cigarettes to a THP or assisted cessation. - To quantitatively assess differences in the exploratory endpoints between subjects who continue to smoke conventional cigarettes, subjects who switch to a THP, subjects in the cessation arm, and subjects who have never smoked (to be reported separately). - To investigate the profile of selected primary and secondary endpoints over the course of the study for each study arm.   **Primary Endpoints:**  *24-hour Urine (ambulatory collection):*   - Total 4-(methylnitrosamino)-1-(3-pyridyl)-1-butanol (Total NNAL) - 8-epi-prostaglandin F_2α_ Type III (8-Epi-PGF_2α_ Type III)   *Physiological Measures:*   - Augmentation Index (AIx)   **Secondary Endpoints:**  *24-hour Urine (ambulatory collection):*   - Total nicotine equivalents (nicotine, cotinine, 3-hydroxycotinine and their glucuronide conjugates) (TNeq) - Total N-nitrosonornicotine (Total NNN) - 3-hydroxypropylmercapturic acid (3-HPMA) - 3-hydroxy-1-methylpropylmercapturic acid (HMPMA) - S-phenylmercapturic acid (S-PMA) - [Monohydroxybutenyl-mercapturic acid](http://onlinelibrary.wiley.com/doi/10.1002/3527600418.bi10699e0011/pdf) (MHBMA) - 2-cyanoethylmercapturic acid (CEMA) - 1-hydroxypyrene (1-OHP) - 2-hydroxyethylmercapturic acid (HEMA) - 11-dehydrothromboxane B2 (11-dTX B2) - 4-hydroxy-nonenal + metabolites (4-HNE) - 4-aminobiphenyl (4-ABP) - 2-aminonapththalene (2-AN) - Ortho-toluidine (o-Tol) - Creatinine   *Blood:*   - White blood cell count (WBC count) - Monocyte chemotactic protein 1/C-C motif chemokine ligand 2 (MCP‑1/CCL2) - Soluble intercellular adhesion molecule-1 (s-ICAM1) - Fibrinogen (Fib) - High-sensitivity C-reactive protein (hsCRP) - Homocysteine (HMCys) - Glucose (Gluc) - Plasminogen activator inhibitor-1 (PAI-1) - Tissue plasminogen activator (tPA) - E-Selectin (SELE) - Endothelin-1 (ET-1) - 3- nitrotyrosine (3-NTyr) - Serum lipids (high-density lipoprotein [HDL], low-density lipoprotein [LDL], total cholesterol [CholTotal], triglycerides [Trigly])   *Exhaled Breath:*   - Nitric oxide (NO) - Carbon monoxide (CO)   *Physiological Measures:*   - Body weight/waist circumference - Carotid/femoral pulse wave velocity - 6-minute walking test - Finger plethysmography   *Questionnaires:*   - Fagerström Test for Cigarette Dependence (FTCD) - Product Satisfaction - Smoking Cessation Quality of Life - Self-reported product usage (eDiary/paper diary) - Cough and Shortness of Breath VAS   *Safety:*   - Physical examination - Vital signs - Electrocardiogram (ECG) - Clinical laboratory evaluations - Lung function tests (spirometry)   - Peak flow   - Forced vital capacity (FVC)   - Forced expiratory flow (FEF) 25-75%   - Forced expiratory volume in 1 second (FEV_1_) - Adverse events (AE)/serious adverse events (SAE) recording   *Compliance Endpoints:*  *Blood:*   - N-(2-cyanoethyl)valine (HB [haemoglobin] adduct; CEVal)   **Exploratory Endpoints:**  Sample collections for the following exploratory analyses will also be taken (to be reported separately, with the exception of body fat analysis):   - Body fat analysis - Dihydrobiopterin/tetrahydrobiopterin (BH2/BH4) ratio in plasma - Transcriptomics (nasal epithelial cells) - Transcriptomics (white blood cells) - Serum nuclear magnetic resonance (NMR) lipoprotein - Serum metabolomics |
| Study Design: | This is a multi-centre, controlled study utilising a randomised switching design for cigarette smoking and THP use. Three separate populations of subjects will be recruited and randomised/enrolled as follows:   \| **Population** \| **Study arm and randomised product** \| \| --- \| --- \| \| Continue-to-smoke/THP population \| Arm A – conventional cigarettes  Arm B – THP1.1(RT) \| \| Intend-to-quit population \| Arm D – assisted smoking cessation \| \| Never-smoked population \| Arm E – never-smoked \|   This study will be ambulatory, with subjects in Arms A, B, and D attending a total of 13 non-residential clinic visits (approximately 1 every 30 days) plus a Screening Visit and a Follow-up Visit over a period of 12 months. Subjects in Arm E will attend a total of 4 non‑residential clinic visits (Day 1 and approximately 3, 6 and 12 months post-enrolment) plus a Screening Visit and a Follow-up Visit. |
| Number of Subjects: | It is planned for 495 subjects to be enrolled into Arms A, B, D, and E, with the aim of 180 subjects completing the study. It should be noted that 34 subjects had been randomized into Arm C prior to issue of Amendment 7. |
| Main Criteria for Inclusion/ Exclusion: | The main inclusion criteria are:  All populations:   - Subjects will be healthy males or females, within the ages of 23 to 55 years, inclusive. - Subjects will have a body mass index (BMI) between 17.6 and 32.0 kg/m^2^, inclusive, and a body weight exceeding 50 kg (males) or 40 kg (females).   Arms A, B, and D:   - Subjects will be regular smokers of commercially manufactured filter cigarettes and/or roll your own cigarettes. - Subjects will have smoked for at least 5 consecutive years prior to Screening, and will typically smoke at least 10 and a maximum of 30 cigarettes per day.   The main exclusion criteria are:  All subjects   - Subjects who are pregnant. - Subjects who have an acute illness (e.g. upper respiratory tract infection) requiring treatment within 4 weeks prior to Visit 1 (subjects who had viral infections that resolved ≥2 weeks prior to Visit 1 will be admissible to this study). - Subjects who have used prescription or over-the-counter (OTC) bronchodilator medication (e.g. inhaled or oral β-adrenergic agonists) to treat a chronic condition within the 12 months prior to Visit 1. - Subjects who have received any medications or substances (other than tobacco) which interfere with the cyclooxygenase pathway (e.g. anti-inflammatory drugs including aspirin and ibuprofen) within 14 days prior to Visit 1, or which are known to be strong inducers or inhibitors of cytochrome P450 (CYP) enzymes within 14 days or 5 half‑lives of the drug (whichever is longer) prior to Visit 1.   Arms A, B, and D:   - Subjects who regularly use any nicotine or tobacco products other than commercially manufactured filter cigarettes and/or roll your own cigarettes within 14 days of Screening.   Arms A and B:   - Subjects who are self-reported non-inhalers (smokers who draw smoke from the cigarette into the mouth and throat but who do not inhale). - Subjects who, prior to enrolment, are planning to quit smoking in the next 12 months. All subjects will be informed that they are free to quit smoking and withdraw from the study at any time. |
| Test Products: | Details of the study product are presented in the following table:   \| **Study Product** \| **Product Code** \| **Category** \| **Product & Manufacturer** \| **Nicotine Yield/Content** \| \| --- \| --- \| --- \| --- \| --- \| \| **1** \| THP1.1(RT) \| THP \| Glo device with neostik, British American Tobacco \| 0.68 mg/ neostik^a^ \| \| ^a^ nicotine yield under modified Health Canada Intense machine smoking regime; 55 mL puff volume; 30 second puff interval; 2 second puff duration; 8 puffs; no vent blocking. \| \| \| \| \| |
| Duration of Study: | **Screening Duration:** 28 days  **Study Duration:** 360 days  **Follow-up Duration:** ≤28 days |
| Criteria for Evaluation: | Biomarkers of exposure (BoE), biomarkers of biological effect (BoBE), physiological endpoints, questionnaire assessments, and exploratory endpoints will be assessed in this study. Blood samples, exhaled breath, and 24-hour urine samples will be collected for assessment of BoE, BoBE, and exploratory endpoints.  The safety evaluations for this study will include AEs, vital signs, clinical laboratory evaluations, physical examinations, ECG, and lung function tests. |
| Statistical Methods: | In general, continuous variables will be presented by means of descriptive statistics (n, mean, standard deviation, median, minimum and maximum) and categorical variables will be displayed by means of frequency tables and, where appropriate, shift tables. All summaries will be presented by study arm, sex, age, and timepoint. All data collected for the study will be presented as data listings for each subject.  The primary objective will be examined by computing levels of biomarkers at each timepoint, i.e. baseline, 90, 180, and 360 days. These data will be compared between the THP (Arm B) and the main control arm (Arms A) using specific contrast tests from statistical models adjusted for baseline measurements. If any endpoint were to be significant at Day 90 or 180, it will not be statistically assessed at Day 180 and/or 360, as appropriate, and its assigned alpha level will be equally distributed between the remaining primary endpoints. Similarly, biomarker measures in the secondary objectives will be examined by computing levels of biomarkers at each timepoint. These data will be compared between THP and the main control arm using specific contrast tests from statistical models adjusted for baseline measurements. |

1. INTRODUCTION
   1. Smoking and Health

Cigarette smoking is a well-known cause of human disease. The health risks associated with cigarette smoking are correlated with duration of smoking and degree of daily cigarette consumption, and cessation reduces an individual’s relative risks of tobacco‑related
disease.^1–4^ Thus, tobacco-related health risks are assumed to be due to repeated and sustained exposure to a range of smoke toxicants.^5,6^

Reducing the negative health burden of tobacco use is a clear public health priority and has led to a series of regulatory and educational initiatives to persuade people not to smoke.^1,4^ Despite these efforts, smoking rates in adult populations worldwide remain relatively high,^1,4^ and the World Health Organisation (WHO) has forecast that there will be around 1.5 billion tobacco smokers worldwide in 2050.^5^

It is therefore important to complement existing initiatives with strategies to attempt to reduce or prevent harm in those who will otherwise continue to smoke. Tobacco harm reduction, the substitution of potentially reduced-risk products for cigarette smoking, is a strategy that if widely adopted could offer substantial public health gains.^7^ For many years, tobacco researchers and policy experts have embraced the idea that alternative sources of nicotine to smoking that could provide similar rewarding effects as a cigarette might entice smokers away from cigarette smoking. This could lead to them either quitting smoking or switching to long‑term nicotine use without incurring the harm anticipated from exposure to cigarette smoke.

Smoke from conventional cigarettes is a complex and dynamic mixture of more than 5,600 identified chemical constituents,^8^ some of which have been identified as potential contributors to the harmful effects of cigarette smoke.^6^

Nicotine, a chemical also found naturally in tobacco leaf and which transfers into cigarette smoke, is primarily responsible for the addictive properties of cigarette smoking, but is not considered to contribute to smoking-related diseases.^3,9^ Nicotine in the tobacco smoke rapidly activates neuronal nicotinic receptors in the brain to elicit its effect on mood and relaxation, which is largely responsible for the pleasurable effects experienced by the smoker.^10^

Pharmaceutical nicotine products (i.e. nicotine replacement therapies [NRTs]) replace the nicotine absorbed from cigarettes and are thought to assist subjects in stopping smoking by reducing cravings, symptoms of withdrawal, and mood changes. Examples of NRTs include nicotine patches, gums, and more recently, sprays and other inhaled vehicles. In general, the delivery of nicotine from NRT products is relatively slow and the pharmacokinetic profile does not fully resemble that of cigarettes.^11,12^ The time to maximum concentration (t_max_) tends to be longer, and the maximum concentration (C_max_) is not characterised by the sharp peak, but by a lower and flatter peak. Smokers do not therefore achieve the same nicotine experience and satisfaction with NRT products that they do when smoking cigarettes. This may be a contributor to the relatively poor efficacy of NRT products as aids to smoking cessation.

- 1. Next Generation Products

Next Generation Products (NGPs) are nicotine-delivering devices that can be broadly categorised as either electronic inhalable vapour products (e-cigarettes) or tobacco heating products (THPs). This study will exclusively investigate a THP.

- - 1. Tobacco Heating Products

Tobacco heating products are electronic devices that heat tobacco, typically to temperatures lower than 350°C, rather than combusting it. Due to this lack of combustion, significantly fewer chemical toxicants are formed but nicotine is still released into the inhaled aerosol. THP sticks are similar in appearance to a cigarette, including a filter section at the mouth end of the tobacco stick. Less is known concerning the properties of THPs compared to e‑cigarettes; however, in-house assessments of the chemical toxicants found in the vapour from a THP when “smoked” on a smoking machine revealed significant reductions in the levels of many chemical toxicants when compared to those found in conventional cigarette smoke.^13^ The vapour has also been found to contain significant levels of nicotine.

There are few data showing human health effects of THPs, and certainly this is the case when compared to the broad literature on the health effects of cigarette smoking. The studies performed to date examining the effects of THP use in humans have predominantly measured biomarkers of exposure (BoE) to chemical toxicants found in regular cigarette smoke, in subjects who have switched from smoking to using an electrically heated THP. In these studies, large and significant reductions in exposure to a wide range of smoke chemicals have been reported. These effects were rapidly attained and sustained for a period of at least one month of continued use.^13–15^ These studies have been performed in several countries including Japan, South Korea, Poland and the UK.

Two further studies have reported similar reductions in exposure biomarkers in Japanese and Polish smokers when they switched to using a THP with a carbon heating source.^16,17^ Adverse events (AE) related to THP use during these studies were infrequent and included those commonly associated with cigarette smoking (e.g. dizziness and nausea) or with participation in confined clinical studies (e.g. mild headache).

One study assessed changes in biomarkers of health associated with cardiovascular disease (high‑density lipoprotein cholesterol, red blood cell count, haemoglobin and haematocrit levels), and demonstrated favourable changes in smokers who had switched from conventional cigarettes to a THP over a 1-month period.^15^ The most commonly reported AEs in this study that were considered related to the THP were dry mouth, dry throat, cough and diarrhoea, and occurred in 0.4% to 1.3% of 237 subjects. One serious adverse event (SAE) of deep vein thrombosis was also reported. This event was considered by the Investigator to be possibly related to the THP as cigarette smoking is a known risk factor for this condition.

It is anticipated that due to the much-altered chemical profile of a THP aerosol and the demonstrated reductions in chemical exposure associated with their use, the long-term health effects of THPs would potentially be much reduced compared to cigarette smoking. This potential for a change in relative risk still needs to be quantified, as does the absolute risk of THP use.

- - - 1. THP1.1(RT) Tobacco Heating Product

The THP1.1(RT) THP is a novel THP that has been designed and manufactured by the BAT group of companies. The THP1.1(RT) THP is made up of 2 components; a cylindrical shaped tobacco consumable and an electronic heating device into which the consumable is inserted before use. The heating device is comprised of a rechargeable battery, an electrical element which heats the consumable, and electronic hardware which controls the warming up, heating temperature and heating period of the device.

- - - - 1. Product Use

To use the THP1.1(RT) THP, the user inserts a consumable into a port on the top of the THP device, leaving the filter end protruding from the end of the device (as illustrated below). The port is accessed by moving a sliding cover which reveals the circular port. Once the consumable is inserted, the user pushes and holds an activation button on the THP for 3 seconds. This will cause the heating process to start and the THP will vibrate briefly to indicate that this has begun; one of a series of 4 LED lights on the front of the device and surrounding the activation button will also illuminate to further indicate that heating has begun. During the heating process, the other lights will also illuminate and once heating is complete all lights will be lit and the device will again vibrate to indicate that heating is complete and that the user can begin puffing. The device is designed so that the heating-up time will take 40 seconds. Once heating is complete, the device will only continue heating for 210 seconds. After this time the heater will shut down.

**The THP1.1(RT) Tobacco Heating Product:**


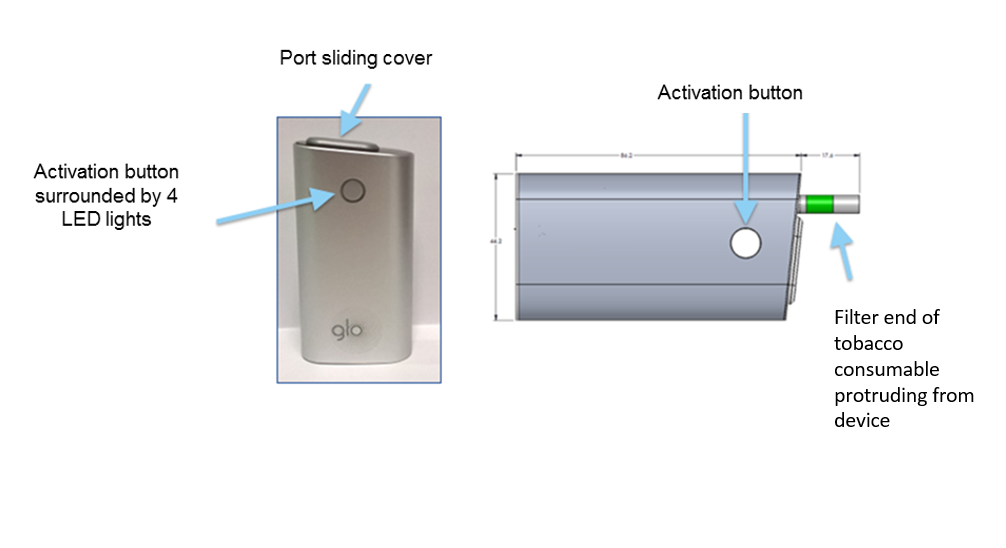


- - - - 1. THP1.1(RT) Tobacco Heating Product Consumables

The THP1.1(RT) THP consumables (neostiks) are cylinders containing blended tobacco sheet and a filter, wrapped in a paper outer. The consumables are 83 mm in length and 5.4 mm in diameter. The tobacco section is 42 mm in length and contains 210 mg of tobacco. The total weight of a consumable is 540 mg.

The consumables are designed to only be used with the THP1.1(RT) THP device and should not be misused by either being lit in a similar way to a combustible cigarette or used in a different tobacco heating device. Once a consumable has been used, it can be discarded in a regular waste bin.

- - 1. Clinical Experience

The Sponsor has assessed a similar product in 1 completed study in Japan,^13^ and a currently ongoing study in the United Kingdom. Both studies assessed BoE and nicotine pharmacokinetics in a clinical confinement setting. The investigational product is commercially available in Japan, Italy, Canada, Romania, South Korea, Russia, and Switzerland, but is not commercially available in the UK.

- 1. Rationale for the Study

Premarketing authorisations for novel tobacco products are required by some regulators, and applications in support of such authorisations may be required to include data from clinical studies to identify any potential reduction in individuals’ risk, relative to the risk of continued smoking.

The purpose of this study is to:

- Test the hypothesis that reduction in exposure to toxicants will cause changes in health effect indicators when smokers switch to using a THP compared with smokers who continue to smoke, and that these changes are directionally similar to changes seen in smokers who cease smoking (assisted with NRT), over a period of 12 months in an ambulatory setting.
- Test the hypothesis that reductions in toxicant levels in THP emissions translate to sustained reductions in human exposure to cigarette smoke toxicants, as assessed by measuring BoE in an ambulatory setting.

1. OBJECTIVES AND ENDPOINTS
   1. Primary Objective

- To quantitatively assess differences in primary study endpoints at 90, 180, and 360 days between subjects who continue to smoke conventional cigarettes and subjects who switch to a THP.
  1. Secondary Objectives
- To quantitatively assess differences in secondary study endpoints between subjects who continue to smoke conventional cigarettes and subjects who switch to a THP.
- To assess differences in all study endpoints between subjects who switch to a THP and subjects in the assisted smoking cessation arm.
- To assess the differences in all study endpoints between subjects who switch to a THP or undertake assisted smoking cessation, and subjects who have never smoked.
- To monitor the safety profile of subjects using THPs and combustible cigarettes, and subjects in the smoking cessation and never-smoked arms.
  1. Exploratory Objectives
- To quantitatively assess the time required to observe changes in selected primary and secondary endpoints following a switch from conventional cigarettes to a THP or assisted cessation.
- To quantitatively assess differences in the exploratory endpoints between subjects who continue to smoke conventional cigarettes, subjects who switch to a THP, subjects in the cessation arm, and subjects who have never smoked (to be reported separately).
- To investigate the profile of selected primary and secondary endpoints over the course of the study for each study arm.
  1. Endpoints
     1. Primary Endpoints

Primary endpoint biomarkers to be assessed are presented in the following table:

| **Biomarker** | **Abbreviation** | **Matrix** | **Indication** |
| --- | --- | --- | --- |
| Total 4-(methylnitrosamino)-1-(3-pyridyl)-1-butanol | Total NNAL | Urine  (24-hour) * | Metabolite of the smoke toxicant 4-(methylnitrosamino)-1-(3-pyridyl)-1-butanone (NNK) |
| 8-epi-prostaglandin F_2α_ Type III | 8-Epi-PGF_2α_ Type III | Urine  (24-hour) * | Marker of oxidative stress |
| Augmentation Index | AIx | Physiological measure | Marker of arterial stiffness and hence an indicator for cardiovascular risk |

* ambulatory collection.

- - 1. Secondary Endpoints

Secondary endpoint biomarkers to be assessed are presented in the following table:

| **Biomarker of Exposure** | **Abbreviation** | **Associated Toxicant** | **Matrix** |
| --- | --- | --- | --- |
| Carbon monoxide†† | CO | Carbon monoxide | Exhaled breath |
| Total nicotine equivalents (nicotine, cotinine, 3-hydroxycotinine and their glucuronide conjugates) | TNeq | Nicotine | Urine  (24-hour) * |
| Total N-nitrosonornicotine | Total NNN | NNN | Urine  (24-hour) * |
| 3-hydroxypropylmercapturic acid | 3-HPMA | Acrolein | Urine  (24-hour) * |
| 3-hydroxy-1-methylpropylmercapturic acid | HMPMA | Crotonaldehyde | Urine  (24-hour) * |
| S-phenylmercapturic acid | S-PMA | Benzene | Urine  (24-hour) * |
| [Monohydroxybutenyl-mercapturic acid](http://onlinelibrary.wiley.com/doi/10.1002/3527600418.bi10699e0011/pdf) | MHBMA | 1,3-butadiene | Urine  (24-hour) * |
| 2-cyanoethylmercapturic acid | CEMA | Acrylonitrile | Urine  (24-hour) * |
| 1-hydroxypyrene | 1-OHP | Pyrene | Urine  (24-hour) * |
| 2-hydroxyethylmercapturic acid | HEMA | Ethylene oxide | Urine  (24-hour) * |
| 4-aminobiphenyl | 4-ABP | 4-aminobiphenyl | Urine (24‑hour) * |
| 2-aminonaphthalene | 2-AN | 2-aminonaphthalene | Urine (24‑hour) * |
| Ortho-toluidine | o-Tol | Ortho-toluidine | Urine (24‑hour) * |

| **Biomarker of Compliance** | | **Abbreviation** | **Indication** | **Matrix** |
| --- | --- | --- | --- | --- |
| N-(2-cyanoethyl)valine (haemoglobin adduct) † | | CEVal | Acrylonitrile | Whole blood |
| **Biomarker of Biological Effect** | | **Abbreviation** | **Indication** | **Matrix** |
| Nitric oxide | | NO | Bronchodilation/vascular tone | Exhaled breath |
| 11-dehydrothromboxane B2 | | 11-dTx B2 | Platelet activation/coagulation | Urine  (24-hour) * |
| 4-hydroxy-nonenal + metabolites | | 4-HNE | Oxidative stress | Urine  (24-hour) * |
| White blood cell count | | WBC count | Inflammation | Whole blood |
| Monocyte chemotactic protein 1/ C-C motif chemokine ligand 2 | | MCP-1/CCL2 | Inflammation/chemokine | Plasma |
| Soluble intercellular adhesion molecule-1 | | s-ICAM1 | Endothelial dysfunction | Plasma |
| Fibrinogen | | Fib | Coagulation | Plasma |
| High-sensitivity C-reactive protein | | hsCRP | Inflammation | Plasma |
| Homocysteine | | HMCys | Oxidative stress | Plasma |
| Glucose | | Gluc | Metabolic status | Plasma |
| Plasminogen activator inhibitor-1 | | PAI-1 | Coagulation | Plasma |
| Tissue plasminogen activator | | tPA | Coagulation | Serum |
| E-selectin | | SELE | Endothelial dysfunction | Plasma |
| Endothelin-1 | | ET-1 | Vascular tone/endothelial dysfunction | Serum |
| 3-nitrotyrosine | | 3-NTyr | Nitrosative stress/vascular tone | Plasma |
| Serum lipids (high-density lipoprotein [HDL], low-density lipoprotein [LDL], total cholesterol, triglycerides) | | HDL/LDL/ CholTotal/Trigly | Metabolic status | Serum |
| **Other Measures** | | | | |
| Creatinine (urine; 24-hour) † | | | | |
| **Physiological Measures** | | | | |
| Body weight/waist circumference | | | | |
| Carotid/femoral pulse wave velocity | | | | |
| 6-minute walking test | | | | |
| Finger plethysmography | | | | |
| **Questionnaires** | | | | |
| Fagerström Test for Cigarette Dependence (FTCD) † | | | | |
| Product Satisfaction † | | | | |
| Smoking Cessation Quality of Life | | | | |
| Self-reported product use (eDiary/paper diary) † | | | | |
| Cough and Shortness of Breath VAS | | | | |
| **Safety** | | | | |
| Physical examination † | | | | |
| Vital signs † | | | | |
| Electrocardiogram (ECG) † | | | | |
| Clinical laboratory evaluations † | | | | |
| Lung function test (spirometry) | Peak flow | | | |
|  | Forced vital capacity (FVC) | | | |
|  | Forced expiratory flow (FEF) 25-75% | | | |
|  | Forced expiratory volume in 1 second (FEV_1_) | | | |
| Adverse events (AE)/SAE recording † | | | | |

* Ambulatory collection.

† Blood pressure will be assessed using the statistical analysis as described in Section 9.3. Other endpoints may be analysed using a statistical method that will be described in the Statistical Analysis Plan.

†† The assessments on Days 120 and 150 will be used in the Day 90 interim analysis, the assessments on Days 210 and 240 will be used in the Day 180 interim analysis, and the assessments on Days 300 and 330 will be used in the final analysis on Day 360.

- - 1. Exploratory Endpoints

Body fat will be measured and reported.

Sample collections for the following exploratory analyses will also be taken and will be reported separately:

- Dihydrobiopterin/tetrahydrobiopterin (BH2/BH4) ratio in plasma
- Transcriptomics (nasal epithelial cells)
- Transcriptomics (WBC)
- Serum nuclear magnetic resonance (NMR) lipoprotein
- Serum metabolomics

1. INVESTIGATIONAL PLAN
   1. Overall Study Design and Plan

This is a multi-centre, controlled study utilising a randomised switching design for cigarette smoking and NGP use. Three separate populations will be recruited:

- Continue-to-smoke/THP population (n = 265)
- Intend-to-quit population (n = 190)
- Never-smoked population (n = 40)

Subjects in the continue-to-smoke/THP population will be randomised to 1 of the following study arms:

- Arm A – continue to smoke commercially manufactured filter cigarettes and/or roll your own cigarettes (n = 65)
- Arm B – switch to THP1.1(RT) THP (n = 200)

The aim is for 65 subjects (Arm A) and 200 subjects (Arm B) to be randomised, with the intention for a minimum of 50 subjects to complete per arm.

Subjects in the intend-to-quit population will be assigned to the assisted smoking cessation arm (Arm D). The aim is to recruit 190 healthy adult male and female regular smokers of 10 to 30 non-mentholated commercially manufactured filter cigarettes and/or roll your own cigarettes per day, and who are intending to cease all non-medicinal nicotine use. This group will be supported with NRT and/or varenicline as appropriate. The intended minimum number to complete is 50 subjects.

Subject in the never-smoked population will be assigned to Arm E. The aim is to recruit 40 subjects who have never smoked (<100 cigarettes in their lifetime and none in the 30 days prior to Screening), with the intention for a minimum of 30 subjects to complete.

A schematic of the study design is presented in Figure 1.

Figure 1: Study Schematic

*Subjects in Arm E will only attend Screening, Visit 1 (Day 1), Visit 4 (Day 90 +/- 3 days), Visit 7 (Day 180 +/- 2 weeks), Visit 13 (Day 360 (+/- 2 weeks), and Follow-up.

- - 1. Duration

This will be an ambulatory study, with subjects in Arms A, B, and D attending clinic visits at Screening, Day 1, Days 30, 60, and 90 (+/-3 days), Days 120, 150, 180, 210, 240, 270, 300, 330, and 360 (+/- 2 weeks), and at Follow-up (within 28 days following last visit). Subjects in Arm E will attend clinic visits at Screening, Day 1, Day 90 (+/-3 days), Days 180 and 360 (+/- 2 weeks), and at Follow-up (within 28 days following last visit).

Subjects will be randomised (Arms A and B) or enrolled (Arms D and E) on Day 1. Subjects will undergo screening within 28 days prior to Day 1, and will return for a Follow‑up Visit within 28 days of Day 360.

- - 1. Study Arms

Subjects will be randomised (Arms A and B) or assigned (Arms D and E) to one of the study arms presented below.

| **Study Arm** | **Recruitment Population** | **Randomised Product Use** |
| --- | --- | --- |
| A | THP/continue-to-smoke population | Continue to smoke subject’s usual brand of commercially manufactured filter cigarettes and/or roll your own cigarettes |
| B |  | THP1 (THP1.1(RT)) |
| D | Intend-to-quit population | None |
| E | Never-smoked population | None |

- - 1. Product Use

In Arms A, B and D, the subject will be asked to continue smoking their usual brand of cigarette until randomisation (Arms A and B) or enrolment (Arm D) on Day 1.

Subjects in Arms A and B will use only their assigned product following randomisation until Check-out from the final non-residential clinic visit on Day 360. Subjects in Arm D will use only specific NRT/smoking cessation aids as described in Section 3.4.2 following enrolment until Check-out from the final non-residential clinic visit on Day 360. Subjects in Arm E will not use any products/NRT/smoke cigarettes during the study.

For Arms A and B, smoking/product use following randomisation will be *ad libitum*, subject to an average product use of up to 200% of their self-reported cigarettes per day at Screening. Subjects will be reminded of the risks associated with smoking prior to enrolment onto the study and that they are free to voluntarily quit smoking and/or withdraw from the study at any time (Section 3.4).

For subjects in Arm B:

- At Visit 1, subjects in Arm B will be provided with no more than 150% of their self‑reported cigarettes per day (CPD), with the provision to obtain the remaining 50% before Visit 2 by visiting the site.
- At Visits 2 to 12, an assessment of product usage should be undertaken, and the allocation of product should be 120% of the product used in the previous period, up to the limit of 200% (self-reported pre-study CPD). At these visits, subjects will return all empty, part-used, and unused packs of THP consumables.
- At Visit 13, subjects will return all empty, part-used, and unused packs of THP consumables, and the study device, chargers and other accessories supplied to them for this study.

Subjects randomised to Arm A will continue to smoke their usual brand of cigarettes at their own expense.

- 1. Justification of Study Design

Subjects in this study will be a minimum of 23 years of age. This is based on:

- The legal age to obtain tobacco products in the United Kingdom is 18 years
- Subjects will be required to have a smoking history of at least 5 years

To investigate the effects of smoking cessation over 12 months in smokers, a population of subjects who are intending to quit smoking will be recruited. It is anticipated that a higher proportion of these subjects will not complete the study, in comparison to recruiting subjects who are not intending to quit. Having an intend-to-quit population will also reassure subjects who do not want to quit smoking (the continue-to-smoke/THP population) that they will not be randomised to the cessation arm, thus aiding recruitment.

The BoE selected as endpoints in this study are based around the initial list of priority toxicants proposed by the WHO Study Group on Tobacco Product Regulation.^18^

The biomarkers of biological effect (BoBE) selected for evaluation are markers for bronchodilation/vascular tone, platelet activation, coagulation, oxidative stress, tissue remodelling, inflammation/chemokine, endothelial dysfunction, metabolic status, and nitrosative stress. Within the categories mentioned above, endpoints have been selected to support various events within the biological pathway of interest that they pertain to (e.g. finger plethysmography, endothelin-1, 3-nitrotyrosine, and NO are all related endpoints to support the mechanisms which control vascular tone and endothelial function).

- 1. Subject Withdrawal Criteria

Subjects may be withdrawn from the study prematurely for the following reasons:

1. If a subject experiences an intolerable AE, premature discontinuation will be at the discretion of either the Investigator or their appropriately qualified designee, or the subject, independent of the relationship of the AE to the study test product. The appropriate AE electronic Case Report Form (eCRF) page must be completed.
2. If a subject develops a non-fulfilment of inclusion/exclusion criteria or concurrent disease, which at the discretion of the Investigator (or their appropriately qualified designee) or the Sponsor, no longer justifies the subject’s participation in this study.
3. If any deviations occur during the conduct of the study. This may include the subject’s erroneous inclusion in the study. Any protocol deviations detected during the study should be corrected when possible and the subject should be allowed to continue. All protocol deviations will be fully documented and considered for their effect on study objectives. Deviations that could lead to subject discontinuation from the study include:

- deviations which could affect subject’s safety (e.g. illness requiring treatment[s]) which in the clinical judgement of the Investigator or their appropriately qualified designee (or after discussion with the Medical Monitor [details provided in Section 8.4]) might invalidate the study by interfering with the allocated test product or the willingness of the subject to comply with the study activities.
- deviations involving the use of any nicotine/tobacco products other than the intended conventional cigarettes or THP by subjects in Arms A and B. The level of non-compliance that results in subject withdrawal from the study will be determined by the Sponsor.
- deviations involving the use of any nicotine/tobacco products by Arm D (the cessation arm) after Visit 1 and before Check-out at Visit 13, excluding specified NRT/smoking cessation aids described in Section 3.4.2. The level of non-compliance that results in subject withdrawal from the study will be determined by the Sponsor.
- deviations involving the use of any nicotine/tobacco products by subjects in Arm E (never-smoked arm).

1. If the subject is uncooperative, including non-attendance. In these cases, efforts should have been made by the Investigator or their appropriately qualified designee to ascertain the reason and to ensure subject’s attendance as soon as possible.
2. Subject’s personal request: the subject could decide, at any moment of the study, to stop his/her participation. The Investigator or their appropriately qualified designee should ensure this is not due to AEs, in which case, this reason should be selected. Subjects do not have to provide a reason for withdrawing from the study if they do not wish to do so (reason for withdrawal should be recorded as ‘not given’).
3. Pregnancy.
4. Premature cancellation of the study.
5. If a subject in a smoking/THP arm (Arms A and B) decides to quit smoking following Screening until Check-out at Visit 13.
   1. Smoking Advice and Support
      1. All Subjects

Information will be provided for all subjects regarding the health risks associated with smoking. Advice on smoking cessation will be freely available to all subjects at Screening and during all clinic visits. The advice will be based on the recommendations of the WHO “Evidence based Recommendations on the Treatment of Tobacco Dependence”. Subjects who decide to quit smoking during the study period will be referred to the appropriate stop smoking services to support their cessation attempt.

- - 1. Smoking Cessation Arm (Arm D)

Subjects in Arm D will determine their smoking cessation strategy with the Investigator or their appropriately trained designee at Visit 1. If necessary, subjects will be provided with NRT and/or varenicline (Champix; 1 × 12 week course) or given a prescription for these products. Subjects will also be provided smoking cessation counselling at the site. For additional support, subjects will be referred to the following services based on where they live:

Subjects in England - National Health Service (NHS) quitting support website (https://www.nhs.uk/live-well/quit-smoking/nhs-stop-smoking-services-help-you-quit), the Smokefree helpline (Tel: 0300 123 1044), online advisor support, local stop smoking service, and Smokefree app.

- Subjects in Northern Ireland – Want2Stop quitting support website (www.want2stop.info)
- Subjects in Wales – NHS Wales quitting support website (www.helpmequit.wales) and telephone helpline (0800 085 2219)

Subjects will also be provided with a 24-hour site number that they contact for cessation support if required. The Investigator or their appropriately qualified designee will review subject’s progress and strategy at each clinic visit.

- 1. Management of Subject Safety

Before leaving the clinical site, each subject will be given a card to carry at all times in case of an emergency or should they otherwise require assistance outside the clinical site. The card gives details of the study number, start and end date of the subject’s involvement in the study, subject details, name of the relevant study physician and the address and telephone number of the clinical site. Subjects may destroy this card 4 weeks after Visit 13.

1. INVESTIGATIONAL PRODUCT

Details of the study product are presented in the following table:

| **Study Product** | **Product Code** | **Format** | **Product & Manufacturer** | **Nicotine Yield/Content** |
| --- | --- | --- | --- | --- |
| 1 | THP1.1(RT) | THP | Glo device with neostik, British American Tobacco | 0.68 mg/neostik^a^ |

^a^ nicotine yield under modified Health Canada Intense machine smoking regime; 55 mL puff volume; 30 second puff interval; 2 second puff duration; 8 puffs; no vent blocking.

The product is commercially available in Japan, Italy, Canada, Romania, South Korea, Russia, and Switzerland, and will be supplied by the Sponsor.

Prior to being distributed to subjects, study products will be kept in their packages and stored at room temperature under temperature monitored conditions. Subjects randomised to the THP arm will be provided with instructions for storage of the product. No special procedures are required for the safe handling of the products, other than ensuring that only the charger supplied with the THP is used to charge the device. All products must be kept out of sight and reach of children.

- 1. Identity of Investigational Products

Production lot number and date of manufacturing of the investigational products will be documented in the study file for products administered in Arm B.

The THP1.1(RT) THP device will be supplied by the Sponsor along with a micro-USB charger, a cleaning brush, and instructions for use. The device must only be charged as per the device instruction document.

Tobacco heating products, packs and cartons will be pre-labelled as per clinical sites standard operating procedures.

- 1. Accountability

Records will be maintained showing the receipt and disposition of the study supplies by the Investigator or their appropriately qualified designee. The Sponsor will be permitted at intervals and upon request during the study to check the supplies storage and assembly procedures and records at each clinical site.

Following completion of the clinical phase of the study and Sponsor review of accountability, the Investigator or their appropriately qualified designee will ensure that all unused supplies will either be returned to the Sponsor (together with the accountability records) or will be destroyed and Certificates of Destruction provided to the Sponsor, as directed by the Sponsor.

- 1. Blinding

This study will not be blinded.

- 1. Randomisation

Subjects in the continue-to-smoke/THP population will be randomised to Arm A or B. A randomisation scheme will be provided for the clinical sites. Each site will recruit a similar number of male and female subjects and a similar number of subjects ≤ 40 years old and > 40 years old at the time of randomisation.

Each site will receive 4 randomisation lists:

1. Males ≤ 40 years old.
2. Males > 40 years old.
3. Females ≤ 40 years old.
4. Females > 40 years old.

The allocation of subjects in each age/sex combination will be monitored on an ongoing basis during the study and if there is considered to be an imbalance between male and female subjects ≤ 40 years old and > 40 years old which could lead to problems when interpreting the data then the sites will be notified and will endeavour to prioritise the recruitment of subjects in the relevant category.

There will be no randomisation for subjects in the intend-to-quit population and never‑smoked populations; however, sites will endeavour to balance these populations in relation to male and female subjects and subjects ≤ 40 years old and > 40 years old.

- 1. Compliance

The study will be conducted in the ambulatory setting. Subjects will be required to comply with their randomised product or cessation aid, dependent on study arm, as indicated in Section 3.1.3.

Subjects will be instructed of the importance of complying with their randomised product (Arms A and B) or of not smoking cigarettes or using the THP (Arms D and E). Subjects will be asked to report any non-compliance via the automated eDiary (or paper diary if required) (Arms A, B and D) or using a paper diary (Arm E), and will be informed that compliance assessments will be conducted at selected clinic visits.

1. Selection of Study Population
   1. Selection of Study Population

Three populations will be recruited in total: 2 populations of smokers (Arms A, B, and D) and one population of subjects who have never smoked (Arm E).

For Arms A and B, 265 male or female subjects who have smoked 10 to 30 cigarettes per day, inclusive, commercially available and/or roll your own, non-menthol CPD, and who are willing to switch to a THP will be enrolled in this study. For Arm D, 190 male or female subjects who smoke 10 to 30, inclusive, commercially available and/or roll your own, non‑menthol CPD and who are intending to cease all non-medicinal nicotine use will be enrolled in this study. For these 2 populations, subjects will have smoked for at least 5 years consecutively. The subjects’ smoking status will be verified using a urinary cotinine test (cotinine >200 ng/mL) and an exhaled CO measurement (exhaled CO ≥ 7 ppm; subjects may be rescreened if CO levels are < 7 ppm).^19^

For Arm E, 40 subjects who have never smoked (< 100 cigarettes in their life and none within 30 days prior to Screening) will be recruited.

Each site will recruit a similar number of male and female subjects and a similar number of subjects ≤ 40 years old and > 40 years old, as described in Section 4.4.

- 1. Determination of Sample Size

A target of 50 completed subjects in each of the Arms A, B, and D has been set for this study. This is based on power for the primary objective of multiple between-arm comparisons. Calculations are based on the primary biomarker requiring the largest sample size (Augmentation Index [AIx]) between test and control products. Specifically, the power calculation was based on the number of subjects required to perform a contrast based on the F-statistic with 90% power between the arm using THP1 and the smoking arm at Day 360. The sample size was determined to be adequate based on AIx with expected means of 25.7 and 17. 5 for smoker and THP arms, and a common standard deviation of 12.4.^20^

This calculation assumes an 80% change with respect to that observed in subjects quitting smoking and an alpha level of 0.0451, adjusted for timepoint multiplicity using O’Brien‑Fleming sequential approach.

Thirty completed subjects in Arm E has been determined empirically; no formal statistical analysis of sample size has been performed.

- 1. Inclusion Criteria

Subjects will be required to satisfy all of the following criteria at the Screening Visit, unless otherwise stated:

All Subjects:

1. Subjects will be:
   1. males or non-pregnant, non-lactating females
   2. between 23 and 55 years of age, inclusive. Age verification will be performed by checking government issued identification (e.g. passport or driving licence) during Screening
2. Subjects will have a:
   1. body mass index (BMI) between 17.6 and 32.0 kg/m^2^, inclusive
   2. body weight exceeding 50 kg (males) or 40 kg (females)
3. Subjects will be in good health, as judged by the Investigator or their appropriately qualified designee based on:
   1. medical history
   2. physical examination
   3. vital signs assessment (blood pressure < 140 mmHg systolic)
   4. 12-lead ECG
   5. clinical laboratory evaluations
   6. lung function tests (Gold stage 1 is acceptable; see exclusion criterion 16)
4. Subjects will have given their written informed consent to participate in the study and will have agreed to abide by the study restrictions.
5. Subjects must demonstrate the ability to comprehend the Informed Consent Form (ICF), be able to communicate well with the Investigator or their appropriately qualified designee, understand and comply with the requirements of the study, and be judged suitable for the study in the opinion of the Investigator or their appropriately qualified designee.
6. Subjects will be willing to refrain from consuming alcohol within 24 hours prior to Screening and Check-in at each study visit, with the exception of the Follow-up Visit.
7. Subjects will be willing to refrain from consuming barbequed or chargrilled food, and avoid being in the presence of the barbequed or chargrilled food for 48 hours prior to Check-in at each study visit. Subjects will also be willing to avoid food containing poppy seeds for 3 days before both Screening and Check-in at each study visit.

Arms A, B, and D:

1. Subjects will be regular smokers of commercially manufactured filter cigarettes and/or roll your own cigarettes.
2. Subjects will have smoked for at least five consecutive years prior to Screening.
3. Subjects will typically smoke at least 10 and a maximum of 30 CPD and must have a urine cotinine level > 200 ng/mL and an exhaled breath CO level ≥ 7 ppm at Screening.
4. Subjects in Arm A who continue to smoke will be willing to use factory-manufactured non-mentholated cigarettes and/or roll your own cigarettes.
5. Subjects in Arm B will be willing to use the study product (THP) provided to them during the study.
6. Subjects in Arm D will be willing to abstain from smoking and using NGPs.

Arm E

1. Subjects will have never smoked (<100 cigarettes in their life and none within 30 days prior to Screening) and will continue to not smoke or use any form of tobacco or nicotine‑containing product (including THPs) for the duration of the study.
   1. Exclusion Criteria

Subjects will be excluded from the study if they satisfy any of the following criteria at the Screening Visit, unless otherwise stated:

All subjects

1. Male subjects who do not agree, or whose partners of childbearing potential do not agree, to use a barrier method of contraception (i.e. a condom with spermicide) or to refrain from donating sperm from Visit 1 until the end of the Follow-up Visit (see Section 7.1.7).
2. Female subjects of childbearing potential who do not agree to use a highly effective method of birth control in conjunction with male barrier method contraception (i.e. a condom with spermicide) from the time of signing the ICF until the end of the Follow-up Visit (see Section 7.1.7).
3. Female subjects who are pregnant or breastfeeding. This will be confirmed at Screening and Visit 1. Any female subject who becomes pregnant during this study will be withdrawn.
4. Subjects who have donated:
   1. ≥400 mL of blood within 12 weeks (male) or 16 weeks (female) prior to Visit 1.
   2. plasma in the 2 weeks prior to Visit 1.
   3. platelets in the 6 weeks prior to Visit 1.
5. Subjects who have an acute illness (e.g. upper respiratory tract infection) requiring treatment within 4 weeks prior to Visit 1 (subjects who had viral infections that resolved ≥2 weeks prior to Visit 1 will be admissible to this study).
6. Subjects who have a significant history of alcoholism or drug/chemical abuse within 24 months prior to Screening, as determined by the Investigator.
7. Subjects who have:
   1. a positive urine drugs of abuse screen (confirmed by repeat) at Screening or Visit 1.
   2. a positive alcohol breath test (confirmed by repeat) at Screening or Visit 1.
8. Subjects who:
   1. are carriers of the hepatitis B surface antigen (HBsAg).
   2. are carriers of the hepatitis C antibody.
   3. have a positive result for the test for human immunodeficiency virus (HIV) antibodies.
9. Subjects who have used prescription or over-the-counter (OTC) bronchodilator medication (e.g. inhaled or oral β-adrenergic agonists) to treat a chronic condition within the 12 months prior to Visit 1.
10. Subjects who have received any medications or substances (other than tobacco) which
    1. interfere with the cyclooxygenase pathway (e.g. anti-inflammatory drugs including aspirin and ibuprofen) within 14 days prior to Visit 1.
    2. are known to be strong inducers or inhibitors of cytochrome P450 (CYP) enzymes within 14 days or 5 half-lives of the drug (whichever is longer) prior to Visit 1.
11. Subjects who perform strenuous physical activity (exceeding the subject’s normal activity levels) within 7 days prior to Screening or Visit 1.
12. Subjects who are unable to communicate effectively with the Investigator/study staff (i.e. language problem, poor mental development, or impaired cerebral function).
13. Subjects who are unwilling or unable to comply with the study restrictions and requirements.
14. Employees and immediate relatives of the tobacco industry and the clinical site.
15. Subjects who are still participating in another clinical study (e.g. attending follow-up visits) or who have participated in a clinical study involving administration of an investigational drug (new chemical entity) in the past 3 months prior to first product use.
16. Subjects who have any clinically relevant abnormal findings on the physical examination, medical history, ECG, lung function tests (post-bronchodilator FEV_1_/FVC < 0.7 and FEV_1_ < 80% predicted value at post-bronchodilator spirometry or have an asthma condition [post-bronchodilator FEV_1_/FVC < 0.75 and reversibility in FEV_1R_ > 12% and > 200 mL from pre- to post-bronchodilator values at Screening]) or clinical laboratory panel, unless deemed not clinically significant by the Investigator or their appropriately qualified designee.
17. Subjects who have, or who have a history of, any clinically significant neurological, gastrointestinal, renal, hepatic, cardiovascular, psychiatric, respiratory, metabolic, endocrine, haematological or other major disorder that, in the opinion of the Investigator or their appropriately qualified designee, would jeopardise the safety of the subject or impact on the validity of the study results.
18. Subjects who have previously been diagnosed with any form of malignancy.
19. Subjects who have any clinically significant abnormal laboratory safety findings at Screening, as determined by the Investigator or their appropriately qualified designee (1 repeat assessment is acceptable).
20. Subjects who have previously been enrolled in or withdrawn from this study.
21. Subjects who, in the opinion of the Investigator, should not participate in this study.

Arms A, B, and D:

1. Subjects who regularly use any nicotine or tobacco product other than commercially manufactured filter cigarettes and/or roll your own cigarettes within 14 days of Screening.

Arms A and B:

1. Subjects who are self-reported non-inhalers (smokers who draw smoke from the cigarette into the mouth and throat but who do not inhale).
2. Subjects who, prior to randomisation, are planning to quit smoking in the next 12 months. All subjects will be informed that they are free to quit smoking and withdraw from the study at any time. Any subject who decides to quit smoking will be directed to appropriate stop smoking services (Section 3.4).
3. STUDY ACTIVITIES

A detailed Schedule of Assessments can be found in Appendix 15.1.

- 1. Screening
     1. Screening Visit

Subjects will attend a Screening Visit within 28 days prior to Visit 1. Prior to the Screening Visit, subjects will:

- refrain from strenuous physical activity (exceeding the subject’s normal activity levels) for 7 days.
- abstain from alcohol for 24 hours.

Subjects will be asked to sign the study‑specific ICF in the presence of a suitably trained clinical site operations staff member prior to any screening procedures being performed. The information recorded for all subjects, regardless of their suitability for the study, will be retained and archived in accordance with the Investigator’s data retention policies. All assessments conducted at the Screening Visit are presented in the Schedule of Assessments (Appendix 15.1).

All subjects in the continue-to-smoke/THP population will be shown the THP1.1(RT) THP products at Screening.

Subjects in Arm D (smoking cessation) who have passed all screening assessments and signed the ICF will be contacted prior to Visit 1 to discuss their smoking cessation strategy. Contact may take the form of a telephone call or site visit.

- - 1. Re-screening

Subjects who undergo screening but who are not enrolled will be eligible for re-screening at the discretion of the Investigator.

- 1. Visit 1

Prior to Check-in, all subjects will:

- Refrain from strenuous physical activity (exercise exceeding the subject’s normal activity levels) for 7 days.
- Abstain from alcohol for 24 hours.
- Adhere to dietary restrictions detailed in Section 7.1.3.
- Collect a 24-hour urine sample (to begin on Day -1 [AM] and to include the first void of the day on Day 1).

At Visit 1, all subjects will undergo the following demographic and safety assessments prior to randomisation (Arms A and B) or enrolment (Arms D and E):

- Review of inclusion/exclusion criteria
- Review of medical history
- Physical examination (symptom driven)
- Vital signs
- Resting 12-lead ECG
- Lung function test
- Urine drugs of abuse screen
- Urine cotinine test
- Alcohol breath test
- Review of AEs/SAEs
- Review of concomitant medication
- Clinical laboratory evaluations (the Investigator is not required to review these data before randomisation or enrolment).

For female subjects:

- Urine pregnancy test

Following randomisation (Arms A and B) or enrolment (Arms D and E), subjects will undergo the following baseline assessments:

- Blood sampling for primary endpoint biomarkers, BoE, BoBE, and exploratory endpoints
- Nasal transcriptomics sampling
- Carbon monoxide and NO breath tests
- Physiological endpoint assessments (body weight/waist circumference, carotid/femoral pulse wave velocity, 6-minute walking test, finger plethysmography)
- Questionnaire administration
  - 1. Continue-to-Smoke/Next Generation Product Population Only

Following safety assessments but prior to randomisation, product use training and demonstration will occur for subjects in the continue-to-smoke/THP population. Subjects will be offered the opportunity to trial up to 2 THP sticks. Product use will be separated by a minimum of 15 minutes during which subjects will not be allowed to use any nicotine‑containing products. The purpose of the product test is to allow subjects to experience the THP that they could be randomised to. If subjects decide that they do not wish to continue in the study following the product test, they will be withdrawn prior to randomisation. Subjects will be informed that there is an approximate 24.5% chance of being asked to continue to smoke their own brand cigarettes, and a 75.5% chance of being asked to smoke the THP.

Following the product test, subjects willing to take part in the trial will be randomised as described in Section 4.4. An additional, more detailed product use training and demonstration may be performed after randomisation, which may include, but may not be limited to, a demonstration of cleaning and storage instructions.

Prior to Check-out on Day 1, subjects randomised to Arm B will be provided with the investigational product.

Following randomisation, subjects will be instructed to use only their assigned product. As non-compliance with the study products may occur, there will be a 14 day transition period following Visit 1 where non-compliance is tolerated. After this period, subject non‑compliance will be assessed on an individual basis, and may lead to subject withdrawal as described in Section 3.3.

- - 1. Intend-to-quit Population

Subjects in the intend-to-quit population will be expected to refrain from nicotine-containing product use from the time of enrolment at Visit 1. Subjects will receive smoking cessation support as described in Section 3.4.2. It is anticipated that subjects may not stop using all tobacco products immediately following enrolment, the so the use of tobacco products will be tolerated during a 14 day transition period following Visit 1. After this period, use of tobacco products by a subject will be assessed on an individual basis, and may lead to subject withdrawal as described in Section 3.3.

- - 1. Never-smoked Population

Subjects in the never-smoked population (Arm E) will be instructed not to use tobacco or nicotine products for the duration of the study.

- 1. Visit 2 to Visit 13

Subjects in Arms A, B, and D will return to the clinic for non-residential visits every 30 days (+/-3 days for Visit 2 to Visit 4, +/- 2 weeks for Visit 5 to Visit 13). Subjects in Arm E will return to the clinic for non-residential Visit 4 (Day 90 +/- 3 days), Visit 7 (Day 180 +/- 14 days), and Visit 13 (Day 360 +/- 14 days).

Prior to Check-in at each visit, subjects will:

- Refrain from strenuous physical activity (exercise exceeding the subject’s normal activity levels) for 7 days.
- Abstain from alcohol for 24 hours.
- Adhere to dietary restrictions detailed in Section 7.1.3.
- Provide a 24-hour urine collection sample (to begin the day [AM] prior to Check-in for Visits 2, 3, 4, 7, 10, and 13 only).

Following Check-in, subjects will undergo the following assessments:

- Urine drugs of abuse screen
- Alcohol breath test

For female subjects:

- Urine pregnancy test

The following samples/assessments will be completed at the visits indicated in the Schedule of Assessments (Appendix 15.1):

- Blood sampling for BoE, BoBE, and exploratory endpoints
- Nasal transcriptomics sampling
- White blood cell transcriptomics sampling
- End of 24-hour urine collection
- Carbon monoxide and NO breath tests
- Physiological endpoint assessments (body weight/waist circumference, carotid/femoral pulse wave velocity, 6-minute walking test, finger plethysmography)
- Questionnaire administration
- Safety assessments

For subjects in Arm B, an assessment of product usage should be undertaken, and the allocation of product should be 120% of the product used in the previous period, up to a limit of 200%. At these visits, subjects will return all empty, part-used, and unused packs of THP consumables. At Visit 13, subjects will return all empty, part-used, and unused packs of THP consumables, and the study devices, charger, and other accessories supplied to them for this study.

- 1. Follow-up Contact
     1. Visits 1 to 12

All subjects in Arms B and D will be contacted by telephone 7 to 14 days after Visit 1 to aid randomised product (Arm B) or smoking cessation (Arm D) compliance. Subjects will be reminded of the support available (see Section 3.4), and will be able to speak to an appropriately trained member of staff to help to prevent combustible cigarette use.

At the discretion of the Investigator or their appropriately trained designee, additional telephone calls or clinical site visits may be scheduled for subjects in Arm D to monitor or update their smoking cessation strategy or prescriptions.

- - 1. Visit 13

All subjects will return to the site for a Follow-up assessment within 28 days after Check-out at Visit 13. The Investigator or their appropriately qualified designee will complete the following safety assessments:

- Physical examination (symptom driven)
- Vital signs
- 12-lead ECG
- Review of AEs/SAEs
- Clinical laboratory evaluations
- Review of concomitant medication.

For female subjects:

- Urine pregnancy test.

Provided there are no AEs which require further attention, the subject’s participation in the study will be complete. A further visit may be scheduled if deemed necessary by the Investigator or their appropriately qualified designee.

Subjects who, after enrolment, discontinue the trial prematurely should be encouraged to participate in this follow-up procedure. If their withdrawal is due to safety evaluations then the subjects should be followed until these return to baseline levels or until the Investigator or their appropriately qualified designee has determined that these events are no longer clinically significant.

Subjects who develop an AE at any time during the study will be followed until any required evaluations have returned to baseline or until the Investigator or their appropriately qualified designee has determined that these events are no longer clinically significant.

Reported AEs will be followed until resolution whenever possible.

- 1. Study Termination

The study may be discontinued at the discretion of the Investigator (or designee), Sponsor, or Sponsor’s Medical Monitor if any of the following criteria are met:

- adverse events unknown to date
- increased frequency, severity, and/or duration of known, anticipated, or previously reported AEs (this may also apply to AEs defined at Check‑in as baseline signs and symptoms)
- At Day 90, <25 subjects observe reductions ≥50% from baseline for at least 2 of the following biomarkers of exposure: CEMA, S-PMA, 2-AN
- medical or ethical reasons affecting the continued performance of the study
- difficulties in the recruitment of subjects
- administrative reasons
- cancellation of product development.

A written statement fully documenting the reasons for study termination will be provided to the ethics committee (EC).

1. STUDY PROCEDURES
   1. Specific Restrictions/Requirements
      1. Concomitant Medication

Subjects should avoid medication that interferes with the cyclooxygenase pathway (anti‑inflammatory drugs such as aspirin and ibuprofen) from 14 days prior to Visit 1 until Check-out at Visit 13.

Subjects should also not use any drugs or substances (except tobacco) known to be strong inducers or inhibitors of CYP enzymes (formerly known as cytochrome P450 enzymes) within 14 days or 5 half-lives of the drug (whichever is longer) prior to each clinic visit. A list of such drugs and substances known to modulate CYP enzymes can be found at the following location: http://medicine.iupui.edu/clinpharm/ddis/main-table/. If additional clarification is required, the Investigator or their appropriately qualified designee will contact the Medical Monitor.

During the study, subjects will be instructed not to take any medication, including over‑the‑counter products, without first consulting with the Investigator. The Investigator will contact the Medical Monitor for questions regarding episodic use. Proton pump inhibitors, antidepressants, hypnotics, and benzodiazepines are allowed. Antibiotics are allowed for episodic use, provided that they are not strong inhibitors or inducers of CYP enzymes (eg. ciprofloxacin, clarithromycin, erythromycin.). If a subject is taking a concomitant medication not listed in the protocol at Screening, eligibility should be discussed with the Medical Monitor before enrolling the subject.

If any medication is required, the name, strength, frequency of dosing and reason for its use will be documented in the subject's eCRF by the Investigator or their appropriately qualified designee on the appropriate page of the eCRF.

Herbal medications, vitamins, and supplements should be avoided for 3 days prior to each clinic visit, unless agreed as acceptable by the Investigator and/or Medical Monitor (details provided in Section 8.4).

- - 1. Smoking and Nicotine Use

Subjects will be asked to provide a urine sample for a cotinine screen at Screening and Visit 1 to ensure that they are currently using nicotine-delivering products (Arms A, B, and D) or that they are non-smokers and do not otherwise use nicotine-delivering products (Arm E). Subjects will also be asked to provide an exhaled breath CO test at Screening to confirm whether they have smoked conventional cigarettes. Subjects in Arms A, B, and D will only be enrolled into the study if their urine cotinine level is greater than 200 ng/mL and their exhaled breath CO level is ≥ 7 ppm. Subjects may be rescreened to confirm CO levels are ≥ 7 ppm if they have a low CO level at Screening.

- - 1. Diet

Subjects will fast for at least 6 hours prior to each study visit at which clinical laboratory evaluations will be performed. Subjects should also refrain from large meals, and caffeine‑containing food (eg, chocolate) and drinks for at least 4 hours before the AIx measurements, and all food and drink for 1 hour before NO measurements. Spirometry will also be conducted at least 2 hours after eating. At site visits, subjects may be offered a light meal following completion of fasted assessments.

Subjects will be advised to refrain from consuming barbequed or chargrilled food for 48 hours prior to Check-in for each study visit.

Subjects will be advised that they must not eat food containing poppy seeds for 3 days before both Screening and Check-in for each visit, as consumption of poppy seeds can lead to a positive opiate result in the drugs of abuse screen.

- - 1. Alcohol

Subjects will be instructed to refrain from consuming alcohol for 24 hours prior to clinic visits.

Subjects will be instructed not to consume on average more than 28 units of alcohol per week if male or 21 units of alcohol per week if female for the duration of the study.

- - 1. Exercise

Subjects will be requested not to undertake vigorous exercise outside of their usual routine from 7 days before clinic visits. Subjects will not undertake vigorous exercise (including the 6-minute walk test) within 30 minutes prior to spirometry assessments.

- - 1. Blood Donation

Subjects must not donate blood during the study or for 12 weeks (male) or 16 weeks (female) after the safety Follow-up Visit.

- - 1. Contraception

Female subjects participating in the study who are of non-childbearing potential will not be required to use contraception. Women of non-childbearing potential are defined as permanently sterile (i.e. due to hysterectomy, bilateral salpingectomy, bilateral oophorectomy, or confirmed tubal occlusion) or postmenopausal (defined as at least 12 months post-cessation of menses without an alternative medical cause). Postmenopausal status will be confirmed with a screening serum follicle-stimulating hormone (FSH) level greater than 40 mIU/mL.

Female subjects of childbearing potential must be willing to use a highly effective method of birth control (i.e. contraceptive measure with a failure rate of < 1% per year) in conjunction with male barrier contraception (i.e. male condom with spermicide) from Check-in at Visit 1 until the end of the Follow-up Visit. Highly effective methods of contraception include:

- Intrauterine device (IUD; e.g. Mirena®). Steel or copper IUDs are acceptable.
- Established use of oral, implantable, injectable, or transdermal methods of contraception associated with inhibition of ovulation.
- Male sterilization (performed at least 90 days prior to Screening), with verbal confirmation of surgical success. For female subjects on the study, the vasectomised male partner should be the sole partner for that subject.
- Bilateral tubal ligation (performed at least 90 days prior to Screening).

Male subjects will be surgically sterile for at least 90 days, or when sexually active with female partners of childbearing potential will be required to use a male condom with spermicide from Check-in to Visit 1 until after the Follow-up Visit. Sexual intercourse with female partners who are pregnant or breastfeeding should be avoided unless condoms are used from Check-in at Visit 1 until the end of the Follow-up Visit. Male subjects are required to refrain from donation of sperm from Check-in at Visit 1 until the end of the Follow-up Visit.

Subjects who practice true abstinence, because of the subject’s lifestyle choice (i.e. the subject should not become abstinent just for the purpose of study participation), are exempt from contraceptive requirements. Periodic abstinence (e.g. calendar, ovulation, symptothermal, post-ovulation methods) and withdrawal are not acceptable methods of contraception. If a subject who is abstinent at the time of signing the ICF becomes sexually active they must agree to use contraception as described above and as outlined in the ICF.

For subjects who are exclusively in same sex relationships, contraceptive requirements do not apply. If a subject who is in a same sex relationship at the time of signing the ICF becomes engaged in a heterosexual relationship, they must agree to use contraception as described above and as outlined in the ICF.

- 1. Clinical Assessments

The timings of all measurements to be performed during the study may be subject to change based on the ongoing review of the safety and tolerability results.

- - 1. Demographic Data

Socio-demographic data (sex, age, race, and ethnicity) will be recorded at the Screening Visit.

- - 1. Smoking History and Willingness to Quit Smoking

Subjects will be asked about their smoking history at Screening. This will include questions to: evaluate whether the subject has smoked for at least the last 5 consecutive years, determine the number of cigarettes smoked by the subject per day on average, check that the subject has smoked their chosen brand of conventional cigarette consistently for at least 6 months, and to determine if the subject regularly uses any nicotine or tobacco product other than commercially manufactured filter cigarettes and/or roll your own cigarettes. In addition, the subject will be asked if he/she is planning to quit smoking within the next 12 months. A colour photocopy or photograph of each subject’s usual cigarette pack, including surface showing product details, will be taken.

- - 1. Medical History and Concomitant Diseases

Relevant medical history, as determined by the Investigator or their appropriately qualified designee, will be documented at Screening and Check-in at Visit 1. Medical history will include any clinically significant neurological, gastrointestinal, renal, hepatic, cardiovascular, psychiatric, respiratory, metabolic, endocrine, haematological or other major disorders. Medical history is defined as any condition that started and ended prior to Screening. A concomitant disease is any disease that started prior to and which was still ongoing at Screening.

- - 1. Vital Signs

Supine blood pressure, supine pulse rate, respiratory rate and axillary body temperature will be measured at the times indicated in the Schedule of Assessments (Appendix 15.1). Vital signs will also be performed at other times if judged to be clinically appropriate.

Blood pressure and pulse rate will be measured using automated monitors. Subjects must be supine for at least 5 minutes before blood pressure and pulse rate measurements.

Respiratory rate will be measured as per the standard institutional practice.

Axillary body temperature will be measured singly using a digital thermometer.

- - 1. Physical Examination

A full physical examination will be performed at Screening, and a symptom driven physical examination will be performed at the times indicated in the Schedule of Assessments (Appendix 15.1).

- - 1. Height, Body Weight, and BMI

Height in meters (to the nearest cm), and weight in kilograms (to the nearest 0.1 kilogram) in underclothing and without shoes will be measured at Screening.

The BMI at Screening will be calculated using the following formula:

BMI = body weight (kg)
 [height (m)]^2^

- - 1. Electrocardiography

A single 12‑lead resting ECG will be recorded after the subject has been supine for at least 5 minutes at the times indicated in the Schedule of Assessments (Appendix 15.1). The 12‑lead ECG will be repeated once if either of the following criteria applies:

- The QT interval corrected for heart rate using Fridericia’s method (QTcF) is >500 msec
- The QTcF change from the baseline (pre-product use) is >60 msec

If repeated, the repeat values will be used for data analysis.

Additional 12‑lead ECGs will be performed at other times if judged to be clinically appropriate or if the ongoing review of the data suggests a more detailed assessment of ECGs is required.

A physician will perform a clinical assessment of each 12-lead ECG. Clinical site reference ranges will be applied to all ECG parameters determined throughout the study.

The ECG machine will compute the PR and QT intervals, QTcF, QRS duration and heart rate.

- - 1. Lung Function Tests

Spirometry pre and post short-acting bronchodilator will be conducted, as indicated in the Schedule of Assessments (Appendix 15.1). At Screening, post‑bronchodilator spirometry (salbutamol; 4 x 100 µg, administered using a spacer) will be conducted 15 minutes (± 5 minutes) after completion (last attempt) of pre‑bronchodilator spirometry. At Screening, spirometry will be conducted at least 1 hour after smoking, at least 2 hours after eating, and at least 30 minutes after any vigorous exercise.

Spirometry will be used to measure:

- Peak flow
- FVC
- Forced expiratory flow 25‑75% (FEF 25-75%)
- FEV_1_

Spirometry testing will be performed in accordance with procedures of the American Thoracic Society/European Respiratory Society.^21^ Spirometry predicted values will be standardized to the Global Lungs Initiative predictive set.^22^

Measurements will be repeated if there are technical issues during testing or if the subject shows signs of fatigue. Measurements will not be repeated if the results fall under the levels required for enrolment in the study (Section 5.4). If a subject shows signs of fatigue during repeated testing, testing will be halted to ensure that the subject does not become exhausted and will only be recommenced at the physician’s discretion. No more than 8 manoeuvers should be performed. The spirometry traces will be copied and inserted into the source documents. The spirometer must be kept calibrated as recommended by the manufacturer.

- - 1. Clinical Laboratory Evaluations

Blood and urine samples will be collected for clinical laboratory evaluations at the times indicated in the Schedule of Assessments (Appendix 15.1). Subjects will fast for at least 6 hours prior to blood collection for clinical laboratory evaluations. Subjects may drink water during this fasting period.

Additional clinical laboratory evaluations will be performed at other times if judged to be clinically appropriate or if the ongoing review of the data suggests a more detailed assessment of clinical laboratory evaluations is required.

The Investigator or their appropriately qualified designee will perform a clinical assessment of all clinical laboratory data.

Blood samples will be taken for the clinical laboratory panel. Details of all blood samples collected in this study are presented in Section 7.4.2.The following evaluations will be performed:

| **Serum biochemistry:** | **Units** | **Haematology:** | **Units** |
| --- | --- | --- | --- |
| Aspartate aminotransferase (AST) | IU/L | Haemoglobin | g/dL |
| Alanine aminotransferase (ALT) | IU/L | Haematocrit (packed cell volume [PCV]) | % |
| Alkaline phosphatase | IU/L | Total and differential leukocyte count | 10^9^/L & % |
| Gamma‑glutamyl transferase (GGT) | IU/L | Red blood cell (RBC) count | 10^12^/L |
| Sodium | mmol/L | Platelet count | 10^9^/L |
| Potassium | mmol/L | Mean cell volume (MCV) | fL |
| Chloride | mmol/L | Mean cell haemoglobin (MCH) | pg |
| Calcium | mmol/L | MCH concentration (MCHC) | g/dL |
| Inorganic phosphate | mmol/L | **Urinalysis:** | **Units** |
| Glucose | mmol/L | Microscopic examination | + |
| Urea | mmol/L | pH | NA |
| Uric acid | µmol/L | Specific gravity | NA |
| Total bilirubin | μmol/L | Protein | + |
| Direct bilirubin | μmol/L | Glucose | + |
| Creatinine | μmol/L | Ketones | + |
| Total protein | g/L | Bilirubin | + |
| Albumin | g/L | Blood | + |
| Total cholesterol | mmol/L | Nitrite | neg/pos |
| Triglycerides | mmol/L | Urobilinogen | + |
| Creatinine phosphokinase (CPK) | IU/L | Leukocytes | cells/field |
| Blood urea nitrogen (BUN) | mmol/L | Urine pregnancy test^d^ | neg/pos |
| **Serology^a^:** | **Units** | Urine drug screen^e^ | neg/pos |
| Hepatitis B surface antigen (HBsAg) | neg/pos | **Additional Tests** |  |
| Hepatitis C antibody | neg/pos | Alcohol breath test | neg/pos |
| Human immunodeficiency virus (HIV)^b^ | neg/pos |  |  |
| **Hormone Panel^c^:** | **Units** |  |  |
| Follicle‑stimulating hormone (FSH) | mIU/L |  |  |
| Human chorionic gonadotropin (hCG; serum pregnancy test) | IU/L |  |  |

^a^ Screening only
^b^ HIV1/2 and p24 antigen
^c^ In all females at Screening only
^d^ Female subjects only. A positive urine pregnancy test will be confirmed with a serum pregnancy test. See Schedule of Assessments in Appendix 15.1 for scheduled timepoints.
^e^ Urine drugs of abuse screen will be conducted for amphetamines, barbiturates, benzodiazepines, cocaine, ecstasy, methamphetamine, morphine, methadone, tricyclic antidepressants, and tetrahydrocannabinol.
neg = negative
pos = positive

- - 1. Urine Drugs of Abuse Screen and Alcohol Breath Test

Subjects will be asked to provide urine samples for drugs of abuse screens at the times indicated in the Schedule of Assessments (Appendix 15.1). Urine samples will be screened for the presence of the following drugs of abuse: amphetamines, barbiturates, benzodiazepines, cocaine, ecstasy, methamphetamine, morphine, methadone, tricyclic antidepressants, and tetrahydrocannabinol.

Subjects will be screened for the presence of breath alcohol at the times indicated in the Schedule of Assessments (Appendix 15.1).

- - 1. Body Fat Analysis

Subjects’ body fat will be measured at the times indicated in the Schedule of Assessments (Appendix 15.1) using an OMRON BF511 machine according to the manufacturer’s instructions. The following parameters will be calculated:

- Body fat (%)
- Visceral Fat (up to 30 levels)
- BMI
- Skeletal Muscle (in %)

Body fat measurements will be taken at least 2 hours after meals.

- 1. Sample Collection for Biomarker and Exploratory Endpoint Assessments
     1. Exhaled Carbon Monoxide and Nitric Oxide

Exhaled carbon monoxide (CO) will be measured using the EC50 Micro III Smokerlizer (Bedfont) CO meter, or similar device. Exhaled NO will be measured using an Aerocrine NIOX VERO device. Subjects will not be allowed to use any study products/smoke any cigarettes or eat within 30 minutes prior to CO or NO level measurements. Subjects should also avoid all food and drink for 1 hour prior to NO measurements. Levels will be measured at the times indicated in the Schedule of Assessments (Appendix 15.1).

- - 1. Urinary BoE and BoBE

Spot urine samples will be used for the urine drugs of abuse screen, cotinine test, urine pregnancy test, and safety urinalysis.

Pooled 24-hour urine samples will be collected for analysis of urinary BoE and BoBE. Creatinine in urine will also be calculated and used to report BoBE as amount per unit of creatinine.

Samples will be transported and stored as detailed in Section 7.4.

- - 1. Blood Sampling for BoE, BoBE, and Exploratory Endpoints

Blood samples will be collected as described in the laboratory manual. Samples will be transported and stored as detailed in Section 7.4.

- 1. Sample Handling, Transport and Storage

All samples (urine and blood) will be transported on dry ice for analysis, with replicate samples being shipped independently.

- - 1. Urine Samples

For BoE and BoBE analyses, urine samples will be collected over concurrent 24-hour time periods beginning on the day before each study visit. All urine voided during the 24‑hour period will be collected and samples aliquoted for the various analyses. The samples will be refrigerated at between 2°C to 8°C. Additional sample handling instructions will be provided in the laboratory manual.

The urine samples collected during a single 24-hour interval will be pooled together and weighed. The samples will be related to the day on which the 24-hour collection period ended. The pooled samples will be thoroughly mixed before providing aliquots for analyses. The total volume of all urine collected over each 24-hour period will be calculated from weight and specific gravity. Urine aliquots will be stored in suitably labelled tubes at the temperatures indicated in the laboratory manual pending assay.

- - 1. Blood Samples

Blood samples will be collected by qualified and trained site personnel. The following blood volumes will be withdrawn for each subject:

|  | **Volume per Blood Sample (mL)** | **Maximum Number of Samples** | **Total Amount of Blood (mL)** |
| --- | --- | --- | --- |
| Serum biochemistry and hematology^1^ | 7.5 | 7 | 52.5 |
| Serology | 3.5 | 1 | 3.5 |
| White blood cell count | 2.0 | 14 | 28.0 |
| s-ICAM1 | 4.0 | 10 | 40.0 |
| HDL and LDL | 2.0 | 14 | 28.0 |
| Cholesterol and triglycerides^2^ | 1.0 | 4 | 4.0 |
| CEVal (Hb adduct) | 10.0 | 7 | 70 |
| Other BoBE | 5.0 | 10 | 50.0 |
| Serum NMR lipoprotein and metabolomics, and plasma BH2/BH4 ratio | 18.0 | 6 | 108 |
| Transcriptomics (WBC) | 5.0 | 4 | 20.0 |
|  | | **Total:** | **404** |

Abbreviations: BH2/BH4 = dihydrobiopterin/tetrahydrobiopterin; BoBE = biomarker of biological effect; CEVal = N-(2-cyanoethyl)valine; Hb = haemoglobin; HDL = high-density lipoprotein; LDL = low-density lipoprotein; NMR = nuclear magnetic resonance; s‑ICAM1 = soluble intracellular adhesion molecule-1; WBC = white blood cell.
Blood samples will be destroyed as per the laboratory’s standard procedures.
^1^ Glucose, total cholesterol, and triglycerides will be measured as part of serum biochemistry assessments. Serum biochemistry tests include follicle-stimulating hormone and serum pregnancy tests.
^2^ Samples will be collected specifically for cholesterol and triglyceride assessment at Visits 2 and 3.

Subjects will be fasted, will avoid caffeine (Section 7.1.3), and will not smoke (Arms A, B, and D) for 2 hours prior to collection of blood samples.

The exact volumes of each sample may change but the total volume of blood drawn for any subject will not exceed 400 mL.

- 1. Other Study Assessments
     1. Physiological Assessments
        1. Body Weight and Waist Circumference

Body weight in underclothing and without shoes and waist circumference will be measured at the timepoints indicated in the Schedule of Assessments (Appendix 15.1).

- - - 1. Augmentation Index and Carotid/Femoral Pulse Wave Velocity

Augmentation index and carotid/femoral pulse wave velocity assessments will be conducted at the timepoints indicated in the Schedule of Assessments (Appendix 15.1) using a SphygmoCor XCEL device according to the manufacturer’s instructions.

Prior to measurements, subjects will fast and avoid caffeine for 4 hours (described in Section 7.1.3, and will not smoke (Arms A and B) for 2 hours. Subjects will have rested for 10 minutes in a supine position prior to measurements.

- - - 1. 6-minute Walking Test

A 6-minute walking test will be conducted at the timepoints indicated in the Schedule of Assessments (Appendix 15.1) in accordance with the European Respiratory Society/American Thoracic Society guidelines.^23^

Subjects will be asked to walk the greatest distance they can in 6-minutes without jogging or running. Subjects will be instructed to wear appropriate clothing, and will be monitored throughout the test by a suitably qualified member of staff. Subjects are permitted to eat a light meal prior to the test occurring, and will be required to rest in a seated position for 10 minutes prior to the start of the test. During the test, subjects will regulate their pace, and can slow down or stop if required. Subjects blood oxygen saturation levels (SpO_2_) will be monitored continually during the 6-minute walking test using a wrist-mounted pulse oximeter.

- - - 1. Finger Plethysmography

Finger plethysmography will be conducted at the timepoints indicated in the Schedule of Assessments (Appendix 15.1) using an EndoPAT^TM^ device and software according to the manufacturer’s instructions.

Subjects will be fasted, will avoid caffeine (Section 7.1.3), and will not smoke (Arms A, B, and D) for at least 2 hours prior to measurements. Subjects will have rested for 10 minutes in a supine position prior to measurements.

- - 1. Questionnaires

A paper copy of the subject questionnaires used in this study will be completed by each subject, and the Investigator/clinical site staff will review each questionnaire for completeness. Subjects will be required to answer all questions on each questionnaire. Questionnaires will be provided in English.

- - - 1. Tobacco Use History (All Subjects)

A tobacco use history questionnaire will be administered to all subjects at Screening only.

- - - 1. Fagerström Test for Cigarette Dependence (Arms A, B, and D Only)

Potential nicotine dependence will be assessed via a questionnaire at Screening and the study visits indicated in the Schedule of Assessments (Appendix 15.1) using a **FTCD questionnaire in its revised version.**^24^

**The questionnaire consists of 6 questions which will be answered by the subject himself/herself. The scores obtained on the test permit the classification of nicotine dependence into 3 levels: Mild (0-3 points), moderate (4-6 points), and severe (7‑10 points).**

- - - 1. Product Satisfaction (Arms A and B Only)

The Product Satisfaction questionnaire will be administered to subjects at the study visits indicated in the Schedule of Assessments (Appendix 15.1).

The questionnaire consists of 1 question, “Can you tell me how much do you like this tobacco product?” which will be answered by the subject himself/herself. The subject will provide responses on a 7-point Likert scale ranging from “1 – I dislike it a lot” to “7 – I like it a lot” (scores of 2 – 6 will not have a descriptor).

- - - 1. Smoking Cessation Quality of Life (Arms B and D only)

The impact of smoking cessation on perceived functioning and well-being will be assessed via a questionnaire administered to subjects at the study visits indicated in the Schedule of Assessments (Appendix 15.1), using the Smoking Cessation Quality of Life questionnaire.^25^

The Smoking Cessation Quality of Life questionnaire contains 5 multiple-item cessation‑targeted scales: social interactions, self-control, sleep, cognitive functioning and anxiety.

- - - 1. Cough and Shortness of Breath (All Subjects)

Cough and shortness of breath will be assessed using a visual analog scale (VAS) at the visits indicated in the Schedule of Assessments (Appendix 15.1).

- - - 1. Product Use Count (Arms A, B, and D Only)

The number of conventional cigarettes smoked or products used will be recorded daily between Days 1 and 360 using an eDiary. If the eDiary is not able to be used, subjects will record cigarette or product use using a paper diary.

1. ADVERSE EVENTS
   1. Definitions
      1. Adverse Events

The FDA modified risk tobacco product (MRTP) guidelines^26^ specify the following definition for AEs for tobacco products: an AE is any health-related event associated with the use of tobacco product in humans, which is adverse or unfavourable, whether or not it is considered related to the tobacco product, as defined by the MRTP guidelines.

An AE is defined as any untoward medical occurrence in a subject administered a study product, which does not necessarily have a causal relationship with the study product. An AE can therefore be any unfavourable and/or unintended sign (including an abnormal laboratory finding), symptom or disease temporally associated with the use of a study product, whether or not related to the study product.

- - 1. Serious Adverse Events

The FDA MRTP guidelines^26^ specify the following definitions for SAEs for tobacco products:

- results in death
- is life threatening
- requires inpatient hospitalisation or prolongation of existing hospitalisation
- results in persistent or significant disability/incapacity (disability is defined as a substantial disruption of a person’s ability to conduct normal life functions)
- is a congenital anomaly/birth defect

An important medical event that may not result in death, be life threatening or require hospitalisation may be considered a serious adverse experience when, based upon appropriate medical judgement, it may jeopardise the subject or may require medical or surgical intervention to prevent one of the outcomes listed in this definition. Examples of such medical events include allergic bronchospasm requiring intensive treatment in an emergency room or at home, blood dyscrasias or convulsions that do not result in inpatient hospitalisation, or the development of dependency or abuse.

Instances of death or congenital abnormality, if brought to the attention of the Investigator or their appropriately qualified designee at any time after last product use and considered by the Investigator or their appropriately qualified designee to be possibly related to the study product, will be reported to the Sponsor.

Definition of Life Threatening

An AE is life threatening if the subject was at immediate risk of death from the time of the event as it occurred; i.e. it does not refer to an event that might have caused death if it had occurred in a more serious form.

Definition of Hospitalisation

Adverse events requiring hospitalisation should be considered serious. In general, hospitalisation signifies that the subject has been detained (usually involving an overnight stay) at the hospital or emergency ward for observation and/or treatment that would not have been appropriate at the clinical site. When in doubt as to whether hospitalisation occurred or was necessary, the AE should be considered serious.

Hospitalisation for elective surgery or routine clinical procedures, which are not the result of an AE, need not be considered AEs and should be recorded on a clinical assessment form and added to the eCRF. If anything untoward is reported during the procedure, this must be reported as an AE and either ‘serious’ or ‘non-serious’ attributed according to the usual criteria.

- 1. Assessment of Adverse Events

The condition of each subject will be monitored from the time of signing the ICF, to Check‑out at the Follow-up Visit. In addition, any signs or symptoms will be observed and elicited at each visit by open questioning, such as “How have you been feeling since your last visit ?”

Subjects will also be encouraged to spontaneously report AEs occurring at any other time during the study.

Any AEs and remedial action required will be recorded in the subject's source data. The nature, time of onset, duration and severity will be documented, together with the Investigator’s opinion of the relationship to product use.

Any clinically significant abnormalities identified during the course of the study will be followed-up until they return to normal or can be clinically explained.

- 1. Intensity of Adverse Events

The causal relationship between an AE and the study product is defined as follows:

**Not Related** The AE is definitely caused by the subject's clinical state or the study procedures/conditions.

**Unlikely Related** The temporal association between the AE and the product is such that the product is not likely to have any reasonable association with the AE.

**Possibly Related** The AE follows a reasonable temporal sequence from the time of product use but could have been produced by the subject's clinical state or the study procedures/conditions.

**Related** The AE follows a reasonable temporal sequence from the time of product use, abates upon discontinuation of the product and reappears when the product is reintroduced.

The severity of an AE will be recorded as one of the following:

**Mild** Easily tolerated; does not interfere with normal daily activities; does not require intervention

**Moderate** Causes some interference with daily activities; minimal, local or non‑invasive intervention indicated

**Severe** Medically significant event; daily activities limited or completely halted; hospitalisation or prolongation of hospitalisation indicated

Every reasonable effort will be made to follow-up subjects who have an AE at Follow-up, if possible, until resolution.

Any AE assessed as related to the study product will be assessed for its expectedness. An AE will be regarded as ‘unexpected’ if its nature or severity is not consistent with information already known about the study product, and is not listed in the current Investigator’s Brochure (IB).^19^ The IB provides further detail on signs or symptoms that might be expected with the use of the study product, including information relating to malfunction or misuse.

- 1. SAE Reporting

All AEs occurring during this clinical study will be recorded from time of the subject signing the ICF until the end of the study. The Investigator or their appropriately qualified designee will review each event, grade its severity and assess its relationship to the product consumption and/or the study procedures. The date of onset, time of onset, and outcome of each event will be noted.

If any of the above AEs are serious, special procedures will be followed. The Investigator will report all SAEs to the Covance Pharmacovigilance and Drug Safety Services Department within 24 hours. This information must be sent by fax by filling out the serious AE form. Written reports must then be submitted within 48 hours, whether or not the serious events are deemed related to study procedures or products. The EC will be notified by these reports.

If an SAE occurs during this study, the Medical Monitor will be contacted.

- - 1. Abnormal Results of Laboratory Tests

Any clinical safety laboratory results that are outside of the normal reference range will be reviewed for clinical significance by the Investigator or their appropriately qualified designee. If the abnormal result is considered to be of clinical significance, it will be recorded as a concomitant disease (prior to enrolment) or an AE (after enrolment). As for other AEs, the relationship to the study product, intensity, seriousness and outcome of abnormal clinical safety laboratory results will be recorded.

- - 1. Abnormal Results of Other Tests and Investigations

Any ongoing medical conditions or clinically relevant findings detected at Screening will be considered to be a concomitant disease and the subject’s eligibility to participate in the study will be reviewed. Any new, clinically relevant, abnormal findings or worsening of pre‑existing conditions detected during the study after the Screening Visit until the end of the study will be documented as an AE.

- 1. Reporting and Follow-up of Pregnancies

For pregnancies detected during the Screening Period and prior to first product use, the subject will be considered a screening failure and removed from the study. The diagnosed pregnancy will be captured on the screening failure eCRF.

All pregnancies occurring after signing the ICF and diagnosed after enrolment until the completion of the study must be reported to the Investigator. Any pregnancy potentially associated to exposure to the study product, including pregnancies spontaneously reported to the Investigator or their appropriately qualified designee after the end-of-study must be reported and followed-up. Potential association with exposure to the study product is defined as the conception date being calculated before the last exposure to the study product.

The Investigator will complete a Pregnancy Form for all pregnancies (including positive urine pregnancy tests). Pregnancies must be reported to the Medical Monitor, according to their standard procedures.

- 1. Adverse Events Leading to Withdrawal

Subjects who are withdrawn from the study because of an AE will undergo the following assessments and will return for a Follow-up Visit:

- Physical examination (symptom driven)
- Vital signs
- 12-lead ECG
- Review of AEs/SAEs
- Lung function tests
- Clinical laboratory evaluations
- Urine pregnancy test (female subjects only)

The Investigator will follow-up any AE until they have resolved, stabilized or an acceptable explanation has been found.

- 1. Investigational Product Misuse

Any occurrence of THP misuse by a subject will be documented by site staff. Misuse is defined as actions not in accordance with the product instructions.

Investigational product misuse may result in use-related hazards.

Use-related hazards are derived from the US FDA Medical Device Use Safety Guidance:^27^

- Hazards caused specifically by how a device is used.
- Unanticipated use scenarios (e.g. applying any chemicals, using conventional cigarettes, mechanical damage of the product) that result in hazards must be documented and reported by the Investigator.

According to FDA Medical Device Regulation, data should be collected regarding the use‑related hazards that have occurred with the device and when information pertaining to device use safety is extensive, it is helpful to provide it in summary form that highlights the most important issues, considerations, resolutions, and conclusions. The level of detail of device use documentation submitted should be consistent with the level of concern of use‑related hazards for the device.

- 1. Investigational Device Malfunctions

Subjects will be provided with 2 identical devices which may be used concurrently. Subjects should report any device malfunctions, loss, or damage immediately to the site.

Any occurrence of malfunction of the THPs will be documented by the site staff and reported to the Sponsor as appropriate. Any apparently malfunctioning device will be removed from use and replaced.

1. DATA ANALYSIS
   1. General Considerations

Covance will perform the data analysis. The results of the study will be reported in a clinical study report. A detailed Statistical Analysis Plan describing the methodology to be used will be issued to the Sponsor for review and finalised prior to database lock.

In general, continuous variables will be presented by means of descriptive statistics (n, mean, standard deviation, median, minimum and maximum) and categorical variables will be displayed by means of frequency tables and, where appropriate, shift tables. All summaries will be presented by study arm, sex, age, and timepoint.

All data collected for the study will be presented as data listings for each subject.

- 1. Analysis Populations

The statistical analysis will be based on separate, hierarchically organised analysis populations defined as the following:

**Safety population** - All subjects who smoked at least one cigarette or had at least 1 safety assessment on Day 1.

**Intent-to-treat population (ITT)** - All subjects who were assigned and had at least 1 valid assessment of a biomarker variable.

**Per-protocol population (PP)** - All subjects who had a valid assessment of a biomarker variable and completed study according to the protocol (no major protocol deviations).

All listings will be based on the safety population. The baseline and demographics data will be summarised on all 3 populations. The safety data will be summarised based on the safety population only and the remaining data will be summarised for both the ITT and PP populations.

If there is less than a 10% difference between the ITT and PP populations then the summaries will only be based on the PP population.

- 1. Statistical Analysis of Primary and Secondary Objectives

The primary objective will be examined by computing levels of biomarkers at each timepoint, i.e. baseline, 90, 180, and 360 days. These data will be compared between the THP arm (Arm B) and the main control arm (Arm A) using specific contrast tests from statistical models adjusted for baseline measurements. Data will be examined and may be transformed to ensure that any assumptions associated with statistical tests or models are obeyed. Alpha level across timepoints has been adjusted using the O’Brien-Fleming approach, with 0.0471 overall available alpha at Day 360, 0.0151 at Day 180, and 0.0006 at Day 90.^28^ The significance level has been allocated at each primary endpoint based on likelihood of success to detect a significant change in biomarker levels. At Day 360, statistical comparisons between THP and control will be performed at α= 0.0451 for AIx. The remainder alpha will be distributed equally (α= 0.001) for the other 2 primary endpoints. At Day 90, only changes in BoE are expected; therefore, only Total NNAL will be statistically assessed with α=0.0006**.** For the statistical analysis performed at Day 180, an overall α level of 0.0151 will be equally distributed between the 3 primary endpoints (0.00503). If any endpoint were to be significant at Day 90 or 180, it will not be statistically assessed at Day 180 and/or 360, as appropriate, and its assigned alpha level will be equally distributed between the remaining primary endpoints.

Similarly, biomarker measures in the secondary objectives will be examined by computing levels of biomarkers at each timepoint. These data will be compared between the THP and the main control arm using specific contrast tests from statistical models adjusted for baseline measurements. Statistical comparisons for secondary endpoints will be only performed if any of the primary endpoints is significant and using the alpha level released by primary endpoints. If the statistical comparison for Total NNAL is significant at Day 90, only the secondary BoE will be analysed at Day 90 using a 0.0006 significance level. Multiplicity adjustment for family wise error of secondary endpoints will be performed using Holm’s method. If any secondary endpoint were to be significant at Day 90 or 180, it will not be statistically assessed at Day 180 and/or 360, as appropriate.

Product use compliance is a critical part of this study, as failure to fully replace cigarettes with the THP product would reduce or cancel the expected biomarker changes that would be observed if the product is used as indicated. To aid compliance assessment we will use a haemoglobin adduct of acrylonitrile, CEVal. Acrylonitrile is below the detection limit in the THP product emissions but can be found in cigarette smoke. We will use different thresholds for CEVal in ancillary analyses to deduce product use compliance. These thresholds have been calculated based on a previous study where this biomarker was reported for a modified combustible prototype cigarette.

The interim analysis on Day 90 will be performed on a subset of subjects who were enrolled on to the study on or before the day that the 42^nd^ subject was enrolled on to Arm A, and were still enrolled on the study at Day 90. This was chosen to ensure that 30 subjects on Arm A were still enrolled on the study at Day 90 to give sufficient power to detect the statistical difference between the two arms for Total NNAL.

- 1. Safety Data Summary and Analysis

All safety data will be summarised for all safety parameters based on the safety population, stratified by study arm.

1. REPORTS AND PUBLICATIONS
   1. Reports to the Ethics Committee

Upon completion of the study, the Coordinating Investigator or their appropriately qualified designee will inform the EC of the study’s completion and outcome.

The Medicines and Healthcare products Regulatory Agency (MHRA) will be provided with a Declaration of the End of a Clinical Trial form and safety report (within 90 days of completion or within 15 days of a premature termination). (These will also be provided to the EC.)

- 1. Clinical Study Report

Covance will prepare interim presenting data generated over the initial 90 days of the study, an interim presenting data generated over the initial 180 days of the study, and an integrated clinical study report following study completion. Prior to issuing the final clinical study report, Covance will prepare a draft report for approval by the Sponsor. The report will be in accordance with the International Council for Harmonisation (ICH) Note for Guidance on Structure and Content of Clinical Study Reports.

The draft report may be submitted for quality assurance audit, the findings of which will be incorporated into the final version.

An electronic copy of the final report will be provided to the Sponsor. The study report will be provided in PDF format unless otherwise agreed by Covance. Covance will accept no responsibility for subsequent operations carried out on this electronic information, or copies thereof, after delivery to the Sponsor. Reports requiring specialised Sponsor formats/alternative computer software packages may be possible on request from the Sponsor but may involve extra time and cost.

Delivery of a full hard copy of the final report may be possible on request from the Sponsor but may involve extra time and cost.

- 1. Publication and Disclosure Policy

All data collected during the study will be the property of the Sponsor.

This document contains sensitive data and information that are confidential and proprietary to the Sponsor. This document is being provided solely for the purpose of evaluation and/or conducting this clinical study for the Sponsor. Disclosure of the content of this document is allowed only to study personnel, EC, or duly authorised representatives of regulatory agencies for this purpose under the condition that confidentiality is maintained. The contents of this document are not to be used in any other clinical study, or disclosed to any other person or entity without the prior written permission of the Sponsor. The foregoing shall not apply to disclosure required by any regulations; however, prompt notice will be given to the Sponsor prior to any such disclosure.

The Sponsor plans to disclose details of the study protocol on a web-based, publicly available, clinical trial register database (e.g. ClinicalTrials.gov).

1. REGULATORY CONSIDERATIONS
   1. Clinical Trial Authorisation

As confirmed by the MHRA, a Clinical Trial Authorisation is not required for this study.

- 1. Visits by Regulatory Authorities

With the exception of statutory regulatory authority inspections, the Sponsor will be consulted in the event of inspection of the clinical sites by an outside authority before the Inspectors are permitted access to any of the study records or study areas.

1. ETHICAL CONSIDERATIONS
   1. Ethics Committee Approval

This study will be considered by an EC.

The study will not start until the EC have given their written approval of the protocol and ICF.

If there are any changes to the approved protocol (with the exception of emergency modifications required for subject safety), a protocol amendment will be issued by Covance and agreed by the Sponsor. The EC must give their written approval of any substantial amendments likely to affect the safety of the subjects or the conduct of the study. The EC must be notified of all other changes.

Covance will maintain records of all correspondence with the EC.

- 1. Addressing Ethical Issues and the Ethical Conduct of the Study

The Sponsor does not wish the subjects’ compensation to incentivise subjects to smoke. The stipends have been calculated independently by the clinic using the usual rates for this type of clinical study. The stipend therefore offers fair compensation for the inconvenience and effort required of the subjects. Inclusion and exclusion criteria have been carefully tailored so as not to recruit any smoker who is considering quitting smoking in the next 12 months into the continue-to-smoke/THP population (Arms A and B). This study does not force smokers to smoke or use the THP, nor does it require any subject to continue to participate in the study should they not wish to do so. The ICF to be signed by each subject will specify the requirements of the study and the risks associated with participation in the study. During the exposure period there are no specified smoking times, the smoker chooses if and when they smoke or use the THP, as applicable.

The age of each subject will be verified using appropriate identification documentation prior to inclusion in the study to ensure that all subjects comply with the age restrictions specified in this protocol.

- 1. Subject Information and Informed Consent

Prior to commencement of the study, the Investigator, or their appropriately qualified designee, will ensure that the subject is given full and adequate oral and written information in non-technical terms about the nature, purpose, potential risks and possible benefits for study subjects. A possible benefit is the comprehensive medical screening. Subjects will be instructed that they are free to obtain further information from the Investigator or their appropriately qualified designee and that they are free to withdraw their consent and to discontinue their participation in the study at any time without giving a reason.

The subject should be given time for consideration of all the information and have the opportunity to ask questions. Following discussion of the study with the Investigator or their appropriately qualified designee at the study site, subjects will be invited to read, sign and date the ICF summarising the discussion prior to conducting any procedure related to the study, in the presence of a physician or appropriately qualified designee. This indicates that they are freely giving their informed consent to participate in the study. The original of the signed ICF must be stored by the Investigator and a copy of the signed ICF will be given to the subject.

The ICF is written in non-technical language and will be provided as a separate document.

All subject names will be filed confidentially by the Investigator. Subjects will be identified in documentation and throughout evaluation by the number allotted to them during the study. The subjects will be told that their personal data and all study findings will be stored on computer and handled in the strictest confidence. Subject personal data may only be disclosed to third parties as permitted by the ICF signed by the subject, unless permitted or required by law.

- 1. Declaration of Helsinki

This study will be conducted in accordance with consensus ethical principles derived from the Declaration of Helsinki.

- 1. Good Clinical Practice (GCP)

The study will be conducted in accordance with the following:

- ICH E6 R2: Good Clinical Practice: Consolidated guideline CPMP/ICH/135/95 (July 1996), adopted in the EU by CPMP
- European Commission Directive 2001/20/EC (April 2001)
- European Commission Directive 2003/94/EC (October 2003)
- European Commission Directive 2005/28/EC (April 2005)

The Investigator will be responsible for the overall conduct at the study site and adherence to the requirements of the ICH guidelines and all other applicable local regulations.

- 1. Adherence to the Protocol

The clinical site will adopt all reasonable measures to record data in accordance with the protocol. Under practical working conditions, however, some minor variations may occur due to circumstances beyond the control of the clinical site. All such deviations will be documented in the study records, together with the reason for their occurrence; where appropriate, deviations will be detailed in the clinical study report.

The Covance Quality Assurance group will conduct an inspection of the study procedures at any of the sites at the request of the Sponsor. The findings will be reported to the relevant Project Lead at the site.

- 1. Protocol Amendments

Substantial protocol amendments will be submitted to the EC. Approval must be obtained from the EC before implementation of any changes, except for changes necessary to eliminate an immediate hazard to subjects or any non-substantial changes, as defined by regulatory requirements.

1. DATA MANAGEMENT METHODS
   1. Data Quality Assurance

Quality control and quality assurance will be performed according to clinical sites standard operating procedures or per Sponsor request and as applicable according to the contract between clinical sites and the Sponsor.

The study may be audited or reviewed by an independent quality assurance department, EC and/or regulatory authority at any time. The Investigator or their appropriately qualified designee will be given notice before an audit occurs. The study site will permit study-related monitoring, audits, EC review, and regulatory inspections by providing direct access to source data/documents.

Measures will be undertaken to protect the confidentiality of records that could identify subjects, respecting the privacy and confidentiality rules in accordance with applicable regulatory requirements.

- 1. Case Report Form

An electronic data capture system will be used in this study. Data will be captured in source paper data documents (workbooks) and then entered into the electronic data capture system by staff at the clinical site. Following data entry, the eCRF pages and the data entry will undergo quality control checks in accordance with Covance procedures. Any discrepancies will be resolved in the database.

Following all data validation steps, the Investigator or designee will electronically sign the completed electronic data prior to database lock.

- 1. Monitoring

The Sponsor will designate a Study Monitor who will be responsible for monitoring this clinical study. The Study Monitor will monitor the study conduct, eCRF and source documentation completion and retention, and accurate study product accountability. To this end, the Study Monitor will visit the study site at suitable intervals and be in frequent contact through verbal and written communication. It is essential that the Study Monitor has access to all documents, related to the study and the individual subjects, at any time these are requested. In turn, the Study Monitor will adhere to all requirements for subject confidentiality as outlined in the ICF. The Investigator and Investigator’s staff will be expected to cooperate with the Study Monitor, to be available during a portion of the monitoring visit to answer questions, and to provide any missing information.

- 1. Data Storage and Archiving

All primary data generated by the clinical sites or copies thereof (e.g. laboratory records, data sheets, correspondence, photographs, computer records), which are a result of the original observations and activities of the clinical study and are necessary for the reconstruction and evaluation of the study report, will be retained in the clinical sites archive for a period of 5 years after issue of the final clinical study report. At this time the Sponsor will be contacted to determine whether the data should be returned, retained or destroyed on their behalf. The Sponsor will be notified of the financial implications of each of these options at the time. It is the Sponsor’s responsibility to consider any regulatory implications of these options. No data will be destroyed without the agreement of the Sponsor.

Specimens requiring frozen storage are specifically excluded from the above. These will be retained for as long as the quality of the material permits evaluation but for no longer than 6 months after completion of the study. The Sponsor will be notified of the intent to destroy samples and any financial implications before specimens are destroyed on their behalf.

1. REFERENCES

1. International Agency for Research on Cancer. *Tobacco Control: Reversal of Risk after Quitting Smoking*. **11,** (International Agency for Research on Cancer, 2007).

2. World Health Organization. WHO report on the global tobacco epidemic 2011: Warning about the dangers of tobacco. in (World Health Organization, 2011).

3. National Center for Chronic Disease Prevention and Health Promotion (US) Office on Smoking and Health. *The Health Consequences of Smoking—50 Years of Progress: A Report of the Surgeon General*. (Centers for Disease Control and Prevention (US), 2014).

4. Doll, R., Peto, R., Wheatley, K., Gray, R. & Sutherland, I. Mortality in relation to smoking: 40 years’ observations on male British doctors. *BMJ* **309,** 901–911 (1994).

5. Institute of Medicine. *Clearing the Smoke: Assessing the Science Base for Tobacco Harm Reduction*. (The National Academies Press, 2001). doi:10.17226/10029

6. U.S. Department of Health and Human Services. *How Tobacco Smoke Causes Disease: The Biology and Behavioral Basis for Smoking-Attributable Disease: A Report of the General Surgeon*. (U.S. Department of Health and Human Services Centers for Disease Control and Prevention National Center for Chronic Disease Prevention and Health Promotion Office on Smoking and Health, 2010).

7. Royal College of Physicians. *Nicotine without smoke: Tobacco harm reduction*. (2016).

8. Perfetti, T. & Rodgman, A. The Complexity of Tobacco and Tobacco Smoke. *Beitr. Zur Tab. Contrib. Tob. Res.* **24,** (2014).

9. Farsalinos, K. E. & Polosa, R. Safety evaluation and risk assessment of electronic cigarettes as tobacco cigarette substitutes: a systematic review. *Ther. Adv. Drug Saf.* **5,** 67–86 (2014).

10. Polosa, R. & Benowitz, N. L. Treatment of nicotine addiction: present therapeutic options and pipeline developments. *Trends Pharmacol. Sci.* **32,** 281–289 (2011).

11. Lunell, E., Molander, L., Ekberg, K. & Wahren, J. Site of nicotine absorption from a vapour inhaler - comparison with cigarette smoking. *Eur. J. Clin. Pharmacol.* **55,** 737–741 (2000).

12. Digard, H., Proctor, C., Kulasekaran, A., Malmqvist, U. & Richter, A. Determination of Nicotine Absorption from Multiple Tobacco Products and Nicotine Gum. *Nicotine Tob. Res.* **15,** 255–261 (2013).

13. British American Tobacco (Investments) Ltd. Glo Investigator’s Brochure. 1st Edition. (2016).

14. Frost-Pineda, K. *et al.* Short-term clinical exposure evaluation of a third-generation electrically heated cigarette smoking system (EHCSS) in adult smokers. *Regul. Toxicol. Pharmacol.* **52,** 104–110 (2008).

15. Martin Leroy, C. *et al.* Reduced exposure evaluation of an Electrically Heated Cigarette Smoking System. Part 7: A one-month, randomized, ambulatory, controlled clinical study in Poland. *Regul. Toxicol. Pharmacol.* **64,** S74–S84 (2012).

16. Sakaguchi, C., Kakehi, A., Minami, N., Kikuchi, A. & Futamura, Y. Exposure evaluation of adult male Japanese smokers switched to a heated cigarette in a controlled clinical setting. *Regul. Toxicol. Pharmacol.* **69,** 338–347 (2014).

17. Lüdicke, F., Haziza, C., Weitkunat, R. & Magnette, J. Evaluation of Biomarkers of Exposure in Smokers Switching to a Carbon-Heated Tobacco Product: A Controlled, Randomized, Open-Label 5-Day Exposure Study. *Nicotine Tob. Res.* **18,** 1606–1613 (2016).

18. World Health Organization. *WHO Study Group on Tobacco Product Regulation: Report on the Scientific Basis of Tobacco Product Regulation: Fifth Report of a WHO Study Group*. (World Health Organization, 2015).

19. S. Erhan Deveci, Figen Deveci, Yasemin Acik & A Tevfik Ozan. The measurement of exhaled carbon monoxide in healthy smokers and non-smokers. *Respir. Med.* **98,** 551–556 (2004).

20. Roux, A. *et al.* Early improvement in peripheral vascular tone following smoking cessation using nicotine replacement therapy: aortic wave reflection analysis. *Cardiology* **117,** 37–43 (2010).

21. Miller, M. R. *et al.* Standardisation of Spirometry. *Eur. Respir. J.* **26,** 319–338 (2005).

22. Quanjer, P. H. *et al.* Multi-Ethnic Reference Values for Spirometry for the 3–95 Year Age Range: The Global Lung Function 2012 Equations. *Eur. Respir. J.* **40,** 1324–1343 (2012).

23. Holland, A. E. *et al.* An official European Respiratory Society/American Thoracic Society technical standard: field walking tests in chronic respiratory disease. *Eur. Respir. J.* **44,** 1428–1446 (2014).

24. Fagerstrom, K., Russ, C., Yu, C.-R., Yunis, C. & Foulds, J. The Fagerstrom Test for Nicotine Dependence as a Predictor of Smoking Abstinence: A Pooled Analysis of Varenicline Clinical Trial Data. *Nicotine Tob. Res.* **14,** 1467–1473 (2012).

25. Olufade, A. O. *et al.* Development of the Smoking Cessation Quality of Life questionnaire. *Clin. Ther.* **21,** 2113–2130 (1999).

26. Food and Drug Administration. Guidance for Industry: Modified Risk Tobacco Product Applications. (2012).

27. Food and Drug Administration. Guidance for Industry and FDA Premarket and Design Control Reviewers: Medical Device Use: Safety: Incorporating Human Factors Engineering into Risk Management. (1999).

28. O’Brien, P. C. & Fleming, T. R. A multiple testing procedure for clinical trials. *Biometrics* **35,** 549–556 (1979).

1. APPENDICES

Appendix 15.1: Schedule of Assessments

|  | **Screening** | **Visit 1**  **(Baseline)** | **Visit 2 ^j^** | **Visit 3 ^j^** | **Visit 4** | **Visit 5 ^j,m^** | **Visit 6 ^j,m^** | **Visit 7** | **Visit 8 ^j,m^** | **Visit 9 ^j,m^** | **Visit 10 ^j^** | **Visit 11 ^j,m^** | **Visit 12 ^j,m^** | **Visit 13** | **Follow-up** |
| --- | --- | --- | --- | --- | --- | --- | --- | --- | --- | --- | --- | --- | --- | --- | --- |
| **Day** | **-28 to -1** | **1** | **30**  **(+/- 3)** | **60**  **(+/- 3)** | **90**  **(+/- 3)** | **120**  **(+/- 14)** | **150**  **(+/- 14)** | **180**  **(+/- 14)** | **210**  **(+/- 14)** | **240**  **(+/- 14)** | **270**  **(+/- 14)** | **300**  **(+/- 14)** | **330**  **(+/- 14)** | **360**  **(+/- 14)** | **≤388** |
| Informed consent | X |  |  |  |  |  |  |  |  |  |  |  |  |  |  |
| Inclusion/exclusion | X | X |  |  |  |  |  |  |  |  |  |  |  |  |  |
| Randomisation/ enrolment |  | X |  |  |  |  |  |  |  |  |  |  |  |  |  |
| Socio-demographic data | X |  |  |  |  |  |  |  |  |  |  |  |  |  |  |
| Urine cotinine test | X | X |  |  |  |  |  |  |  |  |  |  |  |  |  |
| Serology | X |  |  |  |  |  |  |  |  |  |  |  |  |  |  |
| Product trial^a^ |  | X |  |  |  |  |  |  |  |  |  |  |  |  |  |
| Distribution/collection of products^b^ |  |  | X | X | X | X | X | X | X | X | X | X | X |  |  |
| CO eligibility | X^l^ |  |  |  |  |  |  |  |  |  |  |  |  |  |  |
| Urine drugs of abuse | X | X | X | X | X | X | X | X | X | X | X | X | X | X |  |
| Alcohol breath test | X | X | X | X | X | X | X | X | X | X | X | X | X | X |  |
| Pregnancy test^c^ | X | X | X | X | X | X | X | X | X | X | X | X | X | X | X |
| FSH test | X |  |  |  |  |  |  |  |  |  |  |  |  |  |  |
| Height | X |  |  |  |  |  |  |  |  |  |  |  |  |  |  |
| Weight | X |  |  |  |  |  |  |  |  |  |  |  |  |  |  |
| BMI | X |  |  |  |  |  |  |  |  |  |  |  |  |  |  |
| Medical history | X | X |  |  |  |  |  |  |  |  |  |  |  |  |  |
| Physical examination | X | X^d^ | X^d^ | X^d^ | X^d^ |  |  | X^d^ |  |  | X^d^ |  |  | X^d^ | X^d^ |
| Vital signs (full) | X | X |  |  | X |  |  | X |  |  | X |  |  | X | X |
| 12-Lead ECG | X | X |  |  | X |  |  | X |  |  | X |  |  | X | X |
| Clinical laboratory evaluations^e^ | X | X |  |  | X |  |  | X |  |  | X |  |  | X | X |
| Lung function tests | X^f^ | X | X | X | X |  |  | X |  |  | X |  |  | X |  |
| AE/SAE recording | X | | | | | | | | | | | | | |  |
| Concomitant medication | X | | | | | | | | | | | | | |  |
| **Exhaled Breath Endpoint Assessments^g^** | | | | | | | | | | | | | | | |
| CO |  | X | X | X | X | X | X | X | X | X | X | X | X | X |  |
| NO |  | X | X | X | X |  |  | X |  |  | X |  |  | X |  |
| **24-hour Urine Endpoint Assessments^h^** | | | | | | | | | | | | | | | |
| 8-Epi-PGF_2α_ |  | X | X | X | X |  |  | X |  |  | X |  |  | X |  |
| 4-HNE |  | X | X | X | X |  |  | X |  |  | X |  |  | X |  |
| 11-dTx |  | X | X | X | X |  |  | X |  |  | X |  |  | X |  |
| Total NNAL |  | X | X | X | X |  |  | X |  |  | X |  |  | X |  |
| TNeq |  | X | X | X | X |  |  | X |  |  | X |  |  | X |  |
| Total NNN |  | X | X | X | X |  |  | X |  |  | X |  |  | X |  |
| 3-HPMA |  | X | X | X | X |  |  | X |  |  | X |  |  | X |  |
| HMPMA |  | X | X | X | X |  |  | X |  |  | X |  |  | X |  |
| S-PMA |  | X | X | X | X |  |  | X |  |  | X |  |  | X |  |
| MHBMA |  | X | X | X | X |  |  | X |  |  | X |  |  | X |  |
| CEMA |  | X | X | X | X |  |  | X |  |  | X |  |  | X |  |
| 1-OHP |  | X | X | X | X |  |  | X |  |  | X |  |  | X |  |
| HEMA |  | X | X | X | X |  |  | X |  |  | X |  |  | X |  |
| Creatinine |  | X | X | X | X |  |  | X |  |  | X |  |  | X |  |
| 4-ABP |  | X | X | X | X |  |  | X |  |  | X |  |  | X |  |
| 2-AN |  | X | X | X | X |  |  | X |  |  | X |  |  | X |  |
| o-Tol |  | X | X | X | X |  |  | X |  |  | X |  |  | X |  |
| **Serum / Plasma / Whole Blood Endpoint Assessments** | | | | | | | | | | | | | | | |
| WBC count |  | X | X | X | X |  |  | X |  |  | X |  |  | X |  |
| MCP-1 |  | X |  |  | X |  |  | X |  |  | X |  |  | X |  |
| s-ICAM1 |  | X |  |  | X |  |  | X |  |  | X |  |  | X |  |
| Fib |  | X |  |  | X |  |  | X |  |  | X |  |  | X |  |
| hsCRP |  | X |  |  | X |  |  | X |  |  | X |  |  | X |  |
| HMCys |  | X |  |  | X |  |  | X |  |  | X |  |  | X |  |
| Gluc |  | X |  |  | X |  |  | X |  |  | X |  |  | X |  |
| PAI-1 |  | X |  |  | X |  |  | X |  |  | X |  |  | X |  |
| tPA |  | X |  |  | X |  |  | X |  |  | X |  |  | X |  |
| SELE |  | X |  |  | X |  |  | X |  |  | X |  |  | X |  |
| ET-1 |  | X |  |  | X |  |  | X |  |  | X |  |  | X |  |
| 3-NTyr |  | X |  |  | X |  |  | X |  |  | X |  |  | X |  |
| HDL/LDL/ Chol_Total_/ triglycerides |  | X | X | X | X |  |  | X |  |  | X |  |  | X |  |
| CEVal (HB adduct) |  | X | X | X | X |  |  | X |  |  | X |  |  | X |  |
| **Physiological Endpoint Assessments ^i^** | | | | | | | | | | | | | | | |
| AIx |  | X | X | X | X |  |  | X |  |  | X |  |  | X |  |
| Body weight and waist circumference |  | X |  |  | X |  |  | X |  |  | X |  |  | X |  |
| Carotid/femoral pulse wave velocity |  | X | X | X | X |  |  | X |  |  | X |  |  | X |  |
| 6-minute walking test |  | X |  |  | X |  |  |  |  |  |  |  |  | X |  |
| Finger plethysmography |  | X |  |  | X |  |  | X |  |  | X |  |  | X |  |
| **Questionnaires Assessments** | | | | | | | | | | | | | | | |
| Tobacco use history | X |  |  |  |  |  |  |  |  |  |  |  |  |  |  |
| FTCD^j^ | X | X |  |  |  |  |  |  |  |  |  |  |  |  |  |
| Product Satisfaction^a^ |  |  |  |  | X |  |  | X |  |  | X |  |  | X |  |
| Smoking Cessation Quality of Life^k^ |  | X |  |  | X |  |  | X |  |  | X |  |  | X |  |
| Cough and Shortness of Breath VAS |  | X |  |  | X |  |  | X |  |  | X |  |  | X |  |
| Self-reported product use (eDiary/paper diary) |  | X | | | | | | | | | | | | |  |
| **Exploratory Endpoint Assessments** | | | | | | | | | | | | | | | |
| Body fat analysis |  | X |  |  | X |  |  | X |  |  | X |  |  | X |  |
| BH2/BH4 |  | X | X | X | X |  |  | X |  |  |  |  |  | X |  |
| Transcriptomics (Nasal) |  | X |  |  | X |  |  | X |  |  |  |  |  | X |  |
| Transcriptomics (WBC) |  | X |  |  | X |  |  | X |  |  |  |  |  | X |  |
| Serum NMR lipoprotein |  | X | X | X | X |  |  | X |  |  |  |  |  | X |  |
| Serum metabolomics |  | X | X | X | X |  |  | X |  |  |  |  |  | X |  |

Abbreviations: 1-OHP = 1-hydroxypyrene; 2-AN = 2-aminonaphthalene; 3-HPMA = 3-hydroxypropylmercapturic acid; 3-NTyr = 3-nitrotyrosine; 4-ABP = 4-aminobiphenyl; 4-HNE = 4-hydroxy-nonenal + metabolites; 8-Epi-PGF_2α_ = 8-Epi-Prostaglandin F_2α_ Type III; 11-dTx B2 = 11-dehydrothromboxane B2; AE = adverse event; AIx = Augmentation Index; BH2/BH4 = dihydrobiopterin/tetrahydrobiopterin ratio; BMI = body mass index; CEMA = 2-cyanoethylmercapturic acid; CEVal (Hb adduct) = N-(2-cyanoethyl)valine (haemoglobin adduct); CholTotal = total cholesterol; CO = carbon monoxide; ECG = electrocardiogram; ET-1 = endothelin-1; Fib = fibrinogen; FSH = follicle-stimulating hormone; FTCD = Fagerström test for cigarette dependence; Gluc = glucose; HDL = high-density lipoprotein; HEMA = 2-hydroxyethylmercapturic acid; HMCys = homocysteine; HMPMA = 3-hydroxy-1-methylpropylmercapturic acid; hsCRP = high-sensitivity C-reactive protein; LDL = low-density lipoprotein; MCP-1 = monocyte chemotactic protein 1; MHBMA = monohydroxybutenyl-mercapturic acid; NMR = nuclear magnetic resonance; NNAL = 4-(methylnitrosamino)-1-(3-pyridyl)-1-butanol; NNN = N-nitrosonornicotine; NO = nitric oxide; o-Tol = ortho-toluidine; PAI-1 = plasminogen activator inhibitor-1; SAE = serious adverse event; SELE – E-selectin; s-ICAM1 = soluble intercellular adhesion molecule-1; S-PMA = S-phenylmercapturic acid; TNeq = total nicotine equivalents (nicotine, cotinine, 3-hydroxycotinine and their glucuronide conjugates); tPA = tissue plasminogen activator; WBC = white blood cell.

^a^ Arms A and B only.

^b^ Arm B only.

^c^ Female subjects of childbearing potential. Performed in serum at Screening and in urine at all other times.

^d^ Symptom driven physical examination.

^e^ Includes serum biochemistry, haematology, and urinalysis.

^f^ Spirometry conducted pre and post-bronchodilator.

^g^ Subjects will not be allowed to use a study product within the 30 minutes prior to CO assessments.

^h^ To begin the day before each study visit.

^i^ Blood pressure and lung function tests (spirometry) as physiological endpoints are presented with safety parameters.

^j^ Arms A, B, and D only.

^k^ Arms B and D only.

^l^ Subjects may be rescreened to confirm CO levels if they have a low CO level at screening

^m^ Visit should occur between 1630 and 2100 hours
