# Supplementary material for: Evaluating the effects of switching from cigarette smoking to using a heated tobacco product on health effect indicators in healthy subjects: study protocol for a randomized controlled trial
Source: Intern Emerg Med. 2019 May 2;14(6):885–98. doi: 10.1007/s11739-019-02090-8 (PMC6722146; doi:10.1007/s11739-019-02090-8)
Supplement: Supplementary file 2 — Supplementary file2 (DOCX 98 kb) [file 11739_2019_2090_MOESM2_ESM.docx]

(To be printed on hospital/institution headed paper)

1. **PARTICIPANT Information Sheet**

| **Study Title:** A Randomised, Controlled Study to Evaluate the Effects of Switching from Cigarette Smoking to using a Tobacco Heating Product on Health Effect Indicators in Healthy Subjects. |
| --- |
| **Protocol No**.: BAT3117011 |
| **Name and Address of Sponsor:**  British American Tobacco (Investments) Ltd.  R&D Centre Regents Park Road Southampton Hampshire SO15 8TL, UK. |
| **Principal Investigator Name**: <full name> |
| **Institution:** <name and full address> |
| **IRAS No.: 230414** |

**It is important that you give a true and complete medical history. You must be honest about your past and present usage of medications. Giving information that is not true could be very harmful to your health. If you give false information, you may be dismissed from the study.**

You are being asked to take part in a research study sponsored by British American Tobacco (Investments) Limited (Sponsor). The clinical research site is being paid by the Sponsor to conduct this study. You should read this form before you decide if you want to take part in the study. This form will tell you about the study.

The Study Doctor or study staff can explain words or information that you do not understand. Ask the study staff as many questions as needed for you to decide if you want to take part in the study.

Research studies are voluntary and include only those who wish to take part. If you decide to take part in this study, you must sign your name at the end of the form and date it. You cannot take part in this study until you sign and date this form.

Once you have signed and dated this form, in the presence of the study staff, you will receive one original which you will take home. When making the decision to participate in the study, it is important that you accept its course, its purpose, the planned procedures, and that you are ready to participate until the end of the study.

Prior to the start of this study, an Independent Ethics Committee (IEC) reviewed the scientific and ethical aspects of the study and has given their approval.

**Part 1**

### **Introduction**

Cigarette smoking has been identified as a contributing factor to numerous illnesses including lung cancer, chronic obstructive lung disease and heart disease. The health risks associated with cigarette smoking are known to be due to chemicals toxicants in cigarette smoke, which can lead to changes in the body, causing disease. Nicotine is primarily responsible for the addictive properties of cigarette smoking.

The Sponsor of this study is British American Tobacco (Investments) Limited, a manufacturer of tobacco products. The Sponsor is developing an alternative approach to conventional (normal) cigarettes, by developing new products which may have the potential to reduce some of the risks of tobacco-related diseases.

You are being invited to take part in a clinical research study to test the use of a “Tobacco Heating Product” (THP). In this document, the THP to be used in this study will be referred to as the “investigational product”.

Participants in this study are required to understand the risks associated with smoking. The only known way to reduce the risks of smoking-related diseases described above is to stop smoking.

### **Purpose of this Study**

This study was designed for research purposes to collect data on a newly developed investigational product in adult smokers. The main purposes of this study are:

- To measure 'health effect indicators'. This will determine how your body responds to the THP and how this effects your health.
- To determine if the level of chemical toxicants that the body is exposed is lower following the use of the THP compared to the levels of exposure in people who have continued to smoke conventional cigarettes and/or roll your own cigarettes, stopped smoking or who have never smoked.
- To obtain safety information related to the use of the THP.

Biomarkers are molecules that indicate normal or abnormal process taking place in your body that can be detected and measured in blood or urine. In this study, biomarkers will be measured by testing your blood and your urine which will be collected over a 24-hour period.

Reductions in the level of exposure to chemical toxicants are measured in your biomarkers. Biomarkers of exposure are substances measured in your body as the result of consumption of another substance (such as cigarette smoke).

As part of the study, we will also be taking two samples which will be used for an analysis technique known as “transcriptomics”. The purpose of this analysis is to look at your RNA (genetic information) in more detail. These samples are as follows: blood samples to look at the genetic information in your white blood cells, and a nasal sample. The nasal sample will be collected by a painless procedure where a swab will be placed into your nasal passage to collect a sample of nasal epithelial cells for analysis. Please refer to Section 5 for further details on when these samples will be taken.

### **Do I have to take part?**

If you choose to take part in the study you will do so by your own choice and your own free will. No one can force you to be in the study. If you enter the study, no one can force you to stay in the study. If you choose not to be in the study or if you leave the study early, there will be no penalty. If you leave, are removed from the study or the study is wholly or partly cancelled for any reason, your financial compensation will be prorated to the amount of the study that you complete. You will not lose any rights that you are entitled to as a research participant.

### **What will happen to me if I take part?**

**Test Methods**

THPs have been recently developed as alternatives to conventional cigarettes.

Tobacco Heating Products (THPs) consist of a heating device which heats rather than burns a stick of tobacco (typically to temperatures around 240 to 350ºC). This stick of tobacco is similar in appearance to a cigarette. Based on the Sponsor’s own assessments of the chemical toxicants found in the emissions from a THP developed by the Sponsor, it is thought there are significantly fewer chemical toxicants in those emissions when compared to toxicants found in cigarette smoke.

**Study Design**

This study is a randomised, open label study comparing 3 different populations of participants.

The 3 populations of participants that will be investigated in the study are as follows:

- Participants who will continue to smoke (CTS) conventional cigarettes and/or roll your own cigarettes or use of the investigational product.
- Participants who are intending to quit smoking.
- Participants who have never smoked a cigarette.

Participants, who qualify for the smoking part of this study, will be assigned by chance to one of the following:

- Product 1 – Continue to smoke your own choice of cigarette brand and/or roll your own
- Product 2 – glo THP device with Neostik (British American Tobacco)

You will have an approximate 24.5% chance of being aked to continue to smoke your own brand cigarettes and a 75.5% chance of being asked to smoke the THP.

If you are assigned to receive Product 2 you will be provided with the necessary investigational product. If you are assigned to continue to smoke your own choice of cigarette brand you will continue to pay for your own cigarettes.

If you are a participant who intends to quit smoking and qualify for the study, then you will only be assigned to the intending to quit smoking arm.

If you are a participant who has never smoked and qualify for the study, then you will only be assigned to the never smoked arm.

In total this study will enroll approximately 495 participants.

There will be a total of 4 study arms (to be labelled A, B, D and E) as follows:

| **Study Arm** | **Recruitment population** | **Randomised Product use** |
| --- | --- | --- |
| A | THP/CTS population | CTS conventional cigarettes and/or roll your own cigarettes  (Product 1) |
| B |  | Product 2 |
| D | Intending to quit population | None |
| E | Never smoked population | None |

Abbreviations: CTS = continue to smoke; THP = Tobacco Heating Product

For study Arms A, B and D, participants will be asked to continue smoking their usual brand of cigarette until randomisation (Arms A-B) or enrolment (Arm D) on Day 1.

Participants in Arms A and B will only use their assigned product from randomisation (Day 1) until Visit 13 (Day 360). During this time, you can use the product assigned to you as you wish. You will be informed of any limitations prior to starting the study.

Participants in Arm D will only use study-authorised nicotine replacement therapies provided by the Study Doctor following enrolment on Day 1 until Day 360.

Participants in Arm E will not use any tobacco or nicotine products/ nicotine replacement therapies/ smoke cigarettes during the study.

For all Arms, your participation in the study will last up to 416 days (approximately 1 year and 7 weeks) from the Screening Visit until the Follow-up Visit. You will not be required to stay overnight at the clinical research site but you will be required to visit the clinical research site a total of 15 times over the duration of the study. Subjects in Arms A, B and D will attend clinic visits at Screening, Day 1, Days 30, 60, and 90 (+/-3 days), Days 120, 150, 180, 210, 240, 270, 300, 330, and 360 (+/- 2 weeks), and at Follow-up (within 28 days following last visit). Subjects in Arm E will only attend clinic visits at Screening, Day 1, Day 90 (+/-3 days), Days 180 and 360 (+/- 2 weeks), and at Follow-up (within 28 days following last visit).

**Tobacco Heating Product/ Continue to Smoke Population (Arms A and B)**

For participants in the THP/CTS population, following baseline assessments but prior to randomization, you will be offered a product test. Participants will be given the opportunity to trial the product for a limited amount of time.

Following product test on Day 1 and prior to check-out, participants willing to take part in the study will be randomised to Arms A or B. Participants assigned to Arm B will be provided with the investigational product and directions on how to use this correctly.

**Intending to Quit Population (Arm D)**

Participants in the intending to quit population will be expected to refrain from nicotine and tobacco product use from the time of their enrolment into Arm D. Participants will receive support as needed for the duration of the study (explained further in section 11 below).

**Never Smoked Population (Arm E)**

Participants in the Never Smoked Population (Arm E) will be instructed not to use tobacco or nicotine containing products for the duration of the study.

### **Study Visits**

#### 5.1 Screening

The Screening Period is within 28 days before Visit 1 and will consist of a visit to the research site for additional screening tests. The reason for the Screening Visit is to make sure that you are in good health and are suitable to take part in the study.

If you pass the screening assessments you may be invited to take part in the Study. If you are invited to check in to the clinical research site there is a possibility that you will be selected to be a standby participant. Standby participants who are not required will be discharged from the clinical research site.

During this visit, the following procedures will be performed:

- Provide confirmation of your age to enable the Study Doctor to verify your age. To enter this study you must be aged at least 23 years old.
- Review of inclusion/ exclusion criteria.
- Read and sign this form if you wish to participate.
- Provide your medical and medication history including your name, age, sex, race, and ethnicity, address national insurance number and telephone number.
- An ECG - electrocardiogram recording (a test that measures and records the electrical activity of your heart).
- A full physical examination.
- Vital signs (pulse rate, blood pressures, body temperature and respiratory rate) and your height and body weight will be measured.
- A lung function test. At Screening this test will be performed with and without a bronchodilator. A bronchodilator is a type of medicine that makes breathing easier by relaxing the muscles in your lungs and widening the airways.
- A pregnancy blood test (female participants only).
- A blood test to confirm menopausal status (female participants only).
- A urine test for drugs of abuse and an alcohol breath test.
- A urine test for cotinine (cotinine is a breakdown product of nicotine) and a carbon monoxide breath test. Both of these tests provide evidence that you are a smoker (or non-smoker for participants in Arm E).
- A questionnaire which will record your tobacco use history and your nicotine dependence (participants in Arm E will not have to complete the nicotine dependence questionnaire).
- You will be asked if you are planning to quit smoking in the next 12 months.
- Blood and urine samples will be collected. Your blood and urine sample will be used for routine laboratory tests. Your blood will also be used to test for HIV, hepatitis B and C. If your result for one of these tests shows positive, your sample will be sent to an external laboratory for confirmation that the result is correct as false positives sometimes occur i.e. the test shows a positive result but you may not be infected.
- Questions about your health and how you are feeling.

After your screening visit to the research site and prior to your Visit 1, if you intend to quit smoking you will be contacted by the research site to discuss your requirements for Nicotine Replacement Therapies or Champix (a prescription medication used to treat nicotine addiction).

#### 5.2 Visit 1 (Baseline)

You will start 24-hour urine collection on the morning of Day -1 (and to include the first void of the day on Day 1). The study coordinator in charge will provide you with a bottle when you need to urinate. Further details will be explained by the study coordinator in charge. At Visit 1, all baseline assessments will be performed. Depending on which population you belong to, the study procedures will differ slightly.

The following procedures will take place when you check-in, to ensure that no changes which may affect your safety and/or the study results have occurred since your Screening Visit:

- Review of inclusion/ exclusion criteria.
- A sample of your urine will be collected and screened for drugs of abuse, and tested for cotinine.
- A urine pregnancy test (female participants only).
- An alcohol and carbon monoxide and nitrous oxide breath test.
- A physical examination.
- Vital signs (pulse rate, blood pressures, body temperature and respiratory rate) and your body weight and waist circumferences.
- ECG recording.
- Any changes in your medical history, including any illnesses and/or use of any medication since your Screening Visit, will be discussed and documented.
- Blood and urine samples for routine safety checks.
- Blood and 24-hour urine samples for the measurement of biomarkers.
- A nasal swab sample for the purpose of collecting your nasal epithelial cells. This sample will be used to analyse your genetic information.
- A blood sample to analyse your white blood cells for genetic information.
- A blood sample to measure your white blood cell count.
- A lung function test.
- Completion of smoking related questionnaires:
  - Nicotine dependence test (Arms A, B and D only).
  - A questionnaire to assess how stopping smoking has affected your quality of life (Arms B and D only).
  - A questionnaire to assess cough and shortness of breath (all Arms)
- Physiological measures:
  - Carotid/ femoral pulse wave velocity measurement (measurement of how stiff your arteries are).
  - 6-minute walking test.
  - Finger plethysomography (measurement of heart rate). This is performed following the attachment of a painless device to your index finger. Participants in Arms A, B, and D will be unable to smoke for at least 2 hours prior to measurements.
  - Determination of augmentation index (measurement of arterial stiffness).
- Self-reported product use (diary). This will be recorded daily between Days 1 and 360 of the study for Arms A, B and D only.
- After all admission tests are confirmed including the female pregnancy test, you will be asked if you want to try the study THP - for the THP/CTS population.
- Assignment to investigational device (Arm B).
- Questions about your health and how you are feeling.
- Arm B will receive a phone call within 7-14 days following Visit 1 reminding you of the support available and to help answer any questions or comments about the investigational product. There will be a 14 day transition period following Visit 1 that will allow you the opportunity to determine if you feel comfortable using the device on a daily basis and are happy to continue using it for the remainder of the Study.
- Arm D will also receive a phone call within a week or two following Visit 1 reminding you of the support available and to discuss your use of any Nicotine Replacement Therapy or Champix. There will be a 14 day transition period following Visit 1 that will allow you the opportunity to continue to use tobacco products should you wish to do so during that period. If you decide you do not wish to quit smoking you will no longer be able to take part in the study.

#### 5.3 Visit 2 - Visit 13

You will return to the clinical research site for non-residential visits every 30 days (Arms A, B and D only) (+/- 3 days for Visit 2 to Visit 4, +/- 2 weeks for Visit 5 to Visit 13).

You will start 24-hour urine collection on the morning of the Day before check-in for Visits 2, 3, 4, 7, 10, and 13 only.

At Visits 2 to 12, participants in Arm B will be asked to return all empty, part used, and unused packs of THP consumables. Participants will then be resupplied with new packs of THP sticks for use over the subsequent 30 day period.

At Visit 13, participants in Arm B will be asked to return all empty, part used, and unused packs of THP consumables, and the study devices, chargers and other accessories supplied to you for this study.

The following procedures will take place at your visit to the clinical research site:

- Distribution and collection of investigational products (Arm B only).
- An alcohol and carbon monoxide and nitrous oxide breath test.
- A urine test for drugs of abuse.
- Urine pregnancy test (female participants only).
- Physical examination (Visit 2, 3, 4, 7, 10, and 13 only).
- ECG recording (Visit 4, 7, 10, and 13 only).
- Lung function test (Visit 2, Visit 3, Visit 4, Visit 7, Visit 10, and Visit 13 only).
- Any changes in your medical history, including any illnesses and/or use of any medication since your last visit, will be discussed and documented.
- Blood and urine samples for routine safety checks (Visit 4, Visit 7, Visit 10 and, Visit 13). You will need to fast for at least 6 hours prior to blood collection. You will be allowed to drink water during this time
- Completion of smoking related questionnaires (Visit 4, Visit 7, Visit 10, and Visit 13).
- Blood samples for the measurement of biomarkers (Visit 2, Visit 3, Visit 4, Visit 7, Visit 10, and Visit 13 only).
- Physiological measures:
  - Carotid/femoral pulse wave velocity measurement (Visit 2, Visit 3, Visit 4, Visit 7, Visit 10, and Visit 13 only).
  - 6-minute walking test (Visit 4 and Visit 13 only).
  - Finger plethysomography. (Visit 4, Visit 7, Visit 10, and Visit 13 only). Participants in Arms A, B, and D will be unable to smoke for at least 2 hours prior to measurements.
  - Determination of augmentation index (Visit 2, Visit 3, Visit 4, Visit 7, Visit 10, and Visit 13 only).
- Vital signs (Visit 4, Visit 7, Visit 10, and Visit 13 only).
- Blood and 24-hour urine sample for the measurement of biomarkers (Visit 2, Visit 3, Visit 4, Visit 7, Visit 10, and Visit 13 only).
- A nasal swab sample for the purpose of collecting your nasal epithelial cells (Visit 4, Visit 7 and Visit 13 only).
- A blood sample to measure your white blood cell count (Visit 2, Visit 3, Visit 4, Visit 7, Visit 10, and Visit 13).
- Completion of smoking related questionnaires:
  - ArmProduct use satisfaction (Arms A and B only). (Visit 4, Visit 7, Visit 10, and Visit 13 only).
  - A questionnaire to assess how stopping smoking has affected your quality of life (Arms B and D only) (Visit 4, Visit 7, Visit 10, and Visit 13 only).
- A questionnaire to assess cough and shortness of breath (all Arms) (Visit 4, Visit 7, Visit 10 and Visit 13 only).
- Self-reported product use (diary). This will be recorded daily prior to each clinic visit for the duration of the study for Arms A, B and D only.
- Questions about your health and how you are feeling.

#### 5.4 Follow-up Visit

The Follow-up Visit will be performed at the clinical research site within 28 days after Check-out at Visit 13. The Study Doctor for the study or their appropriately qualified designee will perform safety assessments.

Provided there are no observed side effects which may require further medical attention, your participation in the study will be considered complete. A further visit may be necessary if deemed necessary by the Study Doctor to ensure that you are in full health prior to discharge.

Participants who withdraw from the study after enrolment will be encouraged to attend a Follow‑up Visit.

The following procedures will take place before you check-out of the research site, to ensure that no changes which may affect your safety and/or the study results have occurred since your Screening Visit and during your time on the study:

- Urine pregnancy test (female participants only).
- Physical examination.
- Blood and urine samples for routine safety checks.
- Vital signs.
- ECG recording.
- Review of any medications you may have taken since your last visit.
- Questions about your health and how you are feeling.

### **Cost and Payment for Taking Part in the Study**

The investigational product (Arm B) and study procedures are provided to you at no charge. You are required to arrange your own transport to and from the clinical research unit.

For Arms A, B and D the research site will pay you £3,500 in total for your full participation in these arms of this study.

For Arm E the research site will pay you £1,500 in total for your full participation in this arm of this study.

The amount you will be paid will depend on how much of the study is completed by you. If you do not complete the study for any reason (including if you are removed from the study or the study is wholly or party cancelled), you will receive a pro-rated amount based on the study days you completed. Study payments may be split and the amount you will receive will depend on your sites payment processes. Please ask the site staff for more information on your payment schedule. It is important that you understand the following:

- No deductions will be withheld from your stipend for tax purposes.
- You are responsible for reporting any payment on your individual tax returns and for payment of any applicable taxes.
- Being in this study does not make you an employee of the Sponsor, research site or any third party vendors.
- You will not receive the full payment for the study if you do not complete it for any reason. This may include leaving the study due to a symptom/sign of illness or side effects, which will be determined by the Study Doctor.
- For Arm B, you will not receive your final payment for the study until you have returned all the investigational products to the Study Doctor as set out in this form.

### **What will I have to do?**

You must:

- Inform us of any medical condition you have been diagnosed with or any current concerns you have about your health.
- Follow all research site rules and instructions of the study staff.
- Follow the study restrictions.
- Report any new symptoms/signs of illness or side effects.
- Report any failure, overheating or other malfunction of the investigational products.
- Give true and complete answers to any questions.
- Comply with the terms of this form.
- Inform us if there are any changes in the information you provided during the screening process, so that the Study Doctor can determine your eligibility at any time during the study.

At the Screening Visit you will be provided with a schedule of the dates and times that you will be asked to attend the clinic.

You will be provided with an emergency contact card at the end of Visit 1. This card contains the study number and emergency contact details and you will be requested to carry the card at all times. The card may be destroyed 4 weeks after completion of the study.

### **HIV Information**

### HIV and hepatitis B and C testing (using a blood sample taken at the Screening visit) is mandatory for your participation in the Study. During the screening process, you will be offered the chance to discuss HIV and hepatitis testing with the Study Doctor. You should be aware that a positive test for HIV or hepatitis can have implications for many aspects of your life, including your health, relationships, work and insurances. The tests should not be undertaken lightly. A negative result for HIV means that antibodies to the virus have not been detected. If you have been at risk within the 3 months prior to the test, there is still a chance you may be infected with HIV, even if the result is negative. The same applies for a negative result for hepatitis B or C.

### If a positive HIV, hepatitis B or hepatitis C result is found, we will discuss this with you and contact your GP, who will arrange appropriate follow-up for you. Please be aware that if a positive HIV, hepatitis B or hepatitis C result is found, you will no longer be eligible to participate in the Study.

### **Requirements and Restrictions**

Smoking History and Study Requirements

For the THP/CTS population, only smokers who regularly smoke 10-30 commercial non‑menthol cigarettes per day and who are willing to switch to a THP will be recruited into this study. You will have smoked regularly for at least 5 years prior to your Screening Visit. Smoking status will be confirmed by a cotinine urine test at the Screening Visit and a carbon monoxide breath test.

For the intending to quit population, only smokers who regularly smoke 10-30 commercial non‑menthol cigarettes per day and who are not intending to use any non-medicinal nicotine products and not intending to use tobacco products will be recruited. This arm will be supported with nicotine replacement therapy/Champix (a prescription medication used to treat nicotine addiction) as appropriate. You will have smoked regularly for at least 5 years prior to your Screening Visit. Smoking status will be confirmed by a cotinine urine test at the Screening Visit and a carbon monoxide breath test.

For the never-smoked population, participants who have never smoked (less than 100 cigarettes in their lifetime and none in the 30 days prior to Screening) will be recruited. This will be confirmed by a cotinine urine test at the Screening Visit and carbon monoxide breath test.

You may decide to quit smoking, and/or to withdraw from the study, at any time. If you do wish to stop smoking, you will be appropriately supported by the Study Doctor and research site team. Please see the further information at section 11 below.

Dietary Restrictions

During your visits to the clinical research site, all your meals will be provided and the consumption of water is permitted.

To avoid effects on the measurement of your biomarkers, you are required to avoid certain foods for 48 hours prior to your admission. You must avoid eating barbecued or chargrilled food. You must avoid eating poppy seeds for 3 days prior to your Screening and prior to checking-in to each Visit and you must refrain from drinking alcohol 24 hours prior to each study visit.

Medication

You must not consume medication as follows:

- From 14 days prior to check-in at Visit 1, until check-out at Visit 13, participants should avoid any medication that could interfere with how your body processes the investigational products. These medications include anti-inflammatory drugs such as aspirin and ibuprofen.
- No prescription or over-the-counter medications (except hormone replacement therapies [HRT] and hormonal contraceptives [e.g. oral, transdermal patch, implant or injection]) should be taken during the course of the study. Herbal medications, vitamins, and supplements should be avoided for 3 days prior to each clinic visit, unless otherwise agreed with the Study Doctor.

Please speak to the Study Doctor before taking any new medication, as this may interfere with the investigational product. The Study Doctor will also explain to you which types of routine medication you can take during the study, such as paracetamol for headaches.

Exercise

You must refrain from strenuous physical activity (beyond your normal activity levels) for 7 days prior to each visit to the clinical research site.

Blood Donation

You must not donate blood during the study and for 12 weeks (male) or 16 weeks (female) after the end of the safety Follow-up Visit.

### **What are the possible disadvantages and risks of taking part?**

**Procedures and Possible Risks or Discomforts**

Procedures will be performed during the study at assigned times. You will be given a schedule of all study procedures. The procedures will be performed to monitor aspects of your health (all Arms), assess your use of the investigational product, and to see how the investigational product is broken down in your body (Arm B only). In the instance that the Study Doctor detects any abnormalities in your health, based on the results of study procedures such as ECG, physical examinations or safety blood draw results, they will discuss this with you immediately. If necessary they might contact your GP directly or provide you with a letter to take to your GP.

If you are experiencing any adverse events of concern whilst attending a study visit, you will remain in the clinic until these have been resolved.

**Risks associated with tobacco products (Not applicable to the Never Smoked Population)**

Tobacco products are addictive and their consumption is associated with real risks of serious diseases. The best way to avoid the risks associated with tobacco products is not to use them at all.

All investigational products are provided to you for your own personal use for the purposes of this study, and you must not provide any investigational product to members of your family, friends or any other person.

All study products must be kept out of sight and reach of children at all times.

**Side effects of using the investigational products (Not applicable to the Never Smoked Population)**

Nicotine and tobacco use can have side effects but as you are already using tobacco products the risks related to the side effects of nicotine through investigational product administration are low. During investigational product use, you are not likely to be exposed to nicotine levels higher than the ones you are usually exposed to during your daily consumption of tobacco products.

The following side effects have been reported for THPs, and you will be monitored for the following possible side effects.

- Headache.
- Dizziness.
- Palpitations.
- Mouth and throat irritation.
- Skin irritation.
- Stomach disturbances.

Inhalation of too much nicotine has been reported to lead to side effects such as feeling faint, nausea or headache. Common observed side effects also include cough, irritation of the mouth or throat, dizziness, nasal congestion, stomach discomfort, hiccups and sickness (vomiting).

There is always a chance that an unexpected or serious side effect may happen. You must report any new symptoms/signs of illness to the Study Doctor or person in charge immediately any time after you have signed this form.

**Tobacco Heated Product (Glo Device)**

When using the THP (Glo Device) you will be provided with a fully charged device. In order to avoid the risk of fire, injury and/or damage to your THP or other property, it is important that you only charge your product with the charging equipment provided to you and in accordance with the instructions for use provided to you with the device. Do not charge a THP device using a car charger.

If the device appears to fail, overheat, or malfunction in any way, please immediately stop using the device and report this to the person in charge at the earliest opportunity. Do not attempt to take the device apart.

If you are provided with THP consumables (tobacco sticks) for use in this study, you must only use these with the THP device provided to you and not with any other THP device. You must not light or attempt to smoke any THP consumables provided to you.

**Risks to an unborn baby or child who is breastfeeding:**

The risks of using nicotine containing products during pregnancy are not known. It is possible that they may cause harm to an unborn baby. This may include death, congenital malformations or other unforeseen health problems for the baby.

If you choose to be sexually active during the study, you must use an acceptable method of birth control. The study staff will discuss with you what an acceptable method of birth control is. A pregnancy may still occur even while using birth control. Not having sex with a person of the opposite sex is the only way to be certain that a pregnancy will not occur, however this should be in line with your usual lifestyle choice.

For participants who are in same sex relationships, contraceptive requirements do not apply.

**Females Participants**

Female participants who are not of child-bearing potential will not be required to use contraception. Women not of child-bearing potential are either:

Permanently sterile (i.e. due to hysterectomy, bilateral salpingectomy, bilateral oophorectomy, or confirmed tubal occlusion)

Or

Postmenopausal (defined as at least 12 months of no period without an alternative medical cause). Postmenopausal status will be confirmed with a FSH blood test at Screening.

If you are able to become pregnant, you must agree to use one of the following methods of birth control in combination with a barrier method of contraception (i.e., a condom with spermicide) from the time of signing this form until the end of the Follow-up Visit:

- Combined oestrogen and progesterone containing oral, intravaginal or transdermal contraception associated with inhibition of ovulation.
- Progestogen-only hormonal contraception, either oral, injected or implanted, associated with inhibition of ovulation.
- Intrauterine device (IUD) (IUD; e.g. Mirena®). Steel or copper IUDs are acceptable.
- Male sterilisation (performed at least 90 days prior to the Screening Visit) with confirmation of surgical success.
- Bilateral tubal ligation (performed at least 90 days prior to Screening Visit).

The potential risks of the investigational products to a baby during breastfeeding are not known. Therefore women who are already pregnant or breastfeeding cannot take part in this study.

**Male Participants**

There is no data about the effect of the investigational products on sperm or its production in the body or about its effects on the development of the foetus. It is essential that your partner does not become pregnant during the study. Men who take part in this study and their female partners must use a condom with spermicide in addition to a second highly effective method of contraception used by their female partner from Visit 1 until the end of the Follow-up Visit.

Men who take part in this Study and whose partner is already pregnant must use condoms during sexual intercourse from Visit 1 until the end of the Follow-up Visit (even if they have had a vasectomy). A man whose partner becomes pregnant during the Study should immediately tell the medical staff at the clinical research site.

You must also refrain from donating your sperm from Visit 1 until the end of the Follow-up Visit.

**Blood Collections**

During the Study, blood will be collected from you on approximately 8 occasions. The total volume of blood taken during the study will be no more than a standard blood donation. If considered necessary, a cannula (small plastic tube inserted into a vein in your forearm using a needle) may be used.

Additional samples may need to be taken for example if we need more information due to you experiencing a side-effect.

Blood sampling and cannula insertion may cause temporary pain, bruising and/or bleeding to your arm and may leave a small scar at the puncture site where multiple blood samples are taken. There is a risk of infection, bleeding and/or bruising at the insertion site.

**Other Risks**

ECG (or telemetry) may result in skin rashes and darkening or lightening of skin at electrode sites.

To collect a sample of your nasal epithelial cells (for genetic analysis), we will need to take a swab from inside your nasal passage. This will be a small swab inserted into your nose which is a painless but unpleasant sensation.

### **Quitting Smoking**

Information will be provided to you regarding the health risks associated with smoking. Advice on smoking cessation will be freely available to you at Screening and during all clinic visits. The advice will be based on the recommendations of the WHO “Evidence based Recommendations on the Treatment of Tobacco Dependence”. If you decide to quit smoking during the study period you will be referred to the appropriate stop smoking services for support.

If you are enrolled into Arm D which is not permitted to use any tobacco or nicotine products after Visit 1, you may experience potential effects of tobacco use abstinence, e.g. irritability, anxiety, nausea, cravings for tobacco, etc. The Study doctor or trained designee will determine a stop smoking strategy with you on Visit 1. Counselling will be offered on site but for additional support, you will be referred to the following services based on where you live:

- **England -** National Health Service (NHS) quitting support website (https://www.nhs.uk/live-well/quit-smoking/nhs-stop-smoking-services-help-you-quit) and the Smokefree helpline (Tel: 0300 123 1044), online advisor support, local stop smoking service, and Smokefree app.
- **Northern Ireland –** Want2Stop quitting support website ([www.want2stop.info](http://www.want2stop.info))
- **Wales –** NHS Wales quitting support website ([www.helpmequit.wales](http://www.helpmequit.wales)) and telephone helpline (0808 085 2219)

You will be provided with a 24-hour site number that you can contact for cessation support if required. If necessary, you will also be provided with nicotine replacement therapy and/ or Champix (a prescription medication used to treat nicotine addiction). The Investigator or their appropriately qualified designee will review your progress and strategy at each clinic visit.

### **What are the possible benefits of taking part?**

You will not receive any health benefits from being in this study. The tests provided may help you learn about your general health. They may also help you discover an unknown medical condition. This study may help doctors, scientists or manufacturers learn things about tobacco and nicotine products that could help others to quit smoking.

You can speak to the Study Doctor at any time to ask for advice on completely stopping using tobacco and nicotine products.

### **Reasons you could be removed from the Study**

This study or your study participation may be stopped without your consent.

The study may be discontinued at the discretion of the Investigator (or designee), Sponsor, or Sponsor’s Medical Monitor if any of the following criteria are met:

- adverse events unknown to date
- increased frequency, severity, and/or duration of known, anticipated, or previously reported AEs (this may also apply to AEs defined at Check‑in as baseline signs and symptoms)
- medical or ethical reasons affecting the continued performance of the study
- difficulties in the recruitment or retention of subjects
- administrative reasons
- cancellation of product development or other business decisions.

Reasons why the Study Doctor can stop your study participation include:

- You are having side effects that require you to stop the investigational product.
- You do not follow the instructions, rules, and restrictions given by the study staff.
- You do not follow the requirements described in this form.
- You do not continue to meet the requirements for the study.
- The Study Doctor decides it is best for your health.
- You become pregnant.

**Part 2**

### **What if relevant new information becomes available?**

You will be told of any significant new safety findings or other information that the Study Doctor is made aware of by the Sponsor that might influence your willingness to continue your participation in this study.

### **What will happen if I don't want to carry on with the Study?**

You are free to leave the study at any time. If you choose to leave the study, you must notify the Study Doctor or study staff. You will be asked to complete the discharge procedures prior to leaving the research site. The procedures will be performed for your safety and well-being.

All data and human biological samples that have been collected prior to you leaving the study will be used and retained for purposes specified in this form. If you do not want your samples to be analysed from the date at which you leave the study you must inform the clinical research site at the time of leaving the study. After withdrawal from the study, no new information will be collected about you.

If you choose to leave the study your study payments may be stopped immediately.

### **What if there is a problem?**

**Contacts in case of emergency and for questions about the study**

If you are feeling unwell or believe you are having a reaction to the product please inform your Study doctor immediately in person or by phone using the 24-hour contact number provided on your patient alert card. Please contact the study staff if you have any questions about this study, its procedures, risks and benefits, or alternative courses of treatment. The names and telephone numbers of the study staff to contact are listed in the table below.

| **Main study doctor** | <NAME> | <CONTACT NUMBER> |
| --- | --- | --- |
| **Other study doctor** | <NAME> | <CONTACT NUMBER> |
| **Study nurse** | <NAME> | <CONTACT NUMBER> |

### **Contact for questions about your rights**

If you have any complaints about any part of the study, the way it is being carried out or any questions about your rights as a study participant, you may contact:

Name of Contact Person: <NAME>

Telephone Number: <NUMBER>

Address: <ADDRESS>

### **Compensation for an Injury Directly Related to your Participation in this Study**

It is important that you tell your Study Doctor, if you feel that you have been injured because of taking part in this study. You can tell the Study Doctor in person or by phone using the 24-hour contact number provided on your patient alert card.

**What happens if the injury or illness is a direct result of the study?**

The Study Doctor will decide if an injury or illness is directly related to the performance of the protocol (study plan) or use of the investigational products.

If your injury or illness is directly related to the performance of the protocol (study plan) or use of the investigational products, the Sponsor and/or the research site will ensure you receive the appropriate treatment either within the National Health System or, if appropriate, from a private healthcare provider. You are not being asked to release or waive any of your legal rights against the Sponsor, the Study Doctor or the Research Site for liability for negligence.

Where applicable, the Sponsor will provide compensation for any injury caused by taking part in this study in accordance with the guidelines for the Association of the British Pharmaceutical Industry (ABPI), this may include any injury caused by an investigational product used as part of the study protocol or any test or procedure you received as part of the study protocol.

The Sponsor holds appropriate clinical trials and other applicable insurance to cover the conduct of this study.

You may be asked to sign a “release of information” form. This form will allow the research site to obtain your medical records related to the illness or injury. These records will help the Study Doctor determine the cause of the illness or injury. They may also help the Sponsor learn more about the safety of the investigational products.

If you require any further details, please ask the Study Doctor.

### **Will my taking part in this study be kept confidential?**

Information about you and your participation in this study will be kept confidential according to applicable data protection and privacy laws.

Your study records may contain your name and other personally identifiable information (PII). PII is information that identifies you. These records and your study results will be kept by the research site on paper and/or in a database as required by law or the Sponsor. This may be indefinitely so as to ensure that the Sponsor complies with, and will continue to comply with, its legal, regulatory or other governance obligations. Your study results will be coded with numbers. The research site will keep a list that links your name to your study results. This list will be kept confidential.

Your PII will be accessed by the Study Doctor and the study staff for the purposes of assessing your suitability to participate in this study, for the purposes of conducting this study, for the purposes of assessing and reporting the results of this study, for the purposes of providing you with any medical treatment you may require in connection with this study, and for the purposes of answering any questions you may have.

The Study Doctor and the study staff will process your PII in connection with HIV and Hepatitis testing as specified in this form. If you require any further information please refer to the Study Doctor.

The Study Doctor and the study staff may also disclose your PII to third parties identified below in connection with the above purposes.

The study results may also be accessed, audited and monitored by the people listed below. This is to make sure the study was done correctly and for the purposes of reviewing study results. In order for this to take place, some third parties will have direct access to and may copy some of the original records. This includes the laboratory report linking your name to your HIV and/or hepatitis report. Your original records may contain your PII.

These third parties may include:

- Regulatory authorities, including in the UK, Europe, the United States and Canada.
- The Sponsor and its affiliated companies, and third parties working with the Sponsor and its affiliated companies.
- The research site and third parties working with the research site.
- Medical practitioners providing you with any medical treatment you may require.

All of the parties listed above will maintain, use, disclose, transfer and access your PII confidentially and in accordance with applicable law. This may involve your PII being transferred, for processing as described above, to a non-EU country which does not offer the same level of protection for your PII as applies in the UK. If so, the Sponsor will take steps to ensure that any such transfer complies with data protection laws that apply within the UK.

If any or part of the study test results are published, it will be published in a way to maintain your confidentiality and in accordance with regulatory requirements.

If a medical emergency happens, your study results may be given to emergency medical staff not employed by the research site, or the Sponsor.

If you decide to stop being in the study, the information already gathered will still be kept in the study database. It will be used as described in this form. No new information for research purposes will be collected from you after the point of withdrawal.

Even though you may have consented to the use of your PII for the purposes of the study, you still have various rights under data protection law as described below (we will seek to deal with any request you make without undue delay, and in any event in accordance with the requirements of applicable laws and we may keep a record of your communications to help us resolve any issues which you raise):

• As noted above, you may withdraw your consent at any time;

• You have the right to ask us to confirm what information we hold about you at any time, and you may ask us to modify, update or delete such information. We may ask you to verify your identity, or ask for more information about your request, before acting on it. Where we are legally permitted to do so, we may decline your request, but we will explain why if so;

• In certain situations (for example, if we have processed your data unlawfully), you have the right to request us to "erase" your personal data. We will respond to your request within 30 days (although we may be allowed to extend this period in certain cases) and will only disagree with you if certain limited conditions apply;

• You also have the right to lodge a complaint with your local supervisory authority. In the UK, this is the Information Commissioner, who can be contacted at Wycliffe House, Water Lane, Wilmslow, Cheshire SK9 5AF or at casework@ico.org.uk.

Your national insurance or passport number and the date that you received your last investigational product may be entered into The Over-Volunteering Prevention System (TOPS) database so that other Clinical Research Organisations can check when you last took part in a clinical trial.

By signing this form, you are allowing the processing of your personal data as described in this form.

A description of this clinical trial will be available in accordance with with local regulatory requirements.

### **Involvement of the General Practitioner/ Family Doctor (GP)**

If you agree to take part in this study, your GP will be informed of your involvement.

### **What will happen to the samples I give?**

Your samples will not be sold or used directly for the production of commercial products.

In case of any commercial gain based on research results from your samples, the Sponsor will have the ownership of the research results. The research conducted with your samples may help to develop new products or other developments in the future that have commercial value. There will be no financial benefit to you for any commercial findings or products as a result of your participation in the study or your sample use. By agreeing to take part in this research study, you agree to give up your rights for any commercial value resulting from your samples and data.

Your samples will be provided to third party laboratories for testing, research use and storage purposes done for and on behalf of the sponsor of this study and its third party collaborators. Researchers may use samples for biomarker research to learn more about how participants respond to the investigational product. Your samples will be retained for a maximum of 15 years after completion of the study at which point any remaining samples will be destroyed.

Your samples will be coded to protect your identity and will be identified with a unique code; this will not include your initials or date of birth unless this is specifically required for medical reasons and is legally permitted.

Reports about research done with your samples will not include any details about you or be put in your health/medical record and will be kept confidential, as described above.

In the future, researchers studying your samples may need to know more information, such as whether you smoke or not, and other information such as your age, gender, race. If this information is already available because you are taking part in a study, it may be given to the researcher but it will not contain information that might reveal your identity.

At the end of the research a clinical study report will be written with the results. In the event that results are made available, it may not be possible to provide results in some circumstances.

**Freedom to refuse research on samples required by the study**

You can change your mind at any time about allowing your samples to be used for this study. If you do change your mind, contact the Study Doctor or study staff and let them know. Your samples will no longer be made available for testing and will be destroyed. If you choose not to have your samples used, then you will no longer be able to take part in this study.

(To be printed on hospital/institution headed paper)

**II. INFORMED CONSENT FORM**

| **Study Title:** A Randomised, Controlled Study to Evaluate the Effects of Switching from Cigarette Smoking to using a Tobacco Heating Product on Health Effect Indicators in Healthy Subjects. |
| --- |
| **Protocol No**.: BAT3117011 |
| **Name and Address of Sponsor:**  British American Tobacco (Investments) Ltd.  R&D Centre Regents Park Road Southampton Hampshire SO15 8TL, UK. |
| **Principal Investigator Name**: <full name> |
| **Institution:** <name and full address> |
| **IRAS No:** 230414 |

**Site Number:** <insert site number>

**Participant Identification Number for this trial:_____________________**

Sign this form ONLY if all of the following statements are true.

I hereby confirm that:

|  | Please initial boxes |
| --- | --- |
| - I have read (or someone has read to me) the information in form. I have had plenty of time to think about the purpose and procedures, the possible risks and benefits of the study. My questions have been answered to my satisfaction. |  |
| - I have been informed by Study Doctor about the purposes, type and nature of this study, what is required of me to participate in this study, and about the risks related to participation in this study. |  |
| - I agree to follow the restrictions of this study and the Study Doctor's instructions. I will tell the Study Doctor immediately if there are any changes in my health |  |
| - I am free to stop taking part in this study at any time for any reason and my choice to stop taking part will not affect my future medical care. By signing this form, I am not giving up any of my legal rights as a research participant. |  |
| - I agree to keep confidential all information relating to the investigational products, including the product design, specifications and method of operation, and I will not give the investigational products to anyone else. |  |
| - I agree that my personal and medical information will be processed as explained in this form. |  |
| - I understand that the information collected about me will be used to support other research in the future, and may be shared anonymously with other researchers. |  |
| - I agree to the study procedures and having my samples and data being used for study analysis. |  |
| - I understand that my GP will be told that I am taking part in this clinical research study |  |
| - I have decided to take part in this clinical research study. I understand I will get a signed and dated copy of this form. |  |
| - I have read (or someone has read to me) and fully understand the information stated in this form regarding HIV and Hepatitis testing. |  |
| - I believe there are no medical reasons why I should not participate in this study. I confirm that I am not pregnant or breastfeeding. |  |
| **THP/CTS Population only:** |  |
| - I confirm I am a regular smoker of between 10 and 30 cigarettes per day, and do not plan to quit smoking in the next 12 months. |  |

Please provide the following details:

| Printed Name of Participant |  |  |
| --- | --- | --- |
|  |  |  |
| Signature of Participant |  | Date and time of Signature  (dd-mmm-yyyy, hh:mm) |
|  |  |  |

| I, the undersigned, Study Doctor / study staff, confirm that I have verbally given the necessary information about the study, that I answered any additional questions, and that I did not exert any pressure on the participant to take part in the study.  I declare that I acted in full accordance with the ethical principles described in GCP Guidelines, and other national and international legislation in effect.  A copy of this form, signed by both parties, will be provided to the participant. | | |
| --- | --- | --- |
|  |  |  |
| Printed Name of Person Obtaining Consent |  |  |
|  |  |  |
| Signature of Person Obtaining Consent |  | Date and time of Signature  (dd-mmm-yyyy, hh:mm) |
